# Supplementary material for: Global seroprevalence of Zika virus in asymptomatic individuals: A systematic review
Source: PLoS Negl Trop Dis. 2024 Apr 17;18(4):e0011842. doi: 10.1371/journal.pntd.0011842 (PMC11057727; doi:10.1371/journal.pntd.0011842)
Supplement: S2 Table — (PDF) [file pntd.0011842.s002.pdf]

S2 Table. **Characteristics of Zika virus seroprevalence studies included in the systematic review and meta-analysis.**

First author	Year	Country	Region	Year of sampling	Recruitment	Design	Setting	Adults (≥13), children (<13) (age range)	% female	Diagnostic method	ZIKV Strain	Interpretation ZIKV positive	No. Positive/total (%prevalence)	Risk bias
**AFRICA**														
Tinto et al., 2022[1]	2022	Burkina Faso	Ouagadougou, Bobo-Dioulasso	2020	Blood donors	C-S	NA	Adults (18-59)	22.8	cELISA (flavivirus) ID Screen, NS1 Luminex (IgG), VNT	African: ArB41644	cELISA & VNT positive	114/501 (22.8)	L
Ward et al., 2022[2]	2022	Cabo Verde	Praia, island of Santiago	2016	General population	C-S	Urban	Adults (20-72)	63.1	NS1 ELISA Euroimmun (IgG, IgM), NS1 ELISA Native Ag. Comp. (IgG), DAB assay	NA	Positive DAB assay	47/431 (10.9)	L
Gake et al., 2017[3]	2017	Cameroon	Douala, Yaoundé, Bertoua, Ngaoundéré, Garoua, Maroua	Aug-Oct 2015	Blood donors	C-S	NA	Adults	NA	NS1 ELISA Euroimmun (IgG), VNT	Asian: OPY_Martinique_Pari_2015	ELISA ratio ≥1.1 & VNT titer≥40	53/1084 (4.9)	L
Willcox et al., 2018[4]	2018	Democratic Republic of the Congo	Nationwide	2013-2014	Children	C-S	Urban & rural	Children (0-5)	NA	Ag-capture ELISA (IgG), FRNT50	Asian: H/PF/2013	ELISA positive & FRNT> 100	1/978 (0.1)	L
Asebe et al., 2021[5]	2021	Ethiopia	Gambella Region (Lare and Itang)	2018-2019	General population	C-S	Urban	Adults (18-65)	36.0	BOB ELISA	African: Uganda	BOB positive	41/150 (27.3)	L
Ushijima et al., 2021[6]	2021	Gabon	Lambaréné, Moyen-Ogooué province	2014	General population	C-S	Semi-urban	Both (1–56)	~40.0	In-house NS1 ELISA	NA	ELISA OD≥ + 3 SD of negative OD	20/38 (52.6)	M
Ushijima et al., 2021[6]	2021	Gabon	Lambaréné, Moyen-Ogooué province	Jan-Dec 2015	General population	C-S	Semi-urban	Both (1–56)	~40.0	In-house NS1 ELISA	NA	ELISA OD≥ + 3 SD of negative OD	58/130 (44.6)	M
Ushijima et al., 2021[6]	2021	Gabon	Lambaréné, Moyen-Ogooué province	2016	General population	C-S	Semi-urban	Both (1–56)	~40.0	In-house NS1 ELISA	NA	ELISA OD≥ + 3 SD of negative OD	60/185 (32.4)	M
Ushijima et al., 2021[6]	2021	Gabon	Lambaréné, Moyen-Ogooué province	2017	General population	C-S	Semi-urban	Both (1–56)	~40.0	In-house NS1 ELISA	NA	ELISA OD≥ + 3 SD of negative OD	18/34 (52.9)	M
Sherman et al., 2018[7]	2018	Ghana	Accra	2012-2014	HIV & HBV infected	Cohort	Urban	Adults	59.0	NS1 ELISA Euroimmun (IgG, IgM), PRNT90	NA	ELISA ratio ≥1.1	31/236 (13.1)	L
Kisuya et al., 2019[8]	2019	Kenya	Nairobi, Eldoret and Kisumu	2009-2014	General population	C-S	NA	Adults	NA	NS1 ELISA Euroimmun (IgG), PRNT	African: MR766	ELISA ratio ≥1.1 & PRNT positive	1/577 (0.2)	M

Chepkorir et al., 2019[9]	2019	Kenya	West Pokot and Turkana Counties	2016-2017	General population	C-S	Rural	Adults	64.0	PRNT90	African: MR766	PRNT90 positive	34/877 (3.9)	L
Schwarz et al., 2017[10]	2017	Madagascar	Mananjary, Manakara, Ifanadiana, Tsiroanomandidiy, Moramanga, Ambositra	2010	Pregnant women	C-S	Urban	Adults	100.0	NS1 ELISA Euroimmun (IgM, IgG), IIFA, VNT	African: MR766	ELISA ratio≥1.1 & VNT positive	0/1216 (0.0)	M
Marchi et al., 2020[11]	2020	Mali	Bamako	2007	Previous vaccine clinical trial	Cohort	Urban	Both (2-29)	41.2	NS1 ELISA Euroimmun (IgG), VNT	African: MR766	ELISA ratio≥1.1 & VNT positive	1/291 (0.3)	L
Marchi et al., 2020[11]	2020	Mali	Bamako	2011-2012	Previous vaccine clinical trial	Cohort	Urban	Both (2-29)	43.9	NS1 ELISA Euroimmun (IgG), VNT	African: MR766	ELISA ratio≥1.1 & VNT positive	31/228 (13.7)	L
Diarra et al., 2020[12]	2020	Mali	Bamako	2013	Blood donors	C-S	Urban & rural	Adults (15-55)	NA	NS1 ELISA Euroimmun (IgG), VNT	Asian: H/PF/2013	ELISA ratio≥0.8 & VNT titer≥40	47/637 (7.4)	L
Diarra et al., 2020[12]	2020	Mali	Diema, Niono, Bandiagara, Kita, Bamako, Bougouni, Kadiolo	2016	General population	C-S	Urban & rural	Adults (>15)	NA	NS1 ELISA Euroimmun (IgG), VNT	Asian: H/PF/2013	ELISA ratio≥0.8 & VNT titer≥40	95/793 (11.9)	L
Mathé et al., 2018[13]	2018	Nigeria	Plateau and Nasarawa States	2016	Pregnant women and others	C-S	NA	Adults	84.0	NS1 ELISA Euroimmun (IgM, IgG), colorimetric assay	African: MR766	ELISA ratio≥1.1 & colorimetric assay in positives	48/468 (10.2)	M
Kolawole et al., 2020[14]	2020	Nigeria	Ilorin, Kwara state	2017-2018	Pregnant women	C-S	NA	Adults (18-42)	100.0	ELISA Abcam (IgG, IgM)	NA	ELISA positive	46/200 (23.0)	L
Shaibu et al., 2021[15]	2021	Nigeria	Lagos State	2018	Pregnant women	C-S	Urban	Adults (≥18)	100.0	Capture ELISA Demeditec Diag. (IgM, IgG)	NA	IgM and/or IgG positive	12/352 (3.4)	M
Anejo-Okopi et al., 2020[16]	2020	Nigeria	Jos, Plateau State	2020	Pregnant women (HIV-negative & HIV-positive)	C-S	Urban	Adults (≥18 and ≤50)	100.0	ELISA My BioSource Inc (IgG)	NA	ELISA D.O. >0.172	13/90 (14.4)	L
Mac et al., 2023[17]	2023	Nigeria	Nasarawa, Abia, and Kaduna State	2020-2021	Pregnant women, HIV-positive, blood donors	C-S	Urban, rural, & slums	Both (0-80)	71.0	NS1 and Equad immunoblot Mikrogen Diagnostik (IgG)	NA	Immunoblot positive	167/871 (19.2)	L
Nurtop et al., 2019[18]	2019	Republic of the Congo	Brazzaville, Pointe-Noire, and rural areas (Ewo, Gamboma, Oyo, Owando)	2011	Blood donors	C-S	Urban & rural	Adults (18-63)	21.5	NS1 ELISA Euroimmun (IgG), VNT	Asian: H/PF/2013	ELISA ratio≥0.8 & VNT≥40	7/386 (1.8)	L

Seruyange et al., 2018[19]	2018	Rwanda	Eastern, Western, Northern, Southern provinces & Kigali city	2015 (no month)	Blood donors	C-S	NA	Adults (17-63)	34.7	NS1 ELISA Euroimmun (IgG)	NA	ELISA ratio≥1.1	12/874 (1.4)	L
Marchi et al., 2020[11]	2020	Senegal	Niakhar, Fatick region	2007	Previous vaccine clinical trial	Cohort	Rural	Both (2-29)	44.4	NS1 ELISA Euroimmun (IgG), VNT	African: MR766	ELISA ratio≥1.1 & VNT positive	38/284 (13.4)	L
Marchi et al., 2020[11]	2020	Senegal	Niakhar, Fatick region	2011-2012	Previous vaccine clinical trial	Cohort	Rural	Both (2-29)	47.4	NS1 ELISA Euroimmun (IgG), VNT	African: MR766	ELISA ratio≥1.1 & VNT positive	15/211 (7.1)	L
Soghaier et al., 2018[20]	2018	Sudan	Northern, River Nile, Red Sea, Khartoum	2012	General population	C-S	Urban & rural	Both (2-92)	53.0	In-house ELISA, PRNT90	African: MR766	ELISA & PRNT positive	1/1722 (0.05)	L
Mwanyika et al., 2021[21]	2021	Tanzania	Buhigwe, Kalambo, Kilindi, Kinondoni, Kondoa, Kyela, Mvomero, and Ukerewe	2018	General population & people who attended healthcare facilities	C-S	Urban & rural	Both	54.4	NS1 ELISA Euroimmun (IgG)	NA	ELISA ratio≥1.1	121/1818 (6.8)	L
Marchi et al., 2020[11]	2020	The Gambia	Basse Santa Su	2007	Previous vaccine clinical trial	Cohort	Rural	Both (2-29)	39.5	NS1 ELISA Euroimmun (IgG), VNT	African: MR766	ELISA ratio≥1.1 & VNT positive	11/296 (3.7)	L
Marchi et al., 2020[11]	2020	The Gambia	Basse Santa Su	2011-2012	Previous vaccine clinical trial	Cohort	Rural	Both (2-29)	39.3	NS1 ELISA Euroimmun (IgG), VNT	African: MR766	ELISA ratio≥1.1 & VNT positive	1/211 (0.4)	L
Chisenga et al., 2020[22]	2020	Zambia	Lukanga swamp, Central Province	2016	General population	C-S	Rural	Adults (≥18)	8.4	In-house NS1 ELISA	NA	ELISA D.O. >0.8	23/214 (10.8)	L
Babaniyi et al., 2015[23]	2015	Zambia	Western and Northern Province	NA	NA	C-S	NA	Both	53.3	IgM and IgG (NA test)	NA	NA	217/3579 (6.1)	M
**AMERICA**														
Saba Villarroel et al., 2018[24]	2018	Bolivia	La Paz, Cochabamba, Santa Cruz, Beni & Tarija	2016-2017	Blood donors	C-S	Urban	Adults	42.3	NS1 ELISA Euroimmun (IgG), VNT	Asian: H/PF/2013	ELISA ratio≥0.8 & VNT≥40	237/814 (29.0)	L
Slavov et al., 2020[25]	2020	Brazil	Ribeirão Preto, São Paulo State	Feb-Apr 2015	Blood donors	C-S	Urban	Adults	NA	NS1 ELISA Euroimmun (IgG), PRNT	NA	ELISA ratio ≥1.1 & PRNT	0/93 (0.0)	L
Salgado et al., 2021[26]	2021	Brazil	Manaus, Amazonas state	2014-Dec 2015	Military personnel	C-S	NA	Adults (20-49)	0.0	HIA	NA	HIA titre≥ 1:20	92/298 (30.9)	L
Batista Salgado et al., 2023[27]	2023	Brazil	Manaus, Amazonas state	Jan-Dec 2015	General population	C-S	Urban	Adults (≥18)	70.5	HIA, EDIII ELISA (IgG), FRNT50	Asian: BeH823339	HIA titre ≥1:20	235/447 (52.6)	L

Netto et al., 2017[28]	2017	Brazil	Salvador, Bahia state	Nov 2015-2016	HIV-positive, mothers, TB patients & university employees	C-S	Urban	Adults	69.0	NS1 ELISA Euroimmun (IgG), PRNT, E-based In-house ELISA	Asian: H/PF/2013	ELISA ratio≥1.1	401/633 (63.3)	L
Moreira-Soto et al., 2020[29]	2020	Brazil	Salvador, Bahia state	2016	HIV-positive, TB patients & general population	C-S	Urban	Adults	NA	NS1 ELISA Euroimmun (IgG), PRNT	Asian: H/PF/2013	ELISA ratio≥1.1	85/144 (59.0)	L
Slavov et al., 2020[25]	2020	Brazil	Ribeirão Preto, São Paulo State	2016	Blood donors	C-S	Urban	Adults	NA	NS1 ELISA Euroimmun (IgG), PRNT	NA	ELISA ratio≥1.1 & PRNT positive	19/320 (5.6)	L
Alves et al., 2020[30]	2020	Brazil	Recife, Pernambuco State	2016-2017	Pregnant women	C-S	Urban	Adults	100.0	NS1 ELISA Euroimmun (IgG, IgM)	NA	ELISA ratio ≥1.1	81/132 (61.3)	L
Diefenbach et al., 2019[31]	2019	Brazil	Santa Maria, Rio Grande do Sul State	2016-2017	Blood donors	C-S	Urban	Adults	36.2	NS1 ELISA Euroimmun (IgG)	NA	ELISA ratio ≥1.1	1/182 (0.6)	L
Slavov et al., 2020[25]	2020	Brazil	Ribeirão Preto, São Paulo State	2017	Blood donors	C-S	Urban	Adults	NA	NS1 ELISA Euroimmun (IgG), PRNT	NA	ELISA ratio≥1.1 & PRNT positive	29/317 (9.1)	L
Wittlin et al., 2021[32]	2021	Brazil	Rio de Janeiro State, municipality of Nova Iguaçu	2017	Pregnant women	C-S	Urban	Adults (18-47)	100.0	NS1 ELISA Euroimmun (IgG), ELISA capture Novagnost (IgM)	NA	ELISA ratio IgM positive and/or IgG ratio≥1.1	169/349 (48.2)	M
Moreira-Soto et al., 2020[29]	2020	Brazil	Salvador, Bahia state	2017-2018	HIV-positive, TB patients & general population	C-S	Urban	Adults	NA	NS1 ELISA Euroimmun (IgG), PRNT	Asian: H/PF/2013	ELISA ratio≥1.1	56/144 (38.6)	L
de Almeida Barreto et al., 2020[33]	2020	Brazil	Juazeiro do Norte, Ceará State	2018	General population	C-S	Urban	Both (5-91)	68.3	NS1 ELISA Euroimmun (IgG), ELISA capture, Novagnostic (IgM)	NA	ELISA positive	225/404 (55.7)	L
Santos Périssé et al., 2020[34]	2020	Brazil	Rio de Janeiro State	2018	General population	C-S	Urban	Both	59.5	RDT Bio-Manguinhos (IgM/IgG)	NA	RDT positive	1080/2120 (50.9)	L
Braga et al., 2023[35]	2023	Brazil	Recife, Pernambuco State	2018-2019	General population	C-S	Urban	Both (5-65)	58.6	NS1 ELISA Euroimmun (IgG), NS1 in-house IgG3 antibodies, PRNT50	Asian: BR-PE243/2015	ELISA ratio ≥1.35, PRNT≥1:100 (Bayesian method)	1043/2070 (50.4)	L
Francisco et al., 2020[36]	2020	Brazil	Conde, Bahia state	2019	General population	C-S	Peri-urban & rural	Both	NA	ELISA IgG (NA test)	NA	ELISA positive	71/336 (21.2)	M
Maria de Araújo et al., 2023[37]	2023	Brazil	Bahia state	2019	Healthcare workers	C-S	Urban & rural	Adults (>20)	82.8	DPP® ZDC IgM/IgG rapid test	NA	Rapid test positive	177/434 (41.7)	L

										Biomanguinhos (IgM/IgG)				
Bayona-Pacheco et al., 2019[38]	2019	Colombia	Barranquilla, Atlantic Department	May-Aug 2015	Blood donors	C-S	Urban	Adults	NA	NS1 ELISA Euroimmun (IgG)	NA	ELISA ratio≥1.1	26/390 (6.7)	L
Marbán Castro et al., 2020[39]	2020	Colombia	Cereté, Córdoba Department	2016	Pregnant women	C-S	Rural	Adults	100.0	NS1 ELISA Euroimmun (IgG, IgM)	NA	ELISA ratio≥1.1	80/90 (89.0)	L
Rivas et al., 2022[40]	2022	Colombia	Cali, Valle del Cauca Department	2017-2018	General population	C-S	NA	Adults (15-40)	NA	NS1 BOB ELISA, VNT	Asian: Suriname strain	ELISA titers ≥10, VNT titer ≥100	319/700 (45.5)	M
Cardona-Ospina et al., 2022[41]	2022	Colombia	La Virginia and Pereira, Risaralda Department	2017-2019	Pregnant women	C-S	Urban	Adults (18-35)	100.0	Ag-capture anti–E protein ELISA, FRNT50, eFRNT	Asian: H/PF/2013	ELISA OD D≥ + 3 SD of negative OD + 0.1 & FRNT50 positive	99/115 (86.8)	L
Flamand et al., 2019[42]	2019	French Guiana	22 municipalities	2017	General population	C-S	Urban & rural	Both (2–75)	58.9	E3 MIA, VNT	Asian: French Guiana	MIA ratio >2.5 & VNT titer >20	628/2697 (23.3)	L
Lamb et al., 2022[43]	2022	Guatemala	25 communities, southwest lowlands	Oct–Nov 2015	Children	C-S	Rural	Children	~50.0	NS1 BOB ELISA, VNT	Asian: Suriname	ELISA >10 & VNT50 ≥100	20/196 (10.2)	L
Lamb et al., 2022[43]	2022	Guatemala	25 communities, southwest lowlands	2016	Children	C-S	Rural	Children	~50.0	NS1 BOB, VNT	Asian: Suriname	ELISA>10 & VNT50≥100	69/186 (37.1)	L
Rivas et al., 2022[40]	2022	Honduras	Tegucigalpa	2017-2018	General population	C-S	NA	Adults (15-40)	NA	NS1 BOB ELISA, VNT	Asian: Suriname, 2015	ELISA titer≥10, VNT titer≥100	369/500 (73.8)	M
Anzinger et al., 2022[44]	2022	Jamaica	Kingston	2017–2019	Pregnant women	C-S	Urban	Adults (>16)	100.0	ELISA BLACKBOX	NA	ELISA positive	91/584 (15.6)	L
Gallian et al., 2017[45]	2017	Martinique	NA	2016	Blood donors	C-S	Urban	Adults	45.4	NS1 ELISA Euroimmun (IgG), VNT	Asian: OPY_Martinique_Paris_2015	ELISA ratio ≥1.1 & VNT titer≥40	130/594 (21.9)	L
Rivas et al., 2022[40]	2022	Mexico	Acapulco, Guerrero and Temixco, Morelos	2017-2018	General population	C-S	NA	Adults (15-40)	NA	NS1 BOB ELISA, VNT	Asian: Suriname, 2015	ELISA titer ≥10, VNT titer ≥100	477/800 (59.6)	M
Eligio-García et al., 2020[46]	2020	Mexico	Tuxtla Gutiérrez, Chiapas	2019	Pregnant women	C-S	Urban	Adults (14-43)	100.0	NS1 ELISA Euroimmun (IgG)	NA	ELISA ratio≥1.1	85/136 (62.5)	L
Collins et al., 2020[47]	2020	Nicaragua	León	2016	Pregnant women	Cohort	Urban	Adults	100.0	Ag-capture ELISA (IgG), MAC ELISA (IgM), NS1 BOB, FRNT50, eFRNT	Asian: H/PF/2013	ELISA and eFRNT pos	110/187 (59.0)	M
Zambrana et al., 2018[48]	2018	Nicaragua	Managua	2017	General population	C-S	Urban	Both (2-80)	44.5	NS1 BOB ELISA	NA	ELISA positive	2338/5887 (39.8)	L

Zepeda et al., 2023[49]	2023	Nicaragua	León	2017-2018	Pregnant women	Cohort	Urban	Adults	100.0	Ag-capture ELISA (IgG), EDIII IgG, FRNT50	Asian: H/PF/2013	ELISA D.O.>0.34	171/236 (72.5)	L
Cachay et al., 2022[50]	2022	Peru	Pueblo Nuevo and Chincha Baja, Chincha province	2019	General population	C-S	Urban	Adults (20-40)	78.8	NS1 ELISA Euroimmun (IgG), VNT	Asian: H/PF/2013	ELISA ratio≥0.8 & VNT≥40	52/400 (13.0)	L
Rivas et al., 2022[40]	2022	Puerto Rico	Carolina	2017-2018	General population	C-S	NA	Adults (15-40)	NA	NS1 BOB ELISA, VNT	Asian: Suriname, 2015	ELISA titer ≥10, VNT titer ≥100	136/400 (34.0)	M
Langerak et al., 2019[51]	2019	Suriname	Paramaribo, Laduani, Kwamalasamutu	2017	People who attended healthcare facilities	C-S	Urban & rural	Adults (≥18)	59.1	NS1 ELISA Euroimmun (IgG), VNT	Asian: ZIKVNL00013	ELISA ratio ≥1.1, VNT titer≥1:32	270/770 (35.1)	L
**EASTERN MEDITERRANEAN**														
Ziyaeyan et al., 2018[52]	2018	Iran	Hormozgan province	2016-2017	People who attended laboratory services	C-S	Urban & rural	Both (>0)	76.3	NS1 ELISA Euroimmun (IgG)	NA	ELISA ratio ≥1.1	0/494 (0.0)	M
Sinbat et al., 2021[53]	2021	Iraq	Basrah city	2019-2020	General population	C-S	Urban & rural	Both (10-80)	61.7	Capture MAC-ELISA (IgM) & E3 ELISA (IgG) DxSelectTM	NA	ELISA positive	13/108 (12.0)	M
Alayed et al., 2018[54]	2018	Saudi Arabia	Najran city	2016-2017	Pregnant women	C-S	Urban	Adults	100.0	ELISA MyBiosurce (IgM, IgG)	NA	ELISA positive	76/410 (18.5)	L
**EUROPE**														
Abushoufa et al., 2021[55]	2021	Cyprus	Nicosia	2019	Blood donors	C-S	Urban	Adults (18-90)	34.0	NS1 ELISA R&D Systems (IgG)	NA	ELISA OD≥0.200	0/91 (0.0)	M
Franke et al., 2020[56]	2020	France	Hyeres, Provence-Alpes-Côte d'Azur	2019	General population	C-S	Urban	NA	NA	ELISA flavivirus (IgM/IgG), VNT	Asian: NA	ELISA positive & VNT positive	0/89 (0.0)	L
Seruyange et al., 2018[19]	2018	Sweden	NA	2015	Blood donors	C-S	NA	Adults (18-75)	54.0	NS1 ELISA Euroimmun (IgG)	NA	ELISA ratio≥1.1	0/215 (0.0)	L
**SOUTHEAST ASIA**														
Sasmono et al., 2021[57]	2021	Indonesia	Multiple sites (30 sites in 14 provinces)	2014	Children	C-S	Urban	Children (5-9)	NA	PRNT90	Asian: JMB-185	PRNT90 titer>1:10 or ≥4-fold> all DENV	80/870 (9.2)	L
Sasmono et al., 2018[58]	2018	Indonesia	Multiple sites (30 sites in 14 provinces)	2014	Children	C-S	Urban	Children (1-4)	NA	PRNT90	Asian: JMB-185	PRNT90 titer>1:10 or ≥4-fold> all DENV	60/662 (9.1)	L
Harapan et al., 2022[59]	2022	Indonesia	Aceh province	2017	General population	C-S	NA	Both (4-67)	64.8	PRNT90	Asian: PRVABC59	PRNT90 titer>1:10 or	0/116 (0.0)	L

											≥4-fold> all DENV		
Choyrum et al., 2022[60]	2022	Thailand	Nationwide	1997-2000	Pregnant women (HIV-negative, HIV, HBV or HCV-infected)	C-S	NA	Adults (18-25)	100.0	NS1 ELISA Euroimmun (IgG)	NA	ELISA ratio≥1.1	191/786 (24.3)	L
Choyrum et al., 2022[60]	2022	Thailand	Nationwide	2001-2003	Pregnant women (HIV-negative, HIV, HBV or HCV-infected)	C-S	NA	Adults (18-25)	100.0	NS1 ELISA Euroimmun (IgG)	NA	ELISA ratio≥1.1	68/248 (27.4)	L
Choyrum et al., 2022[60]	2022	Thailand	Nationwide	2008–2011	Pregnant women (HIV-negative, HIV-positive, HBV-infected, HCV-infected)	C-S	NA	Adults (18-25)	100.0	NS1 ELISA Euroimmun (IgG)	NA	ELISA ratio≥1.1	25/102 (24.5)	L
Yamanaka et al. 2021[61]	2021	Thailand	Nakhon Sawan, Uthai Thani, Rayoung, Phuket provinces	2011-2012	General population	C-S	Urban	Both (10-59)	NA	SRIP neutralization system	NA	SRIP IC75 titer >4-fold > DENV and JEV	25/147 (17.0)	M
Chakma et al., 2022[62]	2022	Thailand	Ratchaburi province	2012	General population	C-S	Urban	Both (1-55)	50.0	In-house NS1 ELISA (IgG), RDT Chembio DPP® ZCD (IgM/IgG)	NA	ELISA OD ≥ twice over the neg control	232/400 (58.0)	M
Choyrum et al., 2022[60]	2022	Thailand	Nationwide	2012–2014	Pregnant women (HIV-negative; HIV, HBV, or HCV-infected)	C-S	NA	Adults (18-25)	100.0	NS1 ELISA Euroimmun (IgG)	NA	ELISA ratio≥1.1	30/113 (26.5)	L
Choyrum et al., 2022[60]	2022	Thailand	Nationwide	2015–2017	Pregnant women (HIV-negative; HIV, HBV, or HCV-infected), young men	C-S	NA	Adults (18-25)	56.4	NS1 ELISA Euroimmun (IgG)	NA	ELISA ratio≥1.1	66/399 (16.5)	L
Sornjai et al., 2018[63]	2018	Thailand	Nakhon Pathom province	2017	General population	C-S	Urban	Adults (18-63)	82.2	PRNT50, PRNT90	Asian: SV0010/15	PRNT90 titer≥ 20	30/135 (22.2)	L
Sirinam et al., 2022[64]	2022	Thailand	Samut Songkhram province	2017-2018	General population	Cohort	NA	Both (5-50)	NA	NS1 ELISA (IgG), NS1 BOB ELISA, PRNT50	Asian: SV0127/14	ELISA ratio>2 & ZIKV/DENV NS1 P/N ratio>2; seroconversion 2fold increase.	53/350 (15.1)	M

Densathaporn et al., 2020[65]	2020	Thailand	Surat Thani & Narathiwat province	2018-2019	General population & pregnant women	C-S	Urban	Adults (>18)	NA	PRNT90	African: MR766	PRNT90 titer≥1:10	418/1567 (26.7)	L
Phatihattakorn et al. 2021[66]	2021	Thailand	Multiple provinces	2019	Pregnant women	C-S	Urban	Adults (18-45)	100.0	NS1 ELISA Euroimmun (IgG), FRNT50	NA	ELISA ratio ≥1.1, FRNT titer≥10	200/650 (30.8)	L
**WESTERN PACIFIC**														
Sun et al., 2019[67]	2019	China	Guangdong province	2016	General population	C-S	NA	Both (1-95)	58.2	VNT	NA	VNT positive	1/665 (0.1)	L
Li et al., 2019[68]	2019	China	Guizhou province	2017	General population	C-S	Rural	Both (>0)	53.2	PRNT90	Asian: GZDJ1685	PRNT90 titer≥1:10 4-fold> JEV	0/366 (0.0)	M
Zhou et al., 2020[69]	2020	China	Guangxi province	2019	General population	C-S	Urban	Adults (20-57)	42.0	NS1 ELISA Wending Biotech (IgM, IgG), VNT	Clinical isolate (Ecuador)	ELISA OD>0.748 & VNT titer≥22.3	17/273 (6.2)	L
Henderson et al., 2020[70]	2020	Fiji	Central Division	2013	General population	C-S	NA	Both (2–78)	NA	MIA, VNT	Asian: H/PF/2013	MIA positive	12/189 (6.3)	L
Kama et al., 2019[71]	2019	Fiji	Northern, Western and Central divisions	2013	General population	Cohort	Urban & rural	Both	NA	MIA, VNT	Asian: MIA: H/PF/2013 VNT: PF13/2510 13-18	MIA positive	61/778 (7.8)	L
Kama et al., 2019[71]	2019	Fiji	Central Division	Oct-Nov 2015	General population	Cohort	Urban & rural	Both	56.9	MIA, VNT	Asian: MIA: H/PF/2013 VNT: PF13/2510 13-18	MIA positive	69/311 (22.2)	L
Henderson et al., 2020[70]	2020	Fiji	Central Division	Nov-2015	General population	C-S	NA	Both (4–80)	NA	MIA, VNT	Asian: H/PF/2013	MIA positive	45/189 (23.8)	L
Henderson et al., 2020[70]	2020	Fiji	Central Division	2017	General population	C-S	NA	Both (6–82)	NA	MIA, VNT	Asian: H/PF/2013	MIA positive	23/189 (12.2)	L
Aubry et al., 2015[72]	2015	French Polynesia	Society Island	2011-2013	Blood donors	C-S	Urban	Adults (18-75)	NA	E3 ELISA (IgG)	Asian: H/PF/2013	ELISA positive	5/593 (0.8)	L
Henderson et al., 2020[70]	2020	French Polynesia	Society Islands	2014	General population	C-S	NA	Both (6-77)	NA	E3 ELISA (IgG)	Asian: H/PF/2013	ELISA positive	330/525 (62.9)	L
Cauchemez et al., 2016[73]	2016	French Polynesia	Society, Tuamotu, Marquesas, Australs, and Gambier	2014	General population	C-S	NA	Both (7-86)	NA	E3 ELISA (IgG), MIA	Asian: H/PF/2013	ELISA or MIA positive	97/196 (49.5)	M

Cauchemez et al., 2016[73]	2016	French Polynesia	Society Islands	2014	Children and adolescents	C-S	NA	Both (6-16)	NA	E3 ELISA (IgG)	Asian: H/PF/2013	ELISA positive	314/476 (66.0)	M
Aubry et al., 2017[74]	2017	French Polynesia	Society Islands	Sep-Nov 2015	General population	C-S	NA	Both (4–88)	NA	MIA	Asian: H/PF/2013	MIA positive	154/700 (22.0)	M
Henderson et al., 2020[70]	2020	French Polynesia	Society Islands	2018	Children and adolescents	C-S	NA	Both (6-16)	NA	MIA	Asian: H/PF/2013	MIA positive	291/457 (63.7)	L
Pastorino et al. 2019[75]	2019	Lao PDR	Vientiane	2003-2004	Blood donors	C-S	Urban	Adults (16-63)	20.0	NS1 ELISA Euroimmun (IgG), VNT	Asian: OPY_Martinique_Pari_2015	ELISA ratio≥0.8 & VNTtiter≥40	16/359 (4.5)	L
Pastorino et al. 2019[75]	2019	Lao PDR	Vientiane	2015	Blood donors	C-S	Urban	Adults (17-79)	37.3	NS1 ELISA, Euroimmun (IgG), VNT	Asian: MRS-OPY_Martinique_Pari_2015	ELISA ratio≥0.8 & VNT titer≥40	68/687 (9.9)	L
Sam et al., 2019[76]	2019	Malaysia	Kuala Lumpur	2014-Mar 2015	Blood donors	C-S	Urban	Adults	~40.0	NS1 BOB ELISA, PRNT50, FRNT50	Asian: OPY_Martinique_Pari_2015	NS1 BOB reactive & PRNT≥20 or FRNT ≥4-fold> all DENV	3/178 (1.7)	M
Sam et al., 2019[76]	2019	Malaysia	Kuala Lumpur	2017	Blood donors	C-S	Urban	Adults	~40.0	NS1 BOB, PRNT50, FRNT50	Asian: MRS-OPY_Martinique_Pari_2015	NS1 BOB reactive & PRNT≥20 or FRNT≥4-fold> all DENV	1/181 (0.6)	M
Khoo et al., 2022[77]	2022	Malaysia	Perak, Pahang, and Sabah	2019-2020	General population	C-S	Rural & semiurban	Adults (>18)	69.1	NS1 ELISA Euroimmun (IgG), FRNT90	Asian: P6-740	ELISA ratio≥1.1 & FRNT titer ≥1:40	148/ 706 (21.0)	L
Khor et al., 2020[78]	2020	Malaysia	Forest fringe areas of Peninsular Malaysia	NA	Indigenous people	C-S	Rural	Both (>5)	57.1	NS1 ELISA Euroimmun (IgG), FRNT50	NA	ELISA ratio≥1.1 & FRNT titer≥ 1:40	115/872 (13.2)	L
Grant et al., 2022[79]	2022	Papua New Guinea	Manus Island, Wewak	2019	Military personnel	C-S	NA	Adults (20-62)	0.5	NS1 ELISA Euroimmun (IgG, IgM), VNT	Asian: MR766	VNT positive	135/208 (64.9)	L
Russell et al., 2022[80]	2022	Solomon Islands	Honiara, North Guadalcanal, Isabel, East Malaita and West Malaita	2018	General population	C-S	Rural	Both (5-86)	63.6	NS1 ELISA Euroimmun (IgG)	NA	ELISA ratio ≥1.1	287/1021 (28.1)	L
Chien et al., 2019[81]	2019	Taiwan	Tainan City	End of 2015	General population	C-S	Urban	Adults (>20)	67.0	NS1 ELISA Euroimmun (IgG, IgM), PRNT90	African and Asian: MR766 and clinical	ELISA ratio ≥1.1 & PRNT≥4-fold> all DENV	1/212 (0.5)	L

											isolate (Thailand)			
Adams et al., 2021[82]	2021	The Philippines	Cebu Province	2017	Children	C-S	Semi-urban	Children (9-14)	NA	EDIII-capture ELISA, FRNT50	Asian: H/PF/2013	ELISA OD>0.34	98/547 (18.0)	L
Chiu et al., 2023[83]	2023	Vietnam	Ho Chi Minh City	2008	Pregnant women	C-S	Urban	Adults	100.0	NS1 ELISA Euroimmun (IgG), IIFT Euroimmun, VNT	Asian: PRVABC59	ELISA & VNT >4fold 1DENV, JEV, WNV, YFV	1/176 (0.6)	L
Nguyen et al., 2020[84]	2020	Vietnam	Dak Lak Province	2017-2018	Pregnant women & general population	C-S	Rural	Both	73.7	In-house capture ELISA (IgM), PRNT50, NS1 ELISA R&D Systems (IgG)	African: MR766	ELISA positive &/or PRNT50 titer ≥20 & IgG pos & ≥4fold >all DENV	9/801 (1.1)	L

Abbreviations: BOB: Blockade-of-binding assay; cELISA: competitive enzyme-linked immunosorbent assay; C-S: cross-sectional; DAB: double antigen binding; DENV: Dengue virus; EDIII: Domain III of the ZIKV E protein; ELISA: Enzyme-linked immunosorbent assay; FRNT: Focus reduction neutralization assay; HIA: hemagglutination assay; IIFT: Indirect immunofluorescence; JEV: Japanese Encephalitis virus; L: Low; MIA: Microsphere immunoassay; M: Moderate; NA: No data available; NS1: nonstructural protein 1; RDT: Rapid diagnostic test; SD: Standard Deviation; SRIP: Single round infectious particles; PRNT: Plaque reduction neutralization test; VNT: Virus neutralization test; WNV: West Nile virus; YFV: Yellow Fever virus

References:

1. Tinto B, Kaboré DPA, Kania D, Kagoné TS, Kiba-Koumaré A, Pinceloup L, et al. Serological Evidence of Zika Virus Circulation in Burkina Faso. Pathogens. 2022;11: 741. doi:10.3390/pathogens11070741

2. Ward D, Gomes AR, Tetteh KKA, Sepúlveda N, Gomez LF, Campino S, et al. Sero-epidemiological study of arbovirus infection following the 2015–2016 Zika virus outbreak in Cabo Verde. Sci Rep. 2022;12: 11719. doi:10.1038/s41598-022-16115-4

3. Gake B, Vernet MA, Leparc-Goffart I, Drexler JF, Gould EA, Gallian P, et al. Low seroprevalence of Zika virus in Cameroonian blood donors. Braz J Infect Dis. 2017;21: 481–483. doi:10.1016/j.bjid.2017.03.018

4. Willcox AC, Collins MH, Jadi R, Keeler C, Parr JB, Mumba D, et al. Seroepidemiology of Dengue, Zika, and Yellow Fever Viruses among Children in the Democratic Republic of the Congo. Am J Trop Med Hyg. 2018;99: 756–763. doi:10.4269/ajtmh.18-0156

5. Asebe G, Michlmayr D, Mamo G, Abegaz WE, Endale A, Medhin G, et al. Seroprevalence of Yellow fever, Chikungunya, and Zika virus at a community level in the Gambella Region, South West Ethiopia. PLoS ONE. 2021;16. doi:10.1371/journal.pone.0253953

6. Ushijima Y, Abe H, Nguema Ondo G, Bikangui R, Massinga Loembé M, Zadeh VR, et al. Surveillance of the major pathogenic arboviruses of public health concern in Gabon, Central Africa: increased risk of West Nile virus and dengue virus infections. BMC Infect Dis. 2021;21: 265. doi:10.1186/s12879-021-05960-9

7. Sherman KE, Rouster SD, Kong LX, Shata TM, Archampong T, Kwara A, et al. Zika Virus Exposure in an HIV-Infected Cohort in Ghana. J Acquir Immune Defic Syndr. 2018;78: e35–e38. doi:10.1097/QAI.0000000000001718

8. Kisuya B, Masika MM, Bahizire E, Oyugi JO. Seroprevalence of Zika virus in selected regions in Kenya. Transactions of The Royal Society of Tropical Medicine and Hygiene. 2019;113: 735–739. doi:10.1093/trstmh/trz077

9. Chepkorir E, Tchouassi DP, Konongoi SL, Lutomiah J, Tigoi C, Irura Z, et al. Serological evidence of Flavivirus circulation in human populations in Northern Kenya: an assessment of disease risk 2016-2017. Virol J. 2019;16: 65. doi:10.1186/s12985-019-1176-y

10. Schwarz NG, Mertens E, Winter D, Maiga-Ascofaré O, Dekker D, Jansen S, et al. No serological evidence for Zika virus infection and low specificity for anti-Zika virus ELISA in malaria positive individuals among pregnant women from Madagascar in 2010. PLoS One. 2017;12: e0176708. doi:10.1371/journal.pone.0176708

11. Marchi S, Viviani S, Montomoli E, Tang Y, Boccuto A, Vicenti I, et al. Zika Virus in West Africa: A Seroepidemiological Study between 2007 and 2012. Viruses. 2020;12: 641. doi:10.3390/v12060641

12. Diarra I, Nurtop E, Sangaré AK, Sagara I, Pastorino B, Sacko S, et al. Zika Virus Circulation in Mali. Emerg Infect Dis. 2020;26: 945–952. doi:10.3201/eid2605.191383

13. Mathé P, Egah DZ, Müller JA, Shehu NY, Obishakin ET, Shwe DD, et al. Low Zika virus seroprevalence among pregnant women in North Central Nigeria, 2016. Journal of Clinical Virology. 2018;105: 35–40. doi:10.1016/j.jcv.2018.05.011

14. Kolawole OM, Suleiman MM, Bamidele EP. Molecular epidemiology of Zika virus and Rubella virus in pregnant women attending Sobi Specialist Hospital Ilorin, Nigeria. International Journal of Research in Medical Sciences. 2020;8: 2275–2283. doi:10.18203/2320-6012.ijrms20202234

15. Shaibu JO, Okwuraiwe AP, Jakkari A, Dennis A, Akinyemi KO, Li J, et al. Sero-molecular Prevalence of Zika Virus among Pregnant Women Attending Some Public Hospitals in Lagos State, Nigeria. European Journal of Medical and Health Sciences. 2021;3: 77–82. doi:10.24018/ejmed.2021.3.5.1075

16. Anejo-Okopi J, Gotom DY, Chiehiura NA, Okojokwu JO, Amanyi DO, Egbere JO, et al. The Seroprevalence of Zika Virus Infection among HIV Positive and HIV Negative Pregnant Women in Jos, Nigeria. Hosts and Viruses. 637134336000000000;7. doi:10.17582/journal.hv/2020/7.6.129.136

17. Mac PA, Kroeger A, Daehne T, Anyaike C, Velayudhan R, Panning M. Zika, Flavivirus and Malaria Antibody Cocirculation in Nigeria. Tropical Medicine and Infectious Disease. 2023;8: 171. doi:10.3390/tropicalmed8030171

18. Nurtop E, Moyen N, Dzia-Lepfoundzou A, Dimi Y, Ninove L, Drexler JF, et al. A Report of Zika Virus Seroprevalence in Republic of the Congo. Vector Borne Zoonotic Dis. 2020;20: 40–42. doi:10.1089/vbz.2019.2466

19. Seruyange E, Gahutu J-B, Muvunyi CM, Katare S, Ndahindwa V, Sibomana H, et al. Seroprevalence of Zika virus and Rubella virus IgG among blood donors in Rwanda and in Sweden. J Med Virol. 2018;90: 1290–1296. doi:10.1002/jmv.25198

20. Soghaier MA, Abdelgadir DM, Abdelkhalig SM, Kafi H, Zarroug IMA, Sall AA, et al. Evidence of pre-existing active Zika virus circulation in Sudan prior to 2012. BMC Res Notes. 2018;11: 906. doi:10.1186/s13104-018-4027-9

21. Mwanyika GO, Sindato C, Rugarabamu S, Rumisha SF, Karimuribo ED, Misinzo G, et al. Seroprevalence and associated risk factors of chikungunya, dengue, and Zika in eight districts in Tanzania. Int J Infect Dis. 2021;111: 271–280. doi:10.1016/j.ijid.2021.08.040

22. Chisenga CC, Bosomprah S, Musukuma K, Mubanga C, Chilyabanyama ON, Velu RM, et al. Sero-prevalence of arthropod-borne viral infections among Lukanga swamp residents in Zambia. PLoS One. 2020;15: e0235322. doi:10.1371/journal.pone.0235322

23. Babaniyi OA, Mwaba P, Songolo P, Mazaba-Liwewe ML, MweeneNdumba I, Masaninga F, et al. Seroprevalence of Zika virus infection specific IgG in Western and North-Western Provinces of Zambia. International Journal of Public Health and Epidemiology. 2014;4: 1–6.

24. Saba Villarroel PM, Nurtop E, Pastorino B, Roca Y, Drexler JF, Gallian P, et al. Zika virus epidemiology in Bolivia: A seroprevalence study in volunteer blood donors. PLoS Negl Trop Dis. 2018;12: e0006239. doi:10.1371/journal.pntd.0006239

25. Slavov SN, Guaragna Machado RR, Ferreira AR, Soares CP, Araujo DB, Leal Oliveira DB, et al. Zika virus seroprevalence in blood donors from the Northeastern region of São Paulo State, Brazil, between 2015 and 2017. Journal of Infection. 2020;80: 111–115. doi:10.1016/j.jinf.2019.10.002

26. Salgado BB, de Jesus Maués FC, Pereira RL, Chiang JO, de Oliveira Freitas MN, Ferreira MS, et al. Prevalence of arbovirus antibodies in young healthy adult population in Brazil. Parasit Vectors. 2021;14: 403. doi:10.1186/s13071-021-04901-4

27. Salgado BB, Maués FC de J, Jordão M, Pereira RL, Toledo-Teixeira DA, Parise PL, et al. Antibody cross-reactivity and evidence of susceptibility to emerging Flaviviruses in the dengue-endemic Brazilian Amazon. Int J Infect Dis. 2023;129: 142–151. doi:10.1016/j.ijid.2023.01.033

28. Netto EM, Moreira-Soto A, Pedroso C, Höser C, Funk S, Kucharski AJ, et al. High Zika Virus Seroprevalence in Salvador, Northeastern Brazil Limits the Potential for Further Outbreaks. mBio. 2017;8: e01390-17. doi:10.1128/mBio.01390-17

29. Moreira-Soto A, de Souza Sampaio G, Pedroso C, Postigo-Hidalgo I, Berneck BS, Ulbert S, et al. Rapid decline of Zika virus NS1 antigen-specific antibody responses, northeastern Brazil. Virus Genes. 2020;56: 632–637. doi:10.1007/s11262-020-01772-2

30. Alves LV, Leal CA, Alves JGB. Zika virus seroprevalence in women who gave birth during Zika virus outbreak in Brazil - a prospective observational study. Heliyon. 2020;6: e04817. doi:10.1016/j.heliyon.2020.e04817

31. Diefenbach CF, Slavov SN, Kashima S, Ferreira AR, Hespanhol MR, Bandeira BS, et al. Prevalence of Zika Virus (Zikv) in blood donors from a hemotherapy service of the southern region of Brazil. ISBT Science Series. 2019;14: 157–162. doi:10.1111/voxs.12436

32. Wittlin BB, Almeida DV de, Marques BCL, Monteiro CC, Moreira LF de S, Linhares JHR, et al. Chikungunya, Zika and Dengue seroprevalence rates among pregnant women in a hospital of southeastern Brazil. Soroprevalência de chikungunya, zika e dengue em gestantes de um hospital do sudeste do Brasil. 2021 [cited 6 Apr 2024]. Available: https://www.arca.fiocruz.br/handle/icict/56611

33. Barreto FK de A, Alencar CH, Araújo FM de C, Oliveira R de MAB, Cavalcante JW, Lemos DRQ, et al. Seroprevalence, spatial dispersion and factors associated with flavivirus and chikungunha infection in a risk area: a population-based seroprevalence study in Brazil. BMC Infect Dis. 2020;20: 881. doi:10.1186/s12879-020-05611-5

34. Périssé ARS, Souza-Santos R, Duarte R, Santos F, de Andrade CR, Rodrigues NCP, et al. Zika, dengue and chikungunya population prevalence in Rio de Janeiro city, Brazil, and the importance of seroprevalence studies to estimate the real number of infected individuals. PLoS One. 2020;15: e0243239. doi:10.1371/journal.pone.0243239

35. Braga C, Martelli CMT, Souza WV, Luna CF, Albuquerque M de FPM, Mariz CA, et al. Seroprevalence of Dengue, Chikungunya and Zika at the epicenter of the congenital microcephaly epidemic in Northeast Brazil: A population-based survey. PLOS Neglected Tropical Diseases. 2023;17: e0011270. doi:10.1371/journal.pntd.0011270

36. Francisco MV, Costa B, Almeida B, Santos C, Casaes AC, Santos YD, et al. Seroprevalence of Zika, Chikungunya and Dengue viruses in a rural area of northeastern Brazil. International Journal of Infectious Diseases. 2020;101: 245. doi:10.1016/j.ijid.2020.11.074

37. Araújo TMD, Souza FDO, Helioterio MC, Andrade KVFD, Pinho PDS, Werneck GL. The high prevalence of infectious diseases among health workers indicates the need for improving surveillance. Rev bras saúde ocup. 2023;48: e17. doi:10.1590/2317-6369/23021en2023v48e17

38. Bayona-Pacheco B, Acosta-Reyes J, Navarro E, San-Juan H, Bula J, Baquero H. Seroprevalence of Zika virus among blood donors before the epidemic in Barranquilla, Colombia, 2015-2016. An Acad Bras Cienc. 2019;91: e20180860. doi:10.1590/0001-3765201920180860

39. Marbán-Castro E, Arrieta GJ, Martínez MJ, González R, Bardají A, Menéndez C, et al. High Seroprevalence of Antibodies against Arboviruses among Pregnant Women in Rural Caribbean Colombia in the Context of the Zika Virus Epidemic. Antibodies (Basel). 2020;9: 56. doi:10.3390/antib9040056

40. Rivas E, Ojeda J, Garcia-Rivera EJ, Rivera DM, Arredondo JL, Medina EL, et al. Prospective surveillance of Zika virus at the end of the Americas' outbreak: An unexpected outcome. Frontiers in Tropical Diseases. 2022;3. Available: https://www.frontiersin.org/articles/10.3389/fitd.2022.1027908

41. Cardona-Ospina JA, Trujillo AM, Jiménez-Posada EV, Sepúlveda-Arias JC, Tabares-Villa FA, Altieri-Rivera JS, et al. Susceptibility to endemic Aedes-borne viruses among pregnant women in Risaralda, Colombia. Int J Infect Dis. 2022;122: 832–840. doi:10.1016/j.ijid.2022.07.017

42. Flamand C, Bailly S, Fritzell C, Berthelot L, Vanhomwegen J, Salje H, et al. Impact of Zika Virus Emergence in French Guiana: A Large General Population Seroprevalence Survey. The Journal of Infectious Diseases. 2019;220: 1915–1925. doi:10.1093/infdis/jiz396

43. Lamb MM, Paniagua-Avila A, Zacarias A, Rojop N, Chacon A, Natrajan MS, et al. Repeated Rapid Active Sampling Surveys Demonstrated a Rapidly Changing Zika Seroprevalence among Children in a Rural Dengue-endemic Region in Southwest Guatemala during the Zika Epidemic (2015-2016). Am J Trop Med Hyg. 2022;107: 1099–1106. doi:10.4269/ajtmh.22-0399

44. Anzinger JJ, Mears CD, Ades AE, Francis K, Phillips Y, Leys YE, et al. Antenatal Seroprevalence of Zika and Chikungunya Viruses, Kingston Metropolitan Area, Jamaica, 2017-2019. Emerg Infect Dis. 2022;28: 473–475. doi:10.3201/eid2802.211849

45. Gallian P, Cabié A, Richard P, Paturel L, Charrel RN, Pastorino B, et al. Zika virus in asymptomatic blood donors in Martinique. Blood. 2017;129: 263–266. doi:10.1182/blood-2016-09-737981

46. Eligio-García L, Crisóstomo-Vázquez M del P, Caballero-García M de L, Soria-Guerrero M, Méndez–Galván JF, López-Cancino SA, et al. Co-infection of Dengue, Zika and Chikungunya in a group of pregnant women from Tuxtla Gutiérrez, Chiapas: Preliminary data. 2019. PLOS Neglected Tropical Diseases. 2020;14: e0008880. doi:10.1371/journal.pntd.0008880

47. Collins MH, Zepeda O, Blette B, Jadi R, Morales M, Pérez R, et al. Serologic surveillance of maternal Zika infection in a prospective cohort in Leon, Nicaragua during the peak of the Zika epidemic. PLoS One. 2020;15: e0230692. doi:10.1371/journal.pone.0230692

48. Zambrana JV, Bustos Carrillo F, Burger-Calderon R, Collado D, Sanchez N, Ojeda S, et al. Seroprevalence, risk factor, and spatial analyses of Zika virus infection after the 2016 epidemic in Managua, Nicaragua. Proc Natl Acad Sci U S A. 2018;115: 9294–9299. doi:10.1073/pnas.1804672115

49. Zepeda O, Espinoza DO, Martinez E, Cross KA, Becker-Dreps S, de Silva AM, et al. Antibody Immunity to Zika Virus among Young Children in a Flavivirus-Endemic Area in Nicaragua. Viruses. 2023;15: 796. doi:10.3390/v15030796

50. Cachay R, Schwalb A, Acevedo-Rodriguez JG, Merino X, Talledo M, Suarez-Ognio L, et al. Zika Virus Seroprevalence in Two Districts of Chincha, Ica, Peru: A Cross-Sectional Study. Am J Trop Med Hyg. 2021;106: 192–198. doi:10.4269/ajtmh.20-1339

51. Langerak T, Brinkman T, Mumtaz N, Arron G, Hermelijn S, Baldewsingh G, et al. Zika Virus Seroprevalence in Urban and Rural Areas of Suriname, 2017. J Infect Dis. 2019;220: 28–31. doi:10.1093/infdis/jiz063

52. Ziyaeyan M, Behzadi MA, Leyva-Grado VH, Azizi K, Pouladfar G, Dorzaban H, et al. Widespread circulation of West Nile virus, but not Zika virus in southern Iran. PLoS Negl Trop Dis. 2018;12: e0007022. doi:10.1371/journal.pntd.0007022

53. ShantASinbat AKM. Seroepidemiology of Zikavirus in Basrah, Southern Iraq. Annals of the Romanian Society for Cell Biology. 2021;25: 12546–12553.

54. Alayed MS, Qureshi MA, Ahmed S, Alqahtani AS, Al-Qahtani AM, Alshaybari K, et al. Seroprevalence of Zika virus among asymptomatic pregnant mothers and their newborns in the Najran region of southwest Saudi Arabia. Ann Saudi Med. 2018;38: 408–412. doi:10.5144/0256-4947.2018.408

55. Abushoufa F, Arikan A, Sanlidag T, Guvenir M, Guler E, Suer K. Absence of Zika Virus Seroprevalence Among Blood Donors in Northern Cyprus. J Infect Dev Ctries. 2021;15: 1032–1034. doi:10.3855/jidc.12766

56. Franke F, Noël H, Durand GA, Giron S, Decoppet A, de Valk H, et al. 81 - Enquête de séroprévalence suite à la première transmission vectorielle du Zika en Europe. Revue d'Épidémiologie et de Santé Publique. 2022;70: S167–S168. doi:10.1016/j.respe.2022.06.108

57. Sasmono RT, Johar E, Yohan B, Ma'roef CN, Pronyk P, Hadinegoro SR, et al. Spatiotemporal Heterogeneity of Zika Virus Transmission in Indonesia: Serosurveillance Data from a Pediatric Population. Am J Trop Med Hyg. 2021;104: 2220–2223. doi:10.4269/ajtmh.21-0010

58. Sasmono RT, Dhenni R, Yohan B, Pronyk P, Hadinegoro SR, Soepardi EJ, et al. Zika Virus Seropositivity in 1–4-Year-Old Children, Indonesia, 2014. Emerg Infect Dis. 2018;24: 1740–1743. doi:10.3201/eid2409.180582

59. Harapan H, Panta K, Michie A, Ernst T, McCarthy S, Muhsin M, et al. Hyperendemic Dengue and Possible Zika Circulation in the Westernmost Region of the Indonesian Archipelago. Viruses. 2022;14: 219. doi:10.3390/v14020219

60. Choyrum S, Wangsaeng N, Nechba A, Salvadori N, Saisom R, Achalapong J, et al. Zika Virus Immunoglobulin G Seroprevalence among Young Adults Living with HIV or without HIV in Thailand from 1997 to 2017. Viruses. 2022;14: 368. doi:10.3390/v14020368

61. Yamanaka A, Matsuda M, Okabayashi T, Pitaksajjakul P, Ramasoota P, Saito K, et al. Seroprevalence of Flavivirus Neutralizing Antibodies in Thailand by High-Throughput Neutralization Assay: Endemic Circulation of Zika Virus before 2012. mSphere. 2021;6: e0033921. doi:10.1128/mSphere.00339-21

62. Chakma R, Sriburin P, Sittikul P, Rattanamahaphoom J, Nuprasert W, Thammasonthijarern N, et al. Arbovirus Seroprevalence Study in Bangphae District, Ratchaburi Province, Thailand: Comparison between ELISA and a Multiplex Rapid Diagnostic Test (Chembio DPP® ZCD IgG). Trop Med Infect Dis. 2022;7: 378. doi:10.3390/tropicalmed7110378

63. Sornjai W, Jaratsittisin J, Auewarakul P, Wikan N, Smith DR. Analysis of Zika virus neutralizing antibodies in normal healthy Thais. Sci Rep. 2018;8: 17193. doi:10.1038/s41598-018-35643-6

64. Sirinam S, Chatchen S, Arunsodsai W, Guharat S, Limkittikul K. Seroprevalence of Zika Virus in Amphawa District, Thailand, after the 2016 Pandemic. Viruses. 2022;14: 476. doi:10.3390/v14030476

65. Densathaporn T, Sangthong R, Sakolnapa M, Surasombatpattana S, Kemapunmanus M, Masrinoul P, et al. Survey on neutralizing antibodies against Zika virus eighteen months post-outbreak in two southern Thailand communities. BMC Infect Dis. 2020;20: 921. doi:10.1186/s12879-020-05654-8

66. Phatihattakorn C, Wongsa A, Pongpan K, Anuwuthinawin S, Mungmanthong S, Wongprasert M, et al. Seroprevalence of Zika virus in pregnant women from central Thailand. PLoS One. 2021;16: e0257205. doi:10.1371/journal.pone.0257205

67. Sun J, Su J, Jiao X, Zhou H, Zhang H, Wu D, et al. Community based serosurvey of naïve population indicate no local circulation of Zika virus in an hyper endemic area of China 2016. Journal of Infection. 2019;79: 61–74. doi:10.1016/j.jinf.2019.03.013

68. Li F, Zhou JZ, Zhou L, Fu SH, Tian ZZ, Wang Q, et al. Serological Survey of Zika Virus in Humans and Animals in Dejiang Prefecture, Guizhou Province, China. Biomed Environ Sci. 2019;32: 875–880. doi:10.3967/bes2019.108

69. Zhou C-M, Liu J-W, Qi R, Fang L-Z, Qin X-R, Han H-J, et al. Emergence of Zika virus infection in China. PLoS Negl Trop Dis. 2020;14: e0008300. doi:10.1371/journal.pntd.0008300

70. Henderson AD, Aubry M, Kama M, Vanhomwegen J, Teissier A, Mariteragi-Helle T, et al. Zika seroprevalence declines and neutralizing antibodies wane in adults following outbreaks in French Polynesia and Fiji. Elife. 2020;9: e48460. doi:10.7554/eLife.48460

71. Kama M, Aubry M, Naivalu T, Vanhomwegen J, Mariteragi-Helle T, Teissier A, et al. Sustained Low-Level Transmission of Zika and Chikungunya Viruses after Emergence in the Fiji Islands. Emerg Infect Dis. 2019;25: 1535–1538. doi:10.3201/eid2508.180524

72. Aubry M, Finke J, Teissier A, Roche C, Broult J, Paulous S, et al. Seroprevalence of arboviruses among blood donors in French Polynesia, 2011–2013. International Journal of Infectious Diseases. 2015;41: 11–12. doi:10.1016/j.ijid.2015.10.005

73. Cauchemez S, Besnard M, Bompard P, Dub T, Guillemette-Artur P, Eyrolle-Guignot D, et al. Association between Zika virus and microcephaly in French Polynesia, 2013-15: a retrospective study. Lancet. 2016;387: 2125–2132. doi:10.1016/S0140-6736(16)00651-6

74. Aubry M, Teissier A, Huart M, Merceron S, Vanhomwegen J, Roche C, et al. Zika Virus Seroprevalence, French Polynesia, 2014-2015. Emerg Infect Dis. 2017;23: 669–672. doi:10.3201/eid2304.161549

75. Pastorino B, Sengvilaipaseuth O, Chanthongthip A, Vongsouvath M, Souksakhone C, Mayxay M, et al. Low Zika Virus Seroprevalence in Vientiane, Laos, 2003–2015. Am J Trop Med Hyg. 2019;100: 639–642. doi:10.4269/ajtmh.18-0439

76. Sam I-C, Montoya M, Chua CL, Chan YF, Pastor A, Harris E. Low seroprevalence rates of Zika virus in Kuala Lumpur, Malaysia. Trans R Soc Trop Med Hyg. 2019;113: 678–684. doi:10.1093/trstmh/trz056

77. Khoo H-Y, Lee H-Y, Khor C-S, Tan K-K, Bin Hassan MR, Wong CM, et al. Seroprevalence of Zika Virus among Forest Fringe Communities in Peninsular Malaysia and Sabah: General Population-Based Study. Am J Trop Med Hyg. 2022;107: 560–568. doi:10.4269/ajtmh.21-0988

78. Khor C-S, Mohd-Rahim N-F, Hassan H, Tan K-K, Zainal N, Teoh B-T, et al. Serological evidence of DENV, JEV, and ZIKV among the indigenous people (Orang Asli) of Peninsular Malaysia. J Med Virol. 2020;92: 956–962. doi:10.1002/jmv.25649

79. Grant R, Kizu J, Graham M, McCallum F, McPherson B, Auliff A, et al. Serological evidence of possible high levels of undetected transmission of Zika virus among Papua New Guinea military personnel, 2019. IJID Regions. 2022;4: 131–133. doi:10.1016/j.ijregi.2022.07.006

80. Russell TL, Horwood PF, Harrington H, Apairamo A, Kama NJ, Bobogare A, et al. Seroprevalence of dengue, Zika, chikungunya and Ross River viruses across the Solomon Islands. PLoS Negl Trop Dis. 2022;16: e0009848. doi:10.1371/journal.pntd.0009848

81. Chien Y-W, Ho T-C, Huang P-W, Ko N-Y, Ko W-C, Perng GC. Low seroprevalence of Zika virus infection among adults in Southern Taiwan. BMC Infect Dis. 2019;19: 884. doi:10.1186/s12879-019-4491-4

82. Adams C, Jadi R, Segovia-Chumbez B, Daag J, Ylade M, Medina FA, et al. Novel Assay to Measure Seroprevalence of Zika Virus in the Philippines. Emerg Infect Dis. 2021;27: 3073–3081. doi:10.3201/eid2712.211150

83. Chiu Y-C, Baud D, Fahmi A, Zumkehr B, Vouga M, Pomar L, et al. Correction: Absence of Zika virus among pregnant women in Vietnam in 2008. Trop Dis Travel Med Vaccines. 2023;9: 6. doi:10.1186/s40794-023-00191-z

84. Nguyen CT, Moi ML, Le TQM, Nguyen TTT, Vu TBH, Nguyen HT, et al. Prevalence of Zika virus neutralizing antibodies in healthy adults in Vietnam during and after the Zika virus epidemic season: a longitudinal population-based survey. BMC Infectious Diseases. 2020;20: 332. doi:10.1186/s12879-020-05042-2
