# Supplementary material for: Global seroprevalence of Zika virus in asymptomatic individuals: A systematic review
Source: PLoS Negl Trop Dis. 2024 Apr 17;18(4):e0011842. doi: 10.1371/journal.pntd.0011842 (PMC11057727; doi:10.1371/journal.pntd.0011842)
Supplement: S3 Table — (PDF) [file pntd.0011842.s003.pdf]

Table S3. Risk of bias assessment

First author, year	Q1	Q2	Q3	Q4	Q5	Q6	Q7	Q8	Q9	Q10	Total	Bias
Abushoufa et al., 2021[1]	Not	Not	Not	Yes	Yes	Yes	Not	Yes	Yes	Yes	6	moderate risk
Adams et al., 2021[2]	Not	Yes	Yes	Yes	Yes	Yes	Yes	Yes	Yes	Yes	9	low risk
Alayed et al., 2018[3]	Not	Yes	Not	Yes	Yes	Yes	Not	Yes	Yes	Yes	7	low risk
Alves et al., 2020[4]	Not	Not	Not	Yes	Yes	Yes	Yes	Yes	Yes	Yes	7	low risk
Anejo-Okopi et al., 2020[5]	Not	Not	Not	Yes	Yes	Yes	Yes	Yes	Yes	Yes	7	low risk
Anzinger et al., 2022[6]	Not	Not	Not	Yes	Yes	Yes	Yes	Yes	Yes	Yes	7	low risk
Asebe et al., 2021[7]	Not	Not	Not	Yes	Yes	Yes	Yes	Yes	Yes	Yes	7	low risk
Aubry et al., 2015[8]	Not	Unclear	Unclear	Yes	Yes	Yes	Yes	Yes	Yes	Yes	7	low risk
Aubry et al., 2017[9]	Unclear	Unclear	Yes	Yes	Unclear	Yes	Not	Yes	Yes	Yes	6	moderate risk
Babaniyi et al., 2014[10]	Unclear	Yes	Yes	Unclear	Yes	Unclear	Not	Yes	Unclear	Not	4	moderate risk
Batista Salgado et al., 2023[11]	Not	Yes	Yes	Yes	Yes	Yes	Yes	Yes	Yes	Yes	9	low risk
Bayona-Pacheco et al., 2019[12]	Not	Not	Not	Yes	Yes	Yes	Yes	Yes	Yes	Yes	7	low risk
Braga et al., 2023[13]	Not	Yes	yes	yes	Yes	Yes	Yes	Yes	Yes	Yes	9	low risk
Cachay et al., 2022[14]	Not	Not	Unclear	Yes	Yes	Yes	Yes	Yes	Yes	Yes	7	low risk
Cardona-Ospina et al., 2022[15]	Not	Not	Not	Yes	Yes	Yes	Yes	Yes	Yes	Yes	7	low risk
Cauchemez et al., 2016[16]	Unclear	Unclear	Unclear	Unclear	Unclear	Yes	Yes	Yes	Yes	Yes	5	moderate risk
Chakma et al., 2022[17]	Not	Yes	Yes	Unclear	Yes	Yes	Not	Yes	Yes	Unclear	6	moderate risk
Chepkorir et al., 2019[18]	Not	Unclear	Unclear	Yes	Yes	Yes	Yes	Yes	Yes	Yes	7	low risk
Chien et al., 2019[19]	Not	Not	Not	Yes	Yes	Yes	Yes	Yes	Yes	Yes	7	low risk
Chisenga et al., 2020[20]	Not	Yes	Unclear	Yes	Yes	Yes	Yes	Yes	Yes	Yes	8	low risk
Chiu et al., 2023[21]	Not	Not	Not	Yes	Yes	Yes	Yes	Yes	Yes	Yes	7	low risk
Choyrum et al., 2022[22]	Yes	Yes	Yes	Yes	Unclear	Yes	Not	Unclear	Yes	Yes	7	low risk

Collins et al., 2020[23]	Not	Not	Not	Not	Yes	Yes	Yes	Yes	Yes	Yes	6	moderate risk
de Almeida Barreto et al., 2020[24]	Yes	Yes	Yes	Yes	Yes	Yes	Yes	Yes	Yes	Yes	10	low risk
Densathaporn et al., 2020[25]	Not	Yes	Yes	Unclear	Yes	Yes	Yes	Yes	Yes	Yes	8	low risk
Diarra et al., 2020[26]	Not	Yes	Yes	Yes	Yes	Yes	Yes	Yes	Yes	Yes	9	low risk
Diefenbach et al., 2019[27]	Not	Not	Yes	Yes	Yes	Yes	Yes	Yes	Yes	Yes	8	low risk
Eligio-García et al., 2020[28]	Not	Not	Not	Yes	Yes	Yes	Yes	Yes	Yes	Yes	7	low risk
Flamand et al., 2019[29]	Yes	Yes	Yes	Yes	Yes	Yes	Yes	Yes	Yes	Yes	10	low risk
Francisco et al., 2020[30]	Not	Not	Not	Unclear	Unclear	Unclear	Yes	Yes	Yes	Yes	4	moderate risk
Franke et al., 2020[31]	Not	Yes	Yes	Yes	Yes	Yes	Yes	Yes	Yes	Yes	9	low risk
Gake et al., 2017[32]	Not	Unclear	Unclear	Yes	Yes	Yes	Yes	Yes	Yes	Yes	7	low risk
Gallian et al., 2017[33]	Not	Unclear	Unclear	Yes	Yes	Yes	Yes	Yes	Yes	Yes	7	low risk
Grant et al., 2022[34]	Not	Not	Not	Yes	Yes	Yes	Yes	Yes	Yes	Yes	7	low risk
Harapan et al., 2022[35]	Not	Not	Not	Yes	Yes	Yes	Yes	Yes	Yes	Yes	7	low risk
Henderson et al., 2020[36]	Not	Yes	Yes	Yes	Yes	Yes	Yes	Yes	Yes	Yes	9	low risk
Kama et al., 2019[37]	Not	Yes	Yes	Yes	Yes	Yes	Yes	Yes	Yes	Yes	9	low risk
Khoo et al., 2022[38]	Not	Yes	Yes	Unclear	Yes	Yes	Yes	Yes	Yes	Yes	8	low risk
Khor et al., 2020[39]	Not	Yes	Not	Yes	Yes	Yes	Yes	Yes	Not	Yes	7	low risk
Kisuya et al., 2019[40]	Not	Not	Not	Yes	Unclear	Yes	Yes	Yes	Yes	Yes	6	moderate risk
Kolawole et al., 2020[41]	Not	Not	Not	Yes	Yes	Yes	Yes	Yes	Yes	Yes	7	low risk
Lamb et al., 2022[42]	Not	Yes	Yes	Yes	Yes	Yes	Yes	Yes	Yes	Yes	9	low risk
Langerak et al., 2019[43]	Not	Not	Not	Yes	Yes	Yes	Yes	Yes	Yes	Yes	7	low risk
Li et al., 2019[44]	Not	Not	Not	Yes	Unclear	Yes	Yes	Unclear	Yes	Yes	5	moderate risk
Mac et al., 2023[45]	Not	Yes	Yes	Yes	Yes	Yes	Yes	Yes	Yes	Yes	9	low risk
Marbán Castro et al., 2020[46]	Not	Not	Not	Yes	Yes	Yes	Yes	Yes	Yes	Yes	7	low risk
Marchi et al., 2020[47]	Not	Unclear	Unclear	Yes	Yes	Yes	Yes	Yes	Yes	Yes	7	low risk
Maria de Araújo et al., 2023[48]	Not	Yes	Not	Yes	Yes	Yes	Unclear	Yes	Yes	Yes	7	low risk
Mathé et al., 2018[49]	Not	Not	Not	Unclear	Unclear	Yes	Unclear	Yes	Yes	Yes	4	moderate risk

Moreira-Soto et al., 2020[50]	Not	Not	Not	Yes	Yes	Yes	Yes	Yes	Yes	Yes	7	low risk
Mwanyika et al., 2021[51]	Not	Not	Yes	Yes	Yes	Yes	Yes	Yes	Yes	Yes	8	low risk
Netto et al., 2017[52]	Not	Unclear	Unclear	Yes	Yes	Yes	Yes	Yes	Yes	Yes	7	low risk
Nguyen et al., 2020[53]	Not	Yes	Yes	Yes	Yes	Yes	Yes	Yes	Yes	Yes	9	low risk
Nurtop et al., 2020[54]	Not	Not	Not	Yes	Yes	Yes	Yes	Yes	Yes	Yes	7	low risk
Pastorino et al., 2019[55]	Not	Not	Not	Yes	Yes	Yes	Yes	Yes	Yes	Yes	7	low risk
Phatithattokorn et al., 2021[56]	Not	Yes	Yes	Yes	Yes	Yes	Yes	Yes	Yes	Yes	9	low risk
Rivas et al., 2022[57]	Not	Not	Unclear	Yes	Unclear	Yes	Yes	Unclear	Yes	Yes	5	moderate risk
Russell et al., 2022[58]	Not	Yes	Yes	Yes	Yes	Yes	Yes	Yes	Yes	Yes	9	low risk
Saba Villarroel et al., 2018[59]	Not	Not	Not	Yes	Yes	Yes	Yes	Yes	Yes	Yes	7	low risk
Salgado et al., 2021[60]	Not	Yes	Yes	Yes	Yes	Yes	Not	Yes	Yes	Yes	8	low risk
Sam et al., 2019[61]	Not	Yes	Unclear	Unclear	Yes	Unclear	Yes	Yes	Yes	Yes	6	moderate risk
Santos Périssé et al., 2020[62]	Yes	Yes	Yes	Yes	Yes	Yes	Not	Yes	Yes	Yes	9	low risk
Sasmono et al., 2018[63]	Yes	Yes	Unclear	Unclear	Yes	Yes	Yes	Yes	Yes	Yes	8	low risk
Sasmono et al., 2021[64]	Yes	Yes	Unclear	Unclear	Yes	Yes	Yes	Yes	Yes	Yes	8	low risk
Schwarz et al., 2017[65]	Not	Unclear	Unclear	Yes	Unclear	Yes	Yes	Unclear	Yes	Yes	5	moderate risk
Seruyange et al., 2018[66]	Not	Unclear	Unclear	Yes	Yes	Yes	Yes	Yes	Yes	Yes	7	low risk
Shaibu et al., 2021[67]	Not	Not	Unclear	Not	Yes	Yes	Yes	Yes	Yes	Yes	6	moderate risk
Sherman et al., 2018[68]	Not	Not	Not	Yes	Yes	Yes	Yes	Yes	Yes	Yes	7	low risk
Sinbat et al., 2021[69]	Not	Not	Not	Unclear	Yes	Yes	Not	Unclear	Yes	Yes	4	moderate risk
Sirinam et al., 2022[70]	Not	Unclear	Unclear	Not	Yes	Yes	Yes	Yes	Yes	Yes	6	moderate risk
Slavov et al., 2020[71]	Not	Not	Not	Yes	Yes	Yes	Yes	Yes	Yes	Yes	7	low risk
Soghaier et al., 2018[72]	Not	Yes	Yes	Yes	Yes	Yes	Yes	Yes	Yes	Yes	9	low risk
Sornjai et al., 2018[73]	Not	Not	Yes	Yes	Yes	Yes	Yes	Yes	Yes	Yes	8	low risk
Sun et al., 2019[74]	Not	Not	Not	Yes	Yes	Yes	Yes	Yes	Yes	Yes	7	low risk
Tinto et al., 2022[75]	Not	Not	Not	Yes	Yes	Yes	Yes	Yes	Yes	Yes	7	low risk
Ushijima et al., 2021[76]	Not	Not	Unclear	Yes	Yes	Yes	Yes	Unclear	Yes	Yes	6	moderate risk
Ward et al., 2022[77]	Not	Not	Not	Yes	Yes	Yes	Yes	Yes	Yes	Yes	7	low risk

Willcox et al., 2018[78]	Not	yes	yes	yes	yes	yes	yes	yes	yes	yes	9	low risk
Wittlin et al., 2021[79]	Not	Not	Not	Unclear	Yes	Yes	Yes	Yes	Yes	Yes	6	moderate risk
Yamanaka et al., 2021[80]	Not	Not	Not	Unclear	Yes	Yes	Yes	Yes	Yes	Yes	6	moderate risk
Zambrana et al., 2018[81]	Yes	Yes	Yes	Yes	Yes	Yes	Yes	Yes	Yes	Yes	10	low risk
Zepeda et al., 2023[82]	Not	Not	Not	Yes	Yes	Yes	Yes	Yes	Yes	Yes	7	low risk
Zhou et al., 2020[83]	Not	Not	Not	Yes	Yes	Yes	Yes	Yes	Yes	Yes	7	low risk
Ziyaeyan et al., 2018[84]	Not	Not	Yes	Yes	Yes	Yes	Not	Unclear	Yes	Yes	6	moderate risk

References

1. Abushoufa F, Arikan A, Sanlidag T, Guvenir M, Guler E, Suer K. Absence of Zika Virus Seroprevalence Among Blood Donors in Northern Cyprus. J Infect Dev Ctries. 2021;15: 1032–1034. doi:10.3855/jidc.12766

2. Adams C, Jadi R, Segovia-Chumbez B, Daag J, Ylade M, Medina FA, et al. Novel Assay to Measure Seroprevalence of Zika Virus in the Philippines. Emerg Infect Dis. 2021;27: 3073–3081. doi:10.3201/eid2712.211150

3. Alayed MS, Qureshi MA, Ahmed S, Alqahtani AS, Al-Qahtani AM, Alshaybari K, et al. Seroprevalence of Zika virus among asymptomatic pregnant mothers and their newborns in the Najran region of southwest Saudi Arabia. Ann Saudi Med. 2018;38: 408–412. doi:10.5144/0256-4947.2018.408

4. Alves LV, Leal CA, Alves JGB. Zika virus seroprevalence in women who gave birth during Zika virus outbreak in Brazil - a prospective observational study. Heliyon. 2020;6: e04817. doi:10.1016/j.heliyon.2020.e04817

5. Anejo-Okopi J, Gotom DY, Chiehiura NA, Okojokwu JO, Amanyi DO, Egbere JO, et al. The Seroprevalence of Zika Virus Infection among HIV Positive and HIV Negative Pregnant Women in Jos, Nigeria. Hosts and Viruses. 637134336000000000;7. doi:10.17582/journal.hv/2020/7.6.129.136

6. Anzinger JJ, Mears CD, Ades AE, Francis K, Phillips Y, Leys YE, et al. Antenatal Seroprevalence of Zika and Chikungunya Viruses, Kingston Metropolitan Area, Jamaica, 2017-2019. Emerg Infect Dis. 2022;28: 473–475. doi:10.3201/eid2802.211849

7. Asebe G, Michlmayr D, Mamo G, Abegaz WE, Endale A, Medhin G, et al. Seroprevalence of Yellow fever, Chikungunya, and Zika virus at a community level in the Gambella Region, South West Ethiopia. PLoS ONE. 2021;16. doi:10.1371/journal.pone.0253953

8. Aubry M, Finke J, Teissier A, Roche C, Broult J, Paulous S, et al. Seroprevalence of arboviruses among blood donors in French Polynesia, 2011–2013. International Journal of Infectious Diseases. 2015;41: 11–12. doi:10.1016/j.ijid.2015.10.005

9. Aubry M, Teissier A, Huart M, Merceron S, Vanhomwegen J, Roche C, et al. Zika Virus Seroprevalence, French Polynesia, 2014-2015. Emerg Infect Dis. 2017;23: 669–672. doi:10.3201/eid2304.161549

10. Babaniyi OA, Mwaba P, Songolo P, Mazaba-Liwewe ML, MweeneNdumba I, Masaninga F, et al. Seroprevalence of Zika virus infection specific IgG in Western and North-Western Provinces of Zambia. International Journal of Public Health and Epidemiology. 2014;4: 1–6.

11. Salgado BB, Maués FC de J, Jordão M, Pereira RL, Toledo-Teixeira DA, Parise PL, et al. Antibody cross-reactivity and evidence of susceptibility to emerging Flaviviruses in the dengue-endemic Brazilian Amazon. Int J Infect Dis. 2023;129: 142–151. doi:10.1016/j.ijid.2023.01.033

12. Bayona-Pacheco B, Acosta-Reyes J, Navarro E, San-Juan H, Bula J, Baquero H. Seroprevalence of Zika virus among blood donors before the epidemic in Barranquilla, Colombia, 2015-2016. An Acad Bras Cienc. 2019;91: e20180860. doi:10.1590/0001-3765201920180860

13. Braga C, Martelli CMT, Souza WV, Luna CF, Albuquerque M de FPM, Mariz CA, et al. Seroprevalence of Dengue, Chikungunya and Zika at the epicenter of the congenital microcephaly epidemic in Northeast Brazil: A population-based survey. PLOS Neglected Tropical Diseases. 2023;17: e0011270. doi:10.1371/journal.pntd.0011270

14. Cachay R, Schwalb A, Acevedo-Rodriguez JG, Merino X, Talledo M, Suarez-Ognio L, et al. Zika Virus Seroprevalence in Two Districts of Chincha, Ica, Peru: A Cross-Sectional Study. Am J Trop Med Hyg. 2021;106: 192–198. doi:10.4269/ajtmh.20-1339

15. Cardona-Ospina JA, Trujillo AM, Jiménez-Posada EV, Sepúlveda-Arias JC, Tabares-Villa FA, Altieri-Rivera JS, et al. Susceptibility to endemic Aedes-borne viruses among pregnant women in Risaralda, Colombia. Int J Infect Dis. 2022;122: 832–840. doi:10.1016/j.ijid.2022.07.017

16. Cauchemez S, Besnard M, Bompard P, Dub T, Guillemette-Artur P, Eyrolle-Guignot D, et al. Association between Zika virus and microcephaly in French Polynesia, 2013-15: a retrospective study. Lancet. 2016;387: 2125–2132. doi:10.1016/S0140-6736(16)00651-6

17. Chakma R, Sriburin P, Sittikul P, Rattanamahaphoom J, Nuprasert W, Thammasonthijarern N, et al. Arbovirus Seroprevalence Study in Bangphae District, Ratchaburi Province, Thailand: Comparison between ELISA and a Multiplex Rapid Diagnostic Test (Chembio DPP® ZCD IgG). Trop Med Infect Dis. 2022;7: 378. doi:10.3390/tropicalmed7110378

18. Chepkorir E, Tchouassi DP, Konongoi SL, Lutomiah J, Tigoi C, Irura Z, et al. Serological evidence of Flavivirus circulation in human populations in Northern Kenya: an assessment of disease risk 2016-2017. Virol J. 2019;16: 65. doi:10.1186/s12985-019-1176-y

19. Chien Y-W, Ho T-C, Huang P-W, Ko N-Y, Ko W-C, Perng GC. Low seroprevalence of Zika virus infection among adults in Southern Taiwan. BMC Infect Dis. 2019;19: 884. doi:10.1186/s12879-019-4491-4

20. Chisenga CC, Bosomprah S, Musukuma K, Mubanga C, Chilyabanyama ON, Velu RM, et al. Sero-prevalence of arthropod-borne viral infections among Lukanga swamp residents in Zambia. PLoS One. 2020;15: e0235322. doi:10.1371/journal.pone.0235322

21. Chiu Y-C, Baud D, Fahmi A, Zumkehr B, Vouga M, Pomar L, et al. Absence of Zika virus among pregnant women in Vietnam in 2008. Tropical Diseases, Travel Medicine and Vaccines. 2023;9: 4. doi:10.1186/s40794-023-00189-7

22. Choyrum S, Wangsaeng N, Nechba A, Salvadori N, Saisom R, Achalapong J, et al. Zika Virus Immunoglobulin G Seroprevalence among Young Adults Living with HIV or without HIV in Thailand from 1997 to 2017. Viruses. 2022;14: 368. doi:10.3390/v14020368

23. Collins MH, Zepeda O, Blette B, Jadi R, Morales M, Pérez R, et al. Serologic surveillance of maternal Zika infection in a prospective cohort in Leon, Nicaragua during the peak of the Zika epidemic. PLoS One. 2020;15: e0230692. doi:10.1371/journal.pone.0230692

24. Barreto FK de A, Alencar CH, Araújo FM de C, Oliveira R de MAB, Cavalcante JW, Lemos DRQ, et al. Seroprevalence, spatial dispersion and factors associated with flavivirus and chikungunha infection in a risk area: a population-based seroprevalence study in Brazil. BMC Infect Dis. 2020;20: 881. doi:10.1186/s12879-020-05611-5

25. Densathaporn T, Sangthong R, Sakolnapa M, Surasombatpattana S, Kemapunmanus M, Masrinoul P, et al. Survey on neutralizing antibodies against Zika virus eighteen months post-outbreak in two southern Thailand communities. BMC Infect Dis. 2020;20: 921. doi:10.1186/s12879-020-05654-8

26. Diarra I, Nurtop E, Sangaré AK, Sagara I, Pastorino B, Sacko S, et al. Zika Virus Circulation in Mali. Emerg Infect Dis. 2020;26: 945–952. doi:10.3201/eid2605.191383

27. Diefenbach CF, Slavov SN, Kashima S, Ferreira AR, Hespanhol MR, Bandeira BS, et al. Prevalence of Zika Virus (Zikv) in blood donors from a hemotherapy service of the southern region of Brazil. ISBT Science Series. 2019;14: 157–162. doi:10.1111/voxs.12436

28. Eligio-García L, Crisóstomo-Vázquez M del P, Caballero-García M de L, Soria-Guerrero M, Méndez–Galván JF, López-Cancino SA, et al. Co-infection of Dengue, Zika and Chikungunya in a group of pregnant women from Tuxtla Gutiérrez, Chiapas: Preliminary data. 2019. PLOS Neglected Tropical Diseases. 2020;14: e0008880. doi:10.1371/journal.pntd.0008880

29. Flamand C, Bailly S, Fritzell C, Berthelot L, Vanhomwegen J, Salje H, et al. Impact of Zika Virus Emergence in French Guiana: A Large General Population Seroprevalence Survey. The Journal of Infectious Diseases. 2019;220: 1915–1925. doi:10.1093/infdis/jiz396

30. Francisco MV, Costa B, Almeida B, Santos C, Casaes AC, Santos YD, et al. Seroprevalence of Zika, Chikungunya and Dengue viruses in a rural area of northeastern Brazil. International Journal of Infectious Diseases. 2020;101: 245. doi:10.1016/j.ijid.2020.11.074

31. Franke F, Noël H, Durand GA, Giron S, Decoppet A, de Valk H, et al. 81 - Enquête de séroprévalence suite à la première transmission vectorielle du Zika en Europe. Revue d'Épidémiologie et de Santé Publique. 2022;70: S167–S168. doi:10.1016/j.respe.2022.06.108

32. Gake B, Vernet MA, Leparc-Goffart I, Drexler JF, Gould EA, Gallian P, et al. Low seroprevalence of Zika virus in Cameroonian blood donors. Braz J Infect Dis. 2017;21: 481–483. doi:10.1016/j.bjid.2017.03.018

33. Gallian P, Cabié A, Richard P, Paturel L, Charrel RN, Pastorino B, et al. Zika virus in asymptomatic blood donors in Martinique. Blood. 2017;129: 263–266. doi:10.1182/blood-2016-09-737981

34. Grant R, Kizu J, Graham M, McCallum F, McPherson B, Auliff A, et al. Serological evidence of possible high levels of undetected transmission of Zika virus among Papua New Guinea military personnel, 2019. IJID Regions. 2022;4: 131–133. doi:10.1016/j.ijregi.2022.07.006

35. Harapan H, Panta K, Michie A, Ernst T, McCarthy S, Muhsin M, et al. Hyperendemic Dengue and Possible Zika Circulation in the Westernmost Region of the Indonesian Archipelago. Viruses. 2022;14: 219. doi:10.3390/v14020219

36. Henderson AD, Aubry M, Kama M, Vanhomwegen J, Teissier A, Mariteragi-Helle T, et al. Zika seroprevalence declines and neutralizing antibodies wane in adults following outbreaks in French Polynesia and Fiji. Elife. 2020;9: e48460. doi:10.7554/eLife.48460

37. Kama M, Aubry M, Naivalu T, Vanhomwegen J, Mariteragi-Helle T, Teissier A, et al. Sustained Low-Level Transmission of Zika and Chikungunya Viruses after Emergence in the Fiji Islands. Emerg Infect Dis. 2019;25: 1535–1538. doi:10.3201/eid2508.180524

38. Khoo H-Y, Lee H-Y, Khor C-S, Tan K-K, Bin Hassan MR, Wong CM, et al. Seroprevalence of Zika Virus among Forest Fringe Communities in Peninsular Malaysia and Sabah: General Population-Based Study. Am J Trop Med Hyg. 2022;107: 560–568. doi:10.4269/ajtmh.21-0988

39. Khor C-S, Mohd-Rahim N-F, Hassan H, Tan K-K, Zainal N, Teoh B-T, et al. Serological evidence of DENV, JEV, and ZIKV among the indigenous people (Orang Asli) of Peninsular Malaysia. J Med Virol. 2020;92: 956–962. doi:10.1002/jmv.25649

40. Kisuya B, Masika MM, Bahizire E, Oyugi JO. Seroprevalence of Zika virus in selected regions in Kenya. Transactions of The Royal Society of Tropical Medicine and Hygiene. 2019;113: 735–739. doi:10.1093/trstmh/trz077

41. Kolawole OM, Suleiman MM, Bamidele EP. Molecular epidemiology of Zika virus and Rubella virus in pregnant women attending Sobi Specialist Hospital Ilorin, Nigeria. International Journal of Research in Medical Sciences. 2020;8: 2275–2283. doi:10.18203/2320-6012.ijrms20202234

42. Lamb MM, Paniagua-Avila A, Zacarias A, Rojop N, Chacon A, Natrajan MS, et al. Repeated Rapid Active Sampling Surveys Demonstrated a Rapidly Changing Zika Seroprevalence among Children in a Rural Dengue-endemic Region in Southwest Guatemala during the Zika Epidemic (2015-2016). Am J Trop Med Hyg. 2022;107: 1099–1106. doi:10.4269/ajtmh.22-0399

43. Langerak T, Brinkman T, Mumtaz N, Arron G, Hermelijn S, Baldewsingh G, et al. Zika Virus Seroprevalence in Urban and Rural Areas of Suriname, 2017. J Infect Dis. 2019;220: 28–31. doi:10.1093/infdis/jiz063

44. Li F, Zhou JZ, Zhou L, Fu SH, Tian ZZ, Wang Q, et al. Serological Survey of Zika Virus in Humans and Animals in Dejiang Prefecture, Guizhou Province, China. Biomed Environ Sci. 2019;32: 875–880. doi:10.3967/bes2019.108

45. Mac PA, Kroeger A, Daehne T, Anyaike C, Velayudhan R, Panning M. Zika, Flavivirus and Malaria Antibody Cocirculation in Nigeria. Tropical Medicine and Infectious Disease. 2023;8: 171. doi:10.3390/tropicalmed8030171

46. Marbán-Castro E, Arrieta GJ, Martínez MJ, González R, Bardají A, Menéndez C, et al. High Seroprevalence of Antibodies against Arboviruses among Pregnant Women in Rural Caribbean Colombia in the Context of the Zika Virus Epidemic. Antibodies (Basel). 2020;9: 56. doi:10.3390/antib9040056

47. Marchi S, Viviani S, Montomoli E, Tang Y, Boccuto A, Vicenti I, et al. Zika Virus in West Africa: A Seroepidemiological Study between 2007 and 2012. Viruses. 2020;12: 641. doi:10.3390/v12060641

48. Araújo TMD, Souza FDO, Helioterio MC, Andrade KVFD, Pinho PDS, Werneck GL. The high prevalence of infectious diseases among health workers indicates the need for improving surveillance. Rev bras saúde ocup. 2023;48: e17. doi:10.1590/2317-6369/23021en2023v48e17

49. Mathé P, Egah DZ, Müller JA, Shehu NY, Obishakin ET, Shwe DD, et al. Low Zika virus seroprevalence among pregnant women in North Central Nigeria, 2016. Journal of Clinical Virology. 2018;105: 35–40. doi:10.1016/j.jcv.2018.05.011

50. Moreira-Soto A, de Souza Sampaio G, Pedroso C, Postigo-Hidalgo I, Berneck BS, Ulbert S, et al. Rapid decline of Zika virus NS1 antigen-specific antibody responses, northeastern Brazil. Virus Genes. 2020;56: 632–637. doi:10.1007/s11262-020-01772-2

51. Mwanyika GO, Sindato C, Rugarabamu S, Rumisha SF, Karimuribo ED, Misinzo G, et al. Seroprevalence and associated risk factors of chikungunya, dengue, and Zika in eight districts in Tanzania. Int J Infect Dis. 2021;111: 271–280. doi:10.1016/j.ijid.2021.08.040

52. Netto EM, Moreira-Soto A, Pedroso C, Höser C, Funk S, Kucharski AJ, et al. High Zika Virus Seroprevalence in Salvador, Northeastern Brazil Limits the Potential for Further Outbreaks. mBio. 2017;8: e01390-17. doi:10.1128/mBio.01390-17

53. Nguyen CT, Moi ML, Le TQM, Nguyen TTT, Vu TBH, Nguyen HT, et al. Prevalence of Zika virus neutralizing antibodies in healthy adults in Vietnam during and after the Zika virus epidemic season: a longitudinal population-based survey. BMC Infectious Diseases. 2020;20: 332. doi:10.1186/s12879-020-05042-2

54. Nurtop E, Moyen N, Dzia-Lepfoundzou A, Dimi Y, Ninove L, Drexler JF, et al. A Report of Zika Virus Seroprevalence in Republic of the Congo. Vector Borne Zoonotic Dis. 2020;20: 40–42. doi:10.1089/vbz.2019.2466

55. Pastorino B, Sengvilaipaseuth O, Chanthongthip A, Vongsouvath M, Souksakhone C, Mayxay M, et al. Low Zika Virus Seroprevalence in Vientiane, Laos, 2003–2015. Am J Trop Med Hyg. 2019;100: 639–642. doi:10.4269/ajtmh.18-0439

56. Phatihattakorn C, Wongsa A, Pongpan K, Anuwuthinawin S, Mungmanthong S, Wongprasert M, et al. Seroprevalence of Zika virus in pregnant women from central Thailand. PLoS One. 2021;16: e0257205. doi:10.1371/journal.pone.0257205

57. Rivas E, Ojeda J, Garcia-Rivera EJ, Rivera DM, Arredondo JL, Medina EL, et al. Prospective surveillance of Zika virus at the end of the Americas' outbreak: An unexpected outcome. Frontiers in Tropical Diseases. 2022;3. Available: https://www.frontiersin.org/articles/10.3389/fitd.2022.1027908

58. Russell TL, Horwood PF, Harrington H, Apairamo A, Kama NJ, Bobogare A, et al. Seroprevalence of dengue, Zika, chikungunya and Ross River viruses across the Solomon Islands. PLoS Negl Trop Dis. 2022;16: e0009848. doi:10.1371/journal.pntd.0009848

59. Saba Villarroel PM, Nurtop E, Pastorino B, Roca Y, Drexler JF, Gallian P, et al. Zika virus epidemiology in Bolivia: A seroprevalence study in volunteer blood donors. PLoS Negl Trop Dis. 2018;12: e0006239. doi:10.1371/journal.pntd.0006239

60. Salgado BB, de Jesus Maués FC, Pereira RL, Chiang JO, de Oliveira Freitas MN, Ferreira MS, et al. Prevalence of arbovirus antibodies in young healthy adult population in Brazil. Parasit Vectors. 2021;14: 403. doi:10.1186/s13071-021-04901-4

61. Sam I-C, Montoya M, Chua CL, Chan YF, Pastor A, Harris E. Low seroprevalence rates of Zika virus in Kuala Lumpur, Malaysia. Trans R Soc Trop Med Hyg. 2019;113: 678–684. doi:10.1093/trstmh/trz056

62. Périssé ARS, Souza-Santos R, Duarte R, Santos F, de Andrade CR, Rodrigues NCP, et al. Zika, dengue and chikungunya population prevalence in Rio de Janeiro city, Brazil, and the importance of seroprevalence studies to estimate the real number of infected individuals. PLoS One. 2020;15: e0243239. doi:10.1371/journal.pone.0243239

63. Sasmono RT, Dhenni R, Yohan B, Pronyk P, Hadinegoro SR, Soepardi EJ, et al. Zika Virus Seropositivity in 1–4-Year-Old Children, Indonesia, 2014. Emerg Infect Dis. 2018;24: 1740–1743. doi:10.3201/eid2409.180582

64. Sasmono RT, Johar E, Yohan B, Ma'roef CN, Pronyk P, Hadinegoro SR, et al. Spatiotemporal Heterogeneity of Zika Virus Transmission in Indonesia: Serosurveillance Data from a Pediatric Population. Am J Trop Med Hyg. 2021;104: 2220–2223. doi:10.4269/ajtmh.21-0010

65. Schwarz NG, Mertens E, Winter D, Maiga-Ascofaré O, Dekker D, Jansen S, et al. No serological evidence for Zika virus infection and low specificity for anti-Zika virus ELISA in malaria positive individuals among pregnant women from Madagascar in 2010. PLoS One. 2017;12: e0176708. doi:10.1371/journal.pone.0176708

66. Seruyange E, Gahutu J-B, Muvunyi CM, Katare S, Ndahindwa V, Sibomana H, et al. Seroprevalence of Zika virus and Rubella virus IgG among blood donors in Rwanda and in Sweden. J Med Virol. 2018;90: 1290–1296. doi:10.1002/jmv.25198

67. Shaibu JO, Okwuraiwe AP, Jakkari A, Dennis A, Akinyemi KO, Li J, et al. Sero-molecular Prevalence of Zika Virus among Pregnant Women Attending Some Public Hospitals in Lagos State, Nigeria. European Journal of Medical and Health Sciences. 2021;3: 77–82. doi:10.24018/ejmed.2021.3.5.1075

68. Sherman KE, Rouster SD, Kong LX, Shata TM, Archampong T, Kwara A, et al. Zika Virus Exposure in an HIV-Infected Cohort in Ghana. J Acquir Immune Defic Syndr. 2018;78: e35–e38. doi:10.1097/QAI.0000000000001718

69. ShantASinbat AKM. Seroepidemiology of Zikavirus in Basrah, Southern Iraq. Annals of the Romanian Society for Cell Biology. 2021;25: 12546–12553.

70. Sirinam S, Chatchen S, Arunsodsai W, Guharat S, Limkittikul K. Seroprevalence of Zika Virus in Amphawa District, Thailand, after the 2016 Pandemic. Viruses. 2022;14: 476. doi:10.3390/v14030476

71. Slavov SN, Guaragna Machado RR, Ferreira AR, Soares CP, Araujo DB, Leal Oliveira DB, et al. Zika virus seroprevalence in blood donors from the Northeastern region of São Paulo State, Brazil, between 2015 and 2017. Journal of Infection. 2020;80: 111–115. doi:10.1016/j.jinf.2019.10.002

72. Soghaier MA, Abdelgadir DM, Abdelkhalig SM, Kafi H, Zarroug IMA, Sall AA, et al. Evidence of pre-existing active Zika virus circulation in Sudan prior to 2012. BMC Res Notes. 2018;11: 906. doi:10.1186/s13104-018-4027-9

73. Sornjai W, Jaratsittisin J, Auewarakul P, Wikan N, Smith DR. Analysis of Zika virus neutralizing antibodies in normal healthy Thais. Sci Rep. 2018;8: 17193. doi:10.1038/s41598-018-35643-6

74. Sun J, Su J, Jiao X, Zhou H, Zhang H, Wu D, et al. Community based serosurvey of naïve population indicate no local circulation of Zika virus in an hyper endemic area of China 2016. Journal of Infection. 2019;79: 61–74. doi:10.1016/j.jinf.2019.03.013

75. Tinto B, Kaboré DPA, Kania D, Kagoné TS, Kiba-Koumaré A, Pinceloup L, et al. Serological Evidence of Zika Virus Circulation in Burkina Faso. Pathogens. 2022;11: 741. doi:10.3390/pathogens11070741

76. Ushijima Y, Abe H, Nguema Ondo G, Bikangui R, Massinga Loembé M, Zadeh VR, et al. Surveillance of the major pathogenic arboviruses of public health concern in Gabon, Central Africa: increased risk of West Nile virus and dengue virus infections. BMC Infect Dis. 2021;21: 265. doi:10.1186/s12879-021-05960-9

77. Ward D, Gomes AR, Tetteh KKA, Sepúlveda N, Gomez LF, Campino S, et al. Sero-epidemiological study of arbovirus infection following the 2015–2016 Zika virus outbreak in Cabo Verde. Sci Rep. 2022;12: 11719. doi:10.1038/s41598-022-16115-4

78. Willcox AC, Collins MH, Jadi R, Keeler C, Parr JB, Mumba D, et al. Seroepidemiology of Dengue, Zika, and Yellow Fever Viruses among Children in the Democratic Republic of the Congo. Am J Trop Med Hyg. 2018;99: 756–763. doi:10.4269/ajtmh.18-0156

79. Wittlin BB, Almeida DV de, Marques BCL, Monteiro CC, Moreira LF de S, Linhares JHR, et al. Chikungunya, Zika and Dengue seroprevalence rates among pregnant women in a hospital of southeastern Brazil. Soroprevalência de chikungunya, zika e dengue em gestantes de um hospital do sudeste do Brasil. 2021 [cited 6 Apr 2024]. Available: https://www.arca.fiocruz.br/handle/icict/56611

80. Yamanaka A, Matsuda M, Okabayashi T, Pitaksajjakul P, Ramasoota P, Saito K, et al. Seroprevalence of Flavivirus Neutralizing Antibodies in Thailand by High-Throughput Neutralization Assay: Endemic Circulation of Zika Virus before 2012. mSphere. 2021;6: e0033921. doi:10.1128/mSphere.00339-21

81. Zambrana JV, Bustos Carrillo F, Burger-Calderon R, Collado D, Sanchez N, Ojeda S, et al. Seroprevalence, risk factor, and spatial analyses of Zika virus infection after the 2016 epidemic in Managua, Nicaragua. Proc Natl Acad Sci U S A. 2018;115: 9294–9299. doi:10.1073/pnas.1804672115

82. Zepeda O, Espinoza DO, Martinez E, Cross KA, Becker-Dreps S, de Silva AM, et al. Antibody Immunity to Zika Virus among Young Children in a Flavivirus-Endemic Area in Nicaragua. Viruses. 2023;15: 796. doi:10.3390/v15030796

83. Zhou C-M, Liu J-W, Qi R, Fang L-Z, Qin X-R, Han H-J, et al. Emergence of Zika virus infection in China. PLoS Negl Trop Dis. 2020;14: e0008300. doi:10.1371/journal.pntd.0008300

84. Ziyaeyan M, Behzadi MA, Leyva-Grado VH, Azizi K, Pouladfar G, Dorzaban H, et al. Widespread circulation of West Nile virus, but not Zika virus in southern Iran. PLoS Negl Trop Dis. 2018;12: e0007022. doi:10.1371/journal.pntd.0007022
