# Supplementary material for: Global seroprevalence of Zika virus in asymptomatic individuals: A systematic review
Source: PLoS Negl Trop Dis. 2024 Apr 17;18(4):e0011842. doi: 10.1371/journal.pntd.0011842 (PMC11057727; doi:10.1371/journal.pntd.0011842)
Supplement: S4 Table — (PDF) [file pntd.0011842.s004.pdf]

S4 Table: Heterogeneity assessment of seroprevalence of Zika virus per World Health Organization region and per study period.

WHO region	Jan 2000-Jul 2023	Sampling Jan 2000-Apr 2015	Sampling May 2015-Jul 2023
	Heterogeneity p, I^2 %, model	Heterogeneity p, I^2 %, model	Heterogeneity p, I^2 %, model
**Worldwide**	**<0.0001, 99.6, R**	**<0.0001, 99.2, R**	**<0.0001, 99.4, R**
**Africa**	**<0.0001, 99.0, R**	**<0.0001, 98.6, R**	**<0.0001, 98.6, R**
Burkina Faso	NA	NA	NA
Cabo Verde	NA	NA	NA
Cameroon	NA	NA	NA
Democratic Republic of the Congo	NA	NA	NA
Ethiopia	NA	NA	NA
Gabon	0.015, 70.1, R	NA	0.02, 74.5, R
Ghana	NA	NA	NA
Kenya	<0.0001, 96.8, R	NA	NA
Madagascar	NA	NA	NA
Mali	<0.0001, 97.1, R	<0.0001, 97.0, R	NA
Nigeria	<0.0001, 95.3, R	NA	<0.0001, 95.3, R
Republic of the Congo	NA	NA	NA
Rwanda	NA	NA	NA
Senegal	0.02, 80.4, R	0.02, 80.4, R	NA
Sudan	NA	NA	NA
Tanzania	NA	NA	NA
The Gambia	0.01, 84.7, R	0.01, 84.7, R	NA
Zambia	0.01, 84.0, R	NA	NA
**America**	**<0.0001, 99.6, R**	**NA**	**<0.0001, 99.6, R**
Bolivia	NA	NA	NA
Brazil	<0.0001, 99.2, R	NA	<0.0001, 99.2, R
Colombia	<0.0001, 99.5, R	NA	<0.0001, 99.5, R
French Guiana	NA	NA	NA
Guatemala	<0.0001, 97.6, R	NA	<0.0001, 97.6, R
Honduras	NA	NA	NA
Jamaica	NA	NA	NA
Martinique	NA	NA	NA
Mexico	0.54, 0, F	NA	0.54, 0, F

Nicaragua	<0.0001, 97.8, R	NA	<0.0001, 97.8, R
Peru	NA	NA	NA
Puerto Rico	NA	NA	NA
Suriname	NA	NA	NA
**Eastern Mediterranean**	**0.09, 65.3, R**	**NA**	**0.09, 65.3, R**
Iran	NA	NA	NA
Iraq	NA	NA	NA
Saudi Arabia	NA	NA	NA
**Europe**	**NA**	**NA**	**NA**
Cyprus	NA	NA	NA
France	NA	NA	NA
Sweden	NA	NA	NA
**Southeast Asia**	**<0.0001, 97.4, R**	**<0.0001, 98.0, R**	**<0.0001, 92.6, R**
Indonesia	0.94, 0, F	0.94, 0, F	NA
Thailand	<0.0001, 96.2, R	<0.0001, 95.9, R	<0.0001, 92.6, R
**Western Pacific**	**<0.0001, 99.4, R**	**<0.0001, 99.5, R**	**<0.0001, 99.3, R**
China	<0.0001, 97.0, R	NA	<0.0001, 97.0, R
Fiji	<0.0001, 93.6, R	0.53, 0, F	0.004, 82.9, R
French Polynesia	<0.0001, 99.5, R	<0.0001, 99.6, R	<0.0001, 99.5, R
Lao PDR	0.0012, 90.4, R	NA	NA
Malaysia	<0.000, 98.2, R	NA	<0.0001, 98.8, R
Papua New Guinea	NA	NA	NA
Solomon Islands	NA	NA	NA
Taiwan	NA	NA	NA
The Philippines	NA	NA	NA
Vietnam	0.66, 0, F	NA	NA

Abbreviations: F: Fixed-effect model; NA: Not applicable; R: Random-effect model
