# Supplementary material for: Global seroprevalence of Zika virus in asymptomatic individuals: A systematic review
Source: PLoS Negl Trop Dis. 2024 Apr 17;18(4):e0011842. doi: 10.1371/journal.pntd.0011842 (PMC11057727; doi:10.1371/journal.pntd.0011842)
Supplement: S5 Table — (PDF) [file pntd.0011842.s005.pdf]

S5 Table. Heterogeneity assessment of subgroup analysis of Zika virus seroprevalence per World Health Organization region.

Characteristics	Worldwide	Africa	America	Eastern Mediterranean	Southeast Asia	Western Pacific
	Heterogeneity<br>p, I^2 %, model	Heterogeneity<br>p, I^2 %, model	Heterogeneity<br>p, I^2 %, model	Heterogeneity<br>p, I^2 %, model	Heterogeneity<br>p, I^2 %, model	Heterogeneity<br>p, I^2 %, model
**Population type**						
General	<0.0001, 99.5, R	<0.0001, 99.2, R	<0.0001, 99.4, R	NA	<0.0001, 97.9, R	<0.0001, 99.2, R
Pregnant women	<0.0001, 99.0, R	<0.0001, 99.3, R	<0.0001, 98.2, R	NA	<0.0001, 75.4, R	0.58, 0, F
Blood donors	<0.0001, 98.8, R	<0.0001, 99.3, R	<0.0001, 98.0, R	NA	NA	<0.0001, 93.1, R
**Age**						
Children	<0.0001, 99.2, R	<0.0001, 98.8, R	<0.0001, 93.8, R	NA	0.8, 0.0, F	0.18, 44.9, F
Adult	<0.0001, 99.5, R	<0.0001, 98.5, R, Bias 0.01	<0.0001, 99.2, R	NA	0.0011, 81.4, R	<0.0001, 98.6, R
Adults & children	<0.0001, 99.6, R	0.4, 0.0, F Bias <0.0001	<0.0001, 98.7, R, Bias <0.0001	<0.0001, 97.5, R; Bias p<0.0001	<0.0001, 99.5, R	<0.0001, 99.6, R
**Settings**						
Urban	<0.0001, 99.0, R	<0.0001, 95.6, R	<0.0001, 99.5, R	0.109, 61.1, F	<0.0001, 98.8, R	<0.0001, 96.0, R
Rural	<0.0001, 99.3, R	<0.0001, 95.3, R	<0.0001, 99.5, R	NA	NA	<0.0001, 98.4, R
Semi-urban	<0.0001, 99.3, R	0.015, 70.1, R	NA	NA	NA	0.71,0, F
**Serological methods**						
ELISA	<0.0001, 99.3, R	<0.0001, 98.6, R	<0.0001, 99.4, R	0.109, 61.1, F	<0.0001, 96.8, R	<0.0001, 99.6, R
ELISA & PRNT or FRNT	<0.0001, 99.7, R	0.662, 0, F	<0.0001, 99.6, R	NA	NA	<0.0001, 98.9, R
ELISA & VNT	<0.0001, 99.2, R	<0.0001, 96.8, R	<0.0001, 99.0, R	NA	NA	<0.0001, 91.8, R
PRNT90	<0.0001, 98.4, R	1, 0, F	NA	NA	<0.0001, 97.7, R	NA
MIA	<0.0001, 98.7, R	NA	NA	NA	NA	<0.0001, 98.7, R
Other methods	<0.0001, 99.5 R	<0.0001, 94.4, R	<0.0001, 98.6, R	NA	NA	<0.0001, 99.5, R
**ZIKV strain**						

Asian	<0.0001, 99.5 R	<0.0001, 97.8, R	<0.0001, 99.2, R	NA	<0.0001, 92.0, R	<0.0001, 99.2, R
African	<0000.1, 99.9 R	<0.0001, 98.2, R	NA	NA	NA	<0.0001, 99.8, R

Abbreviations: ELISA: Enzyme-linked immunosorbent assay; FRNT: Focus reduction neutralization assay; F: Fixed-effect model; MIA: Microsphere immunoassay; NA: No data available; PRNT: Plaque reduction neutralization test; R: Random-effect model; VNT: Virus neutralization test
