# Supplementary material for: Global seroprevalence of Zika virus in asymptomatic individuals: A systematic review
Source: PLoS Negl Trop Dis. 2024 Apr 17;18(4):e0011842. doi: 10.1371/journal.pntd.0011842 (PMC11057727; doi:10.1371/journal.pntd.0011842)
Supplement: S1 Fig — (PDF) [file pntd.0011842.s006.pdf]

Study	Events	Total	Weight	F-T, Random, 95% CI
Jiufeng et al., 2019	1	665	1.29%	0.002 [0.000, 0.006]
Zhou et al., 2020	17	273	1.28%	0.062 [0.036, 0.094]
Sasmono et al., 2021	80	870	1.29%	0.092 [0.074, 0.112]
Sasmono et al., 2018	60	662	1.29%	0.091 [0.070, 0.114]
Sinbat et al., 2021	13	108	1.26%	0.120 [0.065, 0.189]
Pastorino et al. 2019	84	1046	1.29%	0.080 [0.065, 0.098]
Sam et al., 2019	4	359	1.28%	0.011 [0.002, 0.025]
Khoo et al., 2022	148	706	1.29%	0.210 [0.180, 0.240]
Khor et al., 2019	115	872	1.29%	0.132 [0.110, 0.155]
Alayed et al., 2018	76	410	1.28%	0.185 [0.149, 0.225]
Chien et al., 2019	1	212	1.27%	0.005 [0.000, 0.020]
Choyrum et al., 2022	380	1648	1.29%	0.231 [0.211, 0.251]
Yamanaka et al. 2021	25	147	1.27%	0.170 [0.113, 0.235]
Chakma et al., 2022	232	400	1.28%	0.580 [0.531, 0.628]
Sornjai et al., 2018	30	135	1.26%	0.222 [0.156, 0.297]
Sirinam et al., 2022	53	350	1.28%	0.151 [0.116, 0.191]
Densathaporn et al., 2020	418	1567	1.29%	0.267 [0.245, 0.289]
Phatihattakorn et al. 2021	200	650	1.29%	0.308 [0.273, 0.344]
Adams et al., 2021	98	547	1.29%	0.179 [0.148, 0.212]
Chiu et al., 2023	1	176	1.27%	0.006 [0.000, 0.024]
Nguyen et al., 2020	9	801	1.29%	0.011 [0.005, 0.020]
Tinto et al., 2022	114	501	1.29%	0.228 [0.192, 0.265]
Ward et al., 2022	47	431	1.28%	0.109 [0.081, 0.140]
Gake et al., 2017	53	1084	1.29%	0.049 [0.037, 0.063]
Willcox et al., 2018	1	978	1.29%	0.001 [0.000, 0.004]
Asebe et al., 2021	41	150	1.27%	0.273 [0.205, 0.348]
Ushijima et al., 2021	156	387	1.28%	0.403 [0.355, 0.452]
Sherman et al., 2018	31	236	1.28%	0.131 [0.091, 0.178]
Kisuya et al., 2019	1	577	1.29%	0.002 [0.000, 0.007]
Chepkorir et al., 2019	34	877	1.29%	0.039 [0.027, 0.053]
Marchi et al., 2020	97	1521	1.29%	0.064 [0.052, 0.077]
Diarra et al., 2020	142	1430	1.29%	0.099 [0.084, 0.115]
Mathé et al., 2018	48	468	1.28%	0.103 [0.077, 0.132]
Kolawole et al., 2020	46	200	1.27%	0.230 [0.174, 0.291]
Shaibu et al., 2021	12	352	1.28%	0.034 [0.017, 0.056]
Anejo-Okopi et al., 2020	13	90	1.25%	0.144 [0.078, 0.225]
Mac et al., 2023	167	871	1.29%	0.192 [0.166, 0.219]
Nurtop et al., 2019	7	386	1.28%	0.018 [0.007, 0.034]
Seruyange et al., 2018	12	1089	1.29%	0.011 [0.006, 0.018]
Soghaier et al., 2018	1	1722	1.29%	0.001 [0.000, 0.002]
Mwanyika et al., 2021	121	1818	1.29%	0.067 [0.056, 0.078]
Chisenga et al., 2020	23	214	1.27%	0.107 [0.069, 0.153]
Babaniyi et al., 2015	217	3579	1.29%	0.061 [0.053, 0.069]
Saba Villarroel et al., 2018	237	814	1.29%	0.291 [0.260, 0.323]
Slavov et al., 2020	48	730	1.29%	0.066 [0.049, 0.085]
Salgado et al., 2020	92	298	1.28%	0.309 [0.257, 0.362]
Batista Salgado et al., 2023	235	447	1.28%	0.526 [0.479, 0.572]
Netto et al., 2017	401	633	1.29%	0.633 [0.596, 0.671]
Moreira-Soto et al., 2020	141	288	1.28%	0.490 [0.432, 0.547]
Alves et al., 2020	81	132	1.26%	0.614 [0.529, 0.695]
Diefenbach et al., 2018	1	182	1.27%	0.005 [0.000, 0.023]
Wittlin et al., 2021	169	349	1.28%	0.484 [0.432, 0.537]
de Almeida Barreto et al., 2020	225	404	1.28%	0.557 [0.508, 0.605]
Santos Périssé et al., 2020	1080	2120	1.29%	0.509 [0.488, 0.531]
Braga et al., 2023	1043	2070	1.29%	0.504 [0.482, 0.525]
Francisco et al., 2020	71	336	1.28%	0.211 [0.169, 0.257]
Maria de Araújo et al., 2023	177	424	1.28%	0.417 [0.371, 0.465]
Bayona-Pacheco et al., 2019	26	390	1.28%	0.067 [0.044, 0.094]
Marbán Castro et al., 2020	80	90	1.25%	0.889 [0.815, 0.947]
Rivas et al., 2022	1301	2400	1.29%	0.542 [0.522, 0.562]
Cardona-Ospina et al., 2022	99	115	1.26%	0.861 [0.791, 0.919]
Flamand et al., 2019	628	2697	1.29%	0.233 [0.217, 0.249]
Lamb et al., 2022	89	382	1.28%	0.233 [0.192, 0.277]
Anzinger et al., 2022	91	584	1.29%	0.156 [0.127, 0.186]
Gallian et al., 2017	130	594	1.29%	0.219 [0.186, 0.253]
Eligio-García et al., 2020	85	136	1.26%	0.625 [0.542, 0.705]
Collins et al., 2020	110	187	1.27%	0.588 [0.517, 0.658]
Zambrana et al., 2018	2338	5887	1.29%	0.397 [0.385, 0.410]
Zepeda et al., 2023	171	236	1.28%	0.725 [0.666, 0.780]
Cachay et al., 2022	52	400	1.28%	0.130 [0.099, 0.165]
Langerak et al., 2019	270	770	1.29%	0.351 [0.317, 0.385]
Aubry et al., 2015	5	593	1.29%	0.008 [0.002, 0.018]
Henderson et al., 2020	701	1549	1.29%	0.453 [0.428, 0.477]
Cauchemez et al., 2016	411	672	1.29%	0.612 [0.574, 0.648]
Aubry et al., 2017	154	700	1.29%	0.220 [0.190, 0.251]
Kama et al., 2019	130	1089	1.29%	0.119 [0.101, 0.139]
Grant et al., 2022	135	208	1.27%	0.649 [0.583, 0.713]
Russell et al., 2022	287	1021	1.29%	0.281 [0.254, 0.309]
**Total (95% CI)**	**14723**	**61492**		0.210 [0.161, 0.264]

Heterogeneity: $\tau^2=0.08$, $\chi^2=15839.56$, df=77 (P< 0.0001) $I^2=99.6$

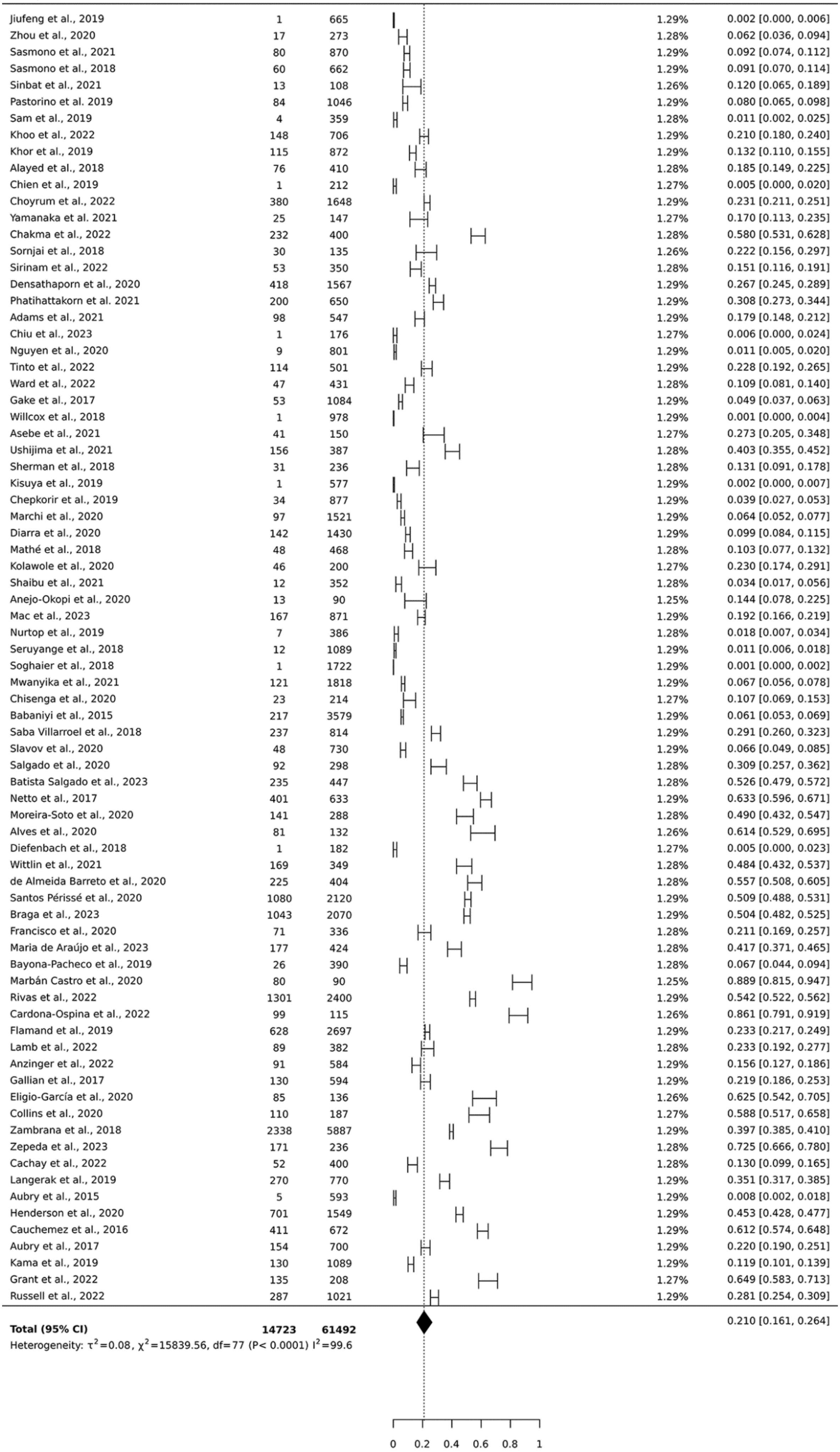

References

1. Jiunfeng Sun J, Su J, Jiao X, Zhou H, Zhang H, Wu D, et al. Community based serosurvey of naïve population indicate no local circulation of Zika virus in an hyper endemic area of China 2016. Journal of Infection. 2019;79: 61–74. doi:10.1016/j.jinf.2019.03.013

2. Zhou C-M, Liu J-W, Qi R, Fang L-Z, Qin X-R, Han H-J, et al. Emergence of Zika virus infection in China. PLoS Negl Trop Dis. 2020;14: e0008300. doi:10.1371/journal.pntd.0008300

3. Sasmono RT, Johar E, Yohan B, Ma'roef CN, Pronyk P, Hadinegoro SR, et al. Spatiotemporal Heterogeneity of Zika Virus Transmission in Indonesia: Serosurveillance Data from a Pediatric Population. Am J Trop Med Hyg. 2021;104: 2220–2223. doi:10.4269/ajtmh.21-0010

4. Sasmono RT, Dhenni R, Yohan B, Pronyk P, Hadinegoro SR, Soepardi EJ, et al. Zika Virus Seropositivity in 1–4-Year-Old Children, Indonesia, 2014. Emerg Infect Dis. 2018;24: 1740–1743. doi:10.3201/eid2409.180582

5. ShantASinbat AKM. Seroepidemiology of Zikavirus in Basrah, Southern Iraq. Annals of the Romanian Society for Cell Biology. 2021;25: 12546–12553.

6. Pastorino B, Sengvilaipaseuth O, Chanthongthip A, Vongsouvath M, Souksakhone C, Mayxay M, et al. Low Zika Virus Seroprevalence in Vientiane, Laos, 2003–2015. Am J Trop Med Hyg. 2019;100: 639–642. doi:10.4269/ajtmh.18-0439

7. Sam I-C, Montoya M, Chua CL, Chan YF, Pastor A, Harris E. Low seroprevalence rates of Zika virus in Kuala Lumpur, Malaysia. Trans R Soc Trop Med Hyg. 2019;113: 678–684. doi:10.1093/trstmh/trz056

8. Khoo H-Y, Lee H-Y, Khor C-S, Tan K-K, Bin Hassan MR, Wong CM, et al. Seroprevalence of Zika Virus among Forest Fringe Communities in Peninsular Malaysia and Sabah: General Population-Based Study. Am J Trop Med Hyg. 2022;107: 560–568. doi:10.4269/ajtmh.21-0988

9. Khor C-S, Mohd-Rahim N-F, Hassan H, Tan K-K, Zainal N, Teoh B-T, et al. Serological evidence of DENV, JEV, and ZIKV among the indigenous people (Orang Asli) of Peninsular Malaysia. J Med Virol. 2020;92: 956–962. doi:10.1002/jmv.25649

10. Alayed MS, Qureshi MA, Ahmed S, Alqahtani AS, Al-Qahtani AM, Alshaybari K, et al. Seroprevalence of Zika virus among asymptomatic pregnant mothers and their newborns in the Najran region of southwest Saudi Arabia. Ann Saudi Med. 2018;38: 408–412. doi:10.5144/0256-4947.2018.408
11. Chien Y-W, Ho T-C, Huang P-W, Ko N-Y, Ko W-C, Perng GC. Low seroprevalence of Zika virus infection among adults in Southern Taiwan. BMC Infect Dis. 2019;19: 884. doi:10.1186/s12879-019-4491-4

12. Choyrum S, Wangsaeng N, Nechba A, Salvadori N, Saisom R, Achalapong J, et al. Zika Virus Immunoglobulin G Seroprevalence among Young Adults Living with HIV or without HIV in Thailand from 1997 to 2017. Viruses. 2022;14: 368. doi:10.3390/v14020368

13. Yamanaka A, Matsuda M, Okabayashi T, Pitaksajjakul P, Ramasoota P, Saito K, et al. Seroprevalence of Flavivirus Neutralizing Antibodies in Thailand by High-Throughput

Neutralization Assay: Endemic Circulation of Zika Virus before 2012. mSphere. 2021;6: e0033921. doi:10.1128/mSphere.00339-21

14. Chakma R, Sriburin P, Sittikul P, Rattanamahaphoom J, Nuprasert W, Thammasonthijarern N, et al. Arbovirus Seroprevalence Study in Bangphae District, Ratchaburi Province, Thailand: Comparison between ELISA and a Multiplex Rapid Diagnostic Test (Chembio DPP® ZCD IgG). Trop Med Infect Dis. 2022;7: 378. doi:10.3390/tropicalmed7110378

15. Sornjai W, Jaratsittisin J, Auewarakul P, Wikan N, Smith DR. Analysis of Zika virus neutralizing antibodies in normal healthy Thais. Sci Rep. 2018;8: 17193. doi:10.1038/s41598-018-35643-6

16. Sirinam S, Chatchen S, Arunsodsai W, Guharat S, Limkittikul K. Seroprevalence of Zika Virus in Amphawa District, Thailand, after the 2016 Pandemic. Viruses. 2022;14: 476. doi:10.3390/v14030476

17. Densathaporn T, Sangthong R, Sakolnapa M, Surasombatpattana S, Kemapunmanus M, Masrinoul P, et al. Survey on neutralizing antibodies against Zika virus eighteen months post-outbreak in two southern Thailand communities. BMC Infect Dis. 2020;20: 921. doi:10.1186/s12879-020-05654-8

18. Phatihattakorn C, Wongsa A, Pongpan K, Anuwuthinawin S, Mungmanthong S, Wongprasert M, et al. Seroprevalence of Zika virus in pregnant women from central Thailand. PLoS One. 2021;16: e0257205. doi:10.1371/journal.pone.0257205

19. Adams C, Jadi R, Segovia-Chumbez B, Daag J, Ylade M, Medina FA, et al. Novel Assay to Measure Seroprevalence of Zika Virus in the Philippines. Emerg Infect Dis. 2021;27: 3073–3081. doi:10.3201/eid2712.211150

20. Chiu Y-C, Baud D, Fahmi A, Zumkehr B, Vouga M, Pomar L, et al. Absence of Zika virus among pregnant women in Vietnam in 2008. Tropical Diseases, Travel Medicine and Vaccines. 2023;9: 4. doi:10.1186/s40794-023-00189-7

21. Nguyen CT, Moi ML, Le TQM, Nguyen TTT, Vu TBH, Nguyen HT, et al. Prevalence of Zika virus neutralizing antibodies in healthy adults in Vietnam during and after the Zika virus epidemic season: a longitudinal population-based survey. BMC Infectious Diseases. 2020;20: 332. doi:10.1186/s12879-020-05042-2

22. Tinto B, Kaboré DPA, Kania D, Kagoné TS, Kiba-Koumaré A, Pinceloup L, et al. Serological Evidence of Zika Virus Circulation in Burkina Faso. Pathogens. 2022;11: 741. doi:10.3390/pathogens11070741

23. Ward D, Gomes AR, Tetteh KKA, Sepúlveda N, Gomez LF, Campino S, et al. Sero-epidemiological study of arbovirus infection following the 2015–2016 Zika virus outbreak in Cabo Verde. Sci Rep. 2022;12: 11719. doi:10.1038/s41598-022-16115-4

24. Gake B, Vernet MA, Leparc-Goffart I, Drexler JF, Gould EA, Gallian P, et al. Low seroprevalence of Zika virus in Cameroonian blood donors. Braz J Infect Dis. 2017;21: 481–483. doi:10.1016/j.bjid.2017.03.018

25. Willcox AC, Collins MH, Jadi R, Keeler C, Parr JB, Mumba D, et al. Seroepidemiology of Dengue, Zika, and Yellow Fever Viruses among Children in the Democratic Republic of the Congo. Am J Trop Med Hyg. 2018;99: 756–763. doi:10.4269/ajtmh.18-0156

26. Asebe G, Michlmayr D, Mamo G, Abegaz WE, Endale A, Medhin G, et al. Seroprevalence of Yellow fever, Chikungunya, and Zika virus at a community level in the Gambella Region, South West Ethiopia. PLoS ONE. 2021;16. doi:10.1371/journal.pone.0253953

27. Ushijima Y, Abe H, Nguema Ondo G, Bikangui R, Massinga Loembé M, Zadeh VR, et al. Surveillance of the major pathogenic arboviruses of public health concern in Gabon, Central Africa: increased risk of West Nile virus and dengue virus infections. BMC Infect Dis. 2021;21: 265. doi:10.1186/s12879-021-05960-9

28. Sherman KE, Rouster SD, Kong LX, Shata TM, Archampong T, Kwara A, et al. Zika Virus Exposure in an HIV-Infected Cohort in Ghana. J Acquir Immune Defic Syndr. 2018;78: e35–e38. doi:10.1097/QAI.0000000000001718

29. Kisuya B, Masika MM, Bahizire E, Oyugi JO. Seroprevalence of Zika virus in selected regions in Kenya. Transactions of The Royal Society of Tropical Medicine and Hygiene. 2019;113: 735–739. doi:10.1093/trstmh/trz077

30. Chepkorir E, Tchouassi DP, Konongoi SL, Lutomiah J, Tigoi C, Irura Z, et al. Serological evidence of Flavivirus circulation in human populations in Northern Kenya: an assessment of disease risk 2016-2017. Virol J. 2019;16: 65. doi:10.1186/s12985-019-1176-y

31. Marchi S, Viviani S, Montomoli E, Tang Y, Boccuto A, Vicenti I, et al. Zika Virus in West Africa: A Seroepidemiological Study between 2007 and 2012. Viruses. 2020;12: 641. doi:10.3390/v12060641

32. Diarra I, Nurtop E, Sangaré AK, Sagara I, Pastorino B, Sacko S, et al. Zika Virus Circulation in Mali. Emerg Infect Dis. 2020;26: 945–952. doi:10.3201/eid2605.191383

33. Mathé P, Egah DZ, Müller JA, Shehu NY, Obishakin ET, Shwe DD, et al. Low Zika virus seroprevalence among pregnant women in North Central Nigeria, 2016. Journal of Clinical Virology. 2018;105: 35–40. doi:10.1016/j.jcv.2018.05.011

34. Kolawole OM, Suleiman MM, Bamidele EP. Molecular epidemiology of Zika virus and Rubella virus in pregnant women attending Sobi Specialist Hospital Ilorin, Nigeria. International Journal of Research in Medical Sciences. 2020;8: 2275–2283. doi:10.18203/2320-6012.ijrms20202234

35. Shaibu JO, Okwuraiwe AP, Jakkari A, Dennis A, Akinyemi KO, Li J, et al. Sero-molecular Prevalence of Zika Virus among Pregnant Women Attending Some Public Hospitals in Lagos State, Nigeria. European Journal of Medical and Health Sciences. 2021;3: 77–82. doi:10.24018/ejmed.2021.3.5.1075

36. Anejo-Okopi J, Gotom DY, Chiehiura NA, Okojokwu JO, Amanyi DO, Egbere JO, et al. The Seroprevalence of Zika Virus Infection among HIV Positive and HIV Negative Pregnant Women in Jos, Nigeria. Hosts and Viruses. 637134336000000000;7. doi:10.17582/journal.hv/2020/7.6.129.136

37. Mac PA, Kroeger A, Daehne T, Anyaike C, Velayudhan R, Panning M. Zika, Flavivirus and Malaria Antibody Cocirculation in Nigeria. Tropical Medicine and Infectious Disease. 2023;8: 171. doi:10.3390/tropicalmed8030171

38. Nurtop E, Moyen N, Dzia-Lepfoundzou A, Dimi Y, Ninove L, Drexler JF, et al. A Report of Zika Virus Seroprevalence in Republic of the Congo. Vector Borne Zoonotic Dis. 2020;20: 40–42. doi:10.1089/vbz.2019.2466

39. Soghaier MA, Abdelgadir DM, Abdelkhalig SM, Kafi H, Zarroug IMA, Sall AA, et al. Evidence of pre-existing active Zika virus circulation in Sudan prior to 2012. BMC Res Notes. 2018;11: 906. doi:10.1186/s13104-018-4027-9

40. Mwanyika GO, Sindato C, Rugarabamu S, Rumisha SF, Karimuribo ED, Misinzo G, et al. Seroprevalence and associated risk factors of chikungunya, dengue, and Zika in eight districts in Tanzania. Int J Infect Dis. 2021;111: 271–280. doi:10.1016/j.ijid.2021.08.040

41. Chisenga CC, Bosomprah S, Musukuma K, Mubanga C, Chilyabanyama ON, Velu RM, et al. Sero-prevalence of arthropod-borne viral infections among Lukanga swamp residents in Zambia. PLoS One. 2020;15: e0235322. doi:10.1371/journal.pone.0235322

42. Babaniyi OA, Mwaba P, Songolo P, Mazaba-Liwewe ML, MweeneNdumba I, Masaninga F, et al. Seroprevalence of Zika virus infection specific IgG in Western and North-Western Provinces of Zambia. International Journal of Public Health and Epidemiology. 2014;4: 1–6.

43. Saba Villarroel PM, Nurtop E, Pastorino B, Roca Y, Drexler JF, Gallian P, et al. Zika virus epidemiology in Bolivia: A seroprevalence study in volunteer blood donors. PLoS Negl Trop Dis. 2018;12: e0006239. doi:10.1371/journal.pntd.0006239

44. Slavov SN, Guaragna Machado RR, Ferreira AR, Soares CP, Araujo DB, Leal Oliveira DB, et al. Zika virus seroprevalence in blood donors from the Northeastern region of São Paulo State, Brazil, between 2015 and 2017. Journal of Infection. 2020;80: 111–115. doi:10.1016/j.jinf.2019.10.002

45. Salgado BB, de Jesus Maués FC, Pereira RL, Chiang JO, de Oliveira Freitas MN, Ferreira MS, et al. Prevalence of arbovirus antibodies in young healthy adult population in Brazil. Parasit Vectors. 2021;14: 403. doi:10.1186/s13071-021-04901-4

46. Salgado BB, Maués FC de J, Jordão M, Pereira RL, Toledo-Teixeira DA, Parise PL, et al. Antibody cross-reactivity and evidence of susceptibility to emerging Flaviviruses in the dengue-endemic Brazilian Amazon. Int J Infect Dis. 2023;129: 142–151. doi:10.1016/j.ijid.2023.01.033

47. Netto EM, Moreira-Soto A, Pedroso C, Höser C, Funk S, Kucharski AJ, et al. High Zika Virus Seroprevalence in Salvador, Northeastern Brazil Limits the Potential for Further Outbreaks. mBio. 2017;8: e01390-17. doi:10.1128/mBio.01390-17

48. Moreira-Soto A, de Souza Sampaio G, Pedroso C, Postigo-Hidalgo I, Berneck BS, Ulbert S, et al. Rapid decline of Zika virus NS1 antigen-specific antibody responses, northeastern Brazil. Virus Genes. 2020;56: 632–637. doi:10.1007/s11262-020-01772-2

49. Alves LV, Leal CA, Alves JGB. Zika virus seroprevalence in women who gave birth during Zika virus outbreak in Brazil - a prospective observational study. Heliyon. 2020;6: e04817. doi:10.1016/j.heliyon.2020.e04817

50. Diefenbach CF, Slavov SN, Kashima S, Ferreira AR, Hespanhol MR, Bandeira BS, et al. Prevalence of Zika Virus (Zikv) in blood donors from a hemotherapy service of the southern region of Brazil. ISBT Science Series. 2019;14: 157–162. doi:10.1111/voxs.12436

51. Wittlin BB, Almeida DV de, Marques BCL, Monteiro CC, Moreira LF de S, Linhares JHR, et al. Chikungunya, Zika and Dengue seroprevalence rates among pregnant women in a hospital of southeastern Brazil. Soroprevalência de chikungunya, zika e dengue em gestantes de um hospital do sudeste do Brasil. 2021 [cited 6 Apr 2024]. Available: https://www.arca.fiocruz.br/handle/icict/56611

52. Barreto FK de A, Alencar CH, Araújo FM de C, Oliveira R de MAB, Cavalcante JW, Lemos DRQ, et al. Seroprevalence, spatial dispersion and factors associated with flavivirus and chikungunha infection in a risk area: a population-based seroprevalence study in Brazil. BMC Infect Dis. 2020;20: 881. doi:10.1186/s12879-020-05611-5

53. Périssé ARS, Souza-Santos R, Duarte R, Santos F, de Andrade CR, Rodrigues NCP, et al. Zika, dengue and chikungunya population prevalence in Rio de Janeiro city, Brazil, and the importance of seroprevalence studies to estimate the real number of infected individuals. PLoS One. 2020;15: e0243239. doi:10.1371/journal.pone.0243239

54. Braga C, Martelli CMT, Souza WV, Luna CF, Albuquerque M de FPM, Mariz CA, et al. Seroprevalence of Dengue, Chikungunya and Zika at the epicenter of the congenital microcephaly epidemic in Northeast Brazil: A population-based survey. PLOS Neglected Tropical Diseases. 2023;17: e0011270. doi:10.1371/journal.pntd.0011270

55. Francisco MV, Costa B, Almeida B, Santos C, Casaes AC, Santos YD, et al. Seroprevalence of Zika, Chikungunya and Dengue viruses in a rural area of northeastern Brazil. International Journal of Infectious Diseases. 2020;101: 245. doi:10.1016/j.ijid.2020.11.074

56. Araújo TMD, Souza FDO, Helioterio MC, Andrade KVFD, Pinho PDS, Werneck GL. The high prevalence of infectious diseases among health workers indicates the need for improving surveillance. Rev bras saúde ocup. 2023;48: e17. doi:10.1590/2317-6369/23021en2023v48e17

57. Bayona-Pacheco B, Acosta-Reyes J, Navarro E, San-Juan H, Bula J, Baquero H. Seroprevalence of Zika virus among blood donors before the epidemic in Barranquilla, Colombia, 2015-2016. An Acad Bras Cienc. 2019;91: e20180860. doi:10.1590/0001-3765201920180860

58. Marbán-Castro E, Arrieta GJ, Martínez MJ, González R, Bardají A, Menéndez C, et al. High Seroprevalence of Antibodies against Arboviruses among Pregnant Women in Rural Caribbean Colombia in the Context of the Zika Virus Epidemic. Antibodies (Basel). 2020;9: 56. doi:10.3390/antib9040056

59. Rivas E, Ojeda J, Garcia-Rivera EJ, Rivera DM, Arredondo JL, Medina EL, et al. Prospective surveillance of Zika virus at the end of the Americas' outbreak: An unexpected outcome. Frontiers in Tropical Diseases. 2022;3. Available: https://www.frontiersin.org/articles/10.3389/fitd.2022.1027908

60. Cardona-Ospina JA, Trujillo AM, Jiménez-Posada EV, Sepúlveda-Arias JC, Tabares-Villa FA, Altieri-Rivera JS, et al. Susceptibility to endemic Aedes-borne viruses among pregnant women in Risaralda, Colombia. Int J Infect Dis. 2022;122: 832–840. doi:10.1016/j.ijid.2022.07.017

61. Flamand C, Bailly S, Fritzell C, Berthelot L, Vanhomwegen J, Salje H, et al. Impact of Zika Virus Emergence in French Guiana: A Large General Population Seroprevalence Survey. The Journal of Infectious Diseases. 2019;220: 1915–1925. doi:10.1093/infdis/jiz396

62. Lamb MM, Paniagua-Avila A, Zacarias A, Rojop N, Chacon A, Natrajan MS, et al. Repeated Rapid Active Sampling Surveys Demonstrated a Rapidly Changing Zika Seroprevalence among Children in a Rural Dengue-endemic Region in Southwest Guatemala during the Zika Epidemic (2015-2016). Am J Trop Med Hyg. 2022;107: 1099–1106. doi:10.4269/ajtmh.22-0399

63. Anzinger JJ, Mears CD, Ades AE, Francis K, Phillips Y, Leys YE, et al. Antenatal Seroprevalence of Zika and Chikungunya Viruses, Kingston Metropolitan Area, Jamaica, 2017-2019. Emerg Infect Dis. 2022;28: 473–475. doi:10.3201/eid2802.211849

64. Gallian P, Cabié A, Richard P, Paturel L, Charrel RN, Pastorino B, et al. Zika virus in asymptomatic blood donors in Martinique. Blood. 2017;129: 263–266. doi:10.1182/blood-2016-09-737981

65. Eligio-García L, Crisóstomo-Vázquez M del P, Caballero-García M de L, Soria-Guerrero M, Méndez–Galván JF, López-Cancino SA, et al. Co-infection of Dengue, Zika and Chikungunya in a group of pregnant women from Tuxtla Gutiérrez, Chiapas: Preliminary data. 2019. PLOS Neglected Tropical Diseases. 2020;14: e0008880. doi:10.1371/journal.pntd.0008880

66. Collins MH, Zepeda O, Blette B, Jadi R, Morales M, Pérez R, et al. Serologic surveillance of maternal Zika infection in a prospective cohort in Leon, Nicaragua during the peak of the Zika epidemic. PLoS One. 2020;15: e0230692. doi:10.1371/journal.pone.0230692

67. Zambrana JV, Bustos Carrillo F, Burger-Calderon R, Collado D, Sanchez N, Ojeda S, et al. Seroprevalence, risk factor, and spatial analyses of Zika virus infection after the 2016 epidemic in Managua, Nicaragua. Proc Natl Acad Sci U S A. 2018;115: 9294–9299. doi:10.1073/pnas.1804672115

68. Zepeda O, Espinoza DO, Martinez E, Cross KA, Becker-Dreps S, de Silva AM, et al. Antibody Immunity to Zika Virus among Young Children in a Flavivirus-Endemic Area in Nicaragua. Viruses. 2023;15: 796. doi:10.3390/v15030796

69. Cachay R, Schwalb A, Acevedo-Rodriguez JG, Merino X, Talledo M, Suarez-Ognio L, et al. Zika Virus Seroprevalence in Two Districts of Chincha, Ica, Peru: A Cross-Sectional Study. Am J Trop Med Hyg. 2021;106: 192–198. doi:10.4269/ajtmh.20-1339

70. Langerak T, Brinkman T, Mumtaz N, Arron G, Hermelijn S, Baldewsingh G, et al. Zika Virus Seroprevalence in Urban and Rural Areas of Suriname, 2017. J Infect Dis. 2019;220: 28–31. doi:10.1093/infdis/jiz063

71. Aubry M, Finke J, Teissier A, Roche C, Broult J, Paulous S, et al. Seroprevalence of arboviruses among blood donors in French Polynesia, 2011–2013. International Journal of Infectious Diseases. 2015;41: 11–12. doi:10.1016/j.ijid.2015.10.005

72. Henderson AD, Aubry M, Kama M, Vanhomwegen J, Teissier A, Mariteragi-Helle T, et al. Zika seroprevalence declines and neutralizing antibodies wane in adults following outbreaks in French Polynesia and Fiji. Elife. 2020;9: e48460. doi:10.7554/eLife.48460

73. Cauchemez S, Besnard M, Bompard P, Dub T, Guillemette-Artur P, Eyrolle-Guignot D, et al. Association between Zika virus and microcephaly in French Polynesia, 2013-15: a retrospective study. Lancet. 2016;387: 2125–2132. doi:10.1016/S0140-6736(16)00651-6

74. Aubry M, Teissier A, Huart M, Merceron S, Vanhomwegen J, Roche C, et al. Zika Virus Seroprevalence, French Polynesia, 2014-2015. Emerg Infect Dis. 2017;23: 669–672. doi:10.3201/eid2304.161549

75. Kama M, Aubry M, Naivalu T, Vanhomwegen J, Mariteragi-Helle T, Teissier A, et al. Sustained Low-Level Transmission of Zika and Chikungunya Viruses after Emergence in the Fiji Islands. Emerg Infect Dis. 2019;25: 1535–1538. doi:10.3201/eid2508.180524

76. Grant R, Kizu J, Graham M, McCallum F, McPherson B, Auliff A, et al. Serological evidence of possible high levels of undetected transmission of Zika virus among Papua New Guinea military personnel, 2019. IJID Regions. 2022;4: 131–133. doi:10.1016/j.ijregi.2022.07.006

77. Russell TL, Horwood PF, Harrington H, Apairamo A, Kama NJ, Bobogare A, et al. Seroprevalence of dengue, Zika, chikungunya and Ross River viruses across the Solomon Islands. PLoS Negl Trop Dis. 2022;16: e0009848. doi:10.1371/journal.pntd.0009848

Articles with a seroprevalence of 0.0% were not included
