# Supplementary material for: Global seroprevalence of Zika virus in asymptomatic individuals: A systematic review
Source: PLoS Negl Trop Dis. 2024 Apr 17;18(4):e0011842. doi: 10.1371/journal.pntd.0011842 (PMC11057727; doi:10.1371/journal.pntd.0011842)
Supplement: Attachment — Submitted filename: PNTD-D-23-01541.pdf [file pntd.0011842.s009.pdf]

PLOS Neglected Tropical Diseases

Global seroprevalence of Zika virus in asymptomatic individuals: A systematic review

--Manuscript Draft--

**Manuscript Number:**	PNTD-D-23-01541
**Full Title:**	Global seroprevalence of Zika virus in asymptomatic individuals: A systematic review
**Short Title:**	Seroprevalence of Zika virus
**Article Type:**	Research Article
**Keywords:**	Zika virus; ZIKV; seroprevalence; arboviruses
**Abstract:**	Background<br><br>Zika virus (ZIKV) has spread to five of the six World Health Organization (WHO) regions. Given the substantial number of asymptomatic infections and clinical presentations resembling those of other arboviruses, estimating the true burden of ZIKV infections is both challenging and essential. Therefore, we conducted a systematic review and meta-analysis of seroprevalence studies of ZIKV IgG in asymptomatic population to estimate its global impact and distribution.<br><br>Methodology/principal findings<br><br>We conducted extensive searches and compiled a collection of articles published from Jan/01/2000, to Jul/31/2023, from Embase, Pubmed, SciELO and Scopus databases. The random effects model was used to pool prevalences, reported with their 95% confidence interval (CI), the modified tool of Hoy et al., 2012 for risk bias, and the I2 method for heterogeneity (PROSPERO registration No. CRD42023442227). Eighty-four studies from 49 countries/territories, with a diversity of study designs and serological tests were included. The global seroprevalence of ZIKV was 21.0% (95%CI 16.1%-26.4%). Evidence of IgG antibodies was identified in all WHO regions, except for Europe. Seroprevalence correlated with the epidemics in the Americas (39.9%, 95%CI:30.0-49.9), and in some Western Pacific countries (15.6%, 95%CI:8.2-24.9), as well as with recent and past circulation in Southeast Asia (22.8%, 95%CI:16.5-29.7), particularly in Thailand. Additionally, sustained low circulation was observed in Africa (8.4%, 95%CI:4.8-12.9), except for Gabon (43.7%), and Burkina Faso (22.8%). Although no autochthonous transmission was identified in the Eastern Mediterranean, a seroprevalence of 16.0% was recorded.<br><br>Conclusions/significance<br><br>The study highlights the high heterogeneity and gaps in the distribution of seroprevalence. The implementation of standardized protocols and the development of tests with high specificity are essential for ensuring a valid comparison between studies. Equally crucial are vector surveillance and control methods to reduce the risk of emerging and re-emerging ZIKV outbreaks, whether caused by Ae. aegypti or Ae. albopictus or by the Asian or African ZIKV.
**Additional Information:**	
**Question**	**Response**
**Government Employee**<br><br><br>Are you or any of the contributing authors an employee of the United States government?<br><br><br>Manuscripts authored by one or more US Government employees are not	No - No authors are employees of the U.S. government.

copyrighted, but are licensed under a CC0 Public Domain Dedication, which allows unlimited distribution and reuse of the article for any lawful purpose. This is a legal requirement for US Government employees.<br><br>This will be typeset if the manuscript is accepted for publication.	
**Financial Disclosure**<br><br>Enter a financial disclosure statement that describes the sources of funding for the work included in this submission. Review the submission guidelines for detailed requirements. View published research articles from *PLOS Neglected Tropical Diseases* for specific examples.<br><br>This statement is required for submission and **will appear in the published article** if the submission is accepted. Please make sure it is accurate.<br><br>**Funded studies**<br>Enter a statement with the following details:<br>• Initials of the authors who received each award<br>• Grant numbers awarded to each author<br>• The full name of each funder<br>• URL of each funder website<br>• Did the sponsors or funders play any role in the study design, data collection and analysis, decision to publish, or preparation of the manuscript?<br><br>Did you receive funding for this work?	Yes
Please add funding details.<br>as follow-up to "**Financial Disclosure**<br><br>Enter a financial disclosure statement that describes the sources of funding for the work included in this submission. Review the submission guidelines for detailed requirements. View published research articles from *PLOS Neglected Tropical Diseases* for specific examples.	This work was financially supported by the National Research Council of Thailand (NRCT): NRCT5-RGJ63012-125, Grant No. RGNS 64-172 by Office of the Permanent Secretary, Ministry of Higher Education, Science, Research and Innovation, (MHESI) (N.G.) and international postdoctoral fellowship 2022 provided by Mahidol University (P.M.S.V).<br>The funders had no role in the study design, data collection, analysis or decision to publish, nor in the preparation of the manuscript.

This statement is required for submission and **will appear in the published article** if the submission is accepted. Please make sure it is accurate.<br><br>**Funded studies**<br>Enter a statement with the following details:<br>• Initials of the authors who received each award<br>• Grant numbers awarded to each author<br>• The full name of each funder<br>• URL of each funder website<br>• Did the sponsors or funders play any role in the study design, data collection and analysis, decision to publish, or preparation of the manuscript?<br><br>Did you receive funding for this work?"	
Please select the country of your main research funder (please select carefully as in some cases this is used in fee calculation).<br>as follow-up to "**Financial Disclosure**<br><br>Enter a financial disclosure statement that describes the sources of funding for the work included in this submission. Review the submission guidelines for detailed requirements. View published research articles from *PLOS Neglected Tropical Diseases* for specific examples.<br><br>This statement is required for submission and **will appear in the published article** if the submission is accepted. Please make sure it is accurate.	THAILAND - TH

**Funded studies**<br>Enter a statement with the following details:<br>• Initials of the authors who received each award<br>• Grant numbers awarded to each author<br>• The full name of each funder<br>• URL of each funder website<br>• Did the sponsors or funders play any role in the study design, data collection and analysis, decision to publish, or preparation of the manuscript?<br><br>Did you receive funding for this work?"	
**Competing Interests**<br><br>On behalf of all authors, disclose any competing interests that could be perceived to bias this work.<br><br>This statement will be typeset if the manuscript is accepted for publication.<br><br>Review the instructions link below and PLOS NTDs' competing interests policy to determine what information must be disclosed at submission.	Authors declare no competing interests.
**Data Availability**<br><br>Provide a **Data Availability Statement** in the box below. This statement should detail where the data used in this submission can be accessed. This statement will be typeset if the manuscript is accepted for publication.<br><br>Before publication, authors are required to make all data underlying their findings fully available, without restriction. Review our PLOS Data Policy page for detailed information on this policy. Instructions for writing your Data Availability statement can be accessed via the Instructions link below.	The data that support the findings of the study are available within the article. Additional data is available upon request from the corresponding author.

Powered by Editorial Manager® and ProduXion Manager® from Aries Systems Corporation

Global seroprevalence of Zika virus in asymptomatic individuals: A systematic review

Paola Mariela Saba Villarroel[1,2], Rodolphe Hamel[1,2,3], Nuttamonpat Gumpangseth[1,2], Sakda Yainoy[1], Phanit Koomhin[4], Dorothée Missé[3], Sineewanlaya Wichit[1,2*]

[1]Department of Clinical Microbiology and Applied Technology, Faculty of Medical Technology, Mahidol University, Nakhon Pathom, Thailand

[2]Viral Vector Joint unit and Joint Laboratory, Mahidol University, Nakhon Pathom, Thailand

[3]MIVEGEC, Univ. Montpellier, CNRS, IRD, Montpellier, France

[4]Center of Excellence in Innovation on Essential Oil, Walailak University, Nakhonsithammarat, Thailand

*Corresponding author: sineewanlaya.wic@mahidol.ac.th (SW)

Short title: Seroprevalence of Zika virus

Abstract

Background

Zika virus (ZIKV) has spread to five of the six World Health Organization (WHO) regions. Given the substantial number of asymptomatic infections and clinical presentations resembling those of other arboviruses, estimating the true burden of ZIKV infections is both challenging and essential. Therefore, we conducted a systematic review and meta-analysis of seroprevalence studies of ZIKV IgG in asymptomatic population to estimate its global impact and distribution.

Methodology/principal findings

We conducted extensive searches and compiled a collection of articles published from Jan/01/2000, to Jul/31/2023, from Embase, Pubmed, SciELO and Scopus databases. The random effects model was used to pool prevalences, reported with their 95% confidence interval (CI), the modified tool of Hoy *et al.*, 2012 for risk bias, and the I^2 method for heterogeneity (PROSPERO registration No. CRD42023442227). Eighty-four studies from 49 countries/territories, with a diversity of study designs and serological tests were included. The global seroprevalence of ZIKV was 21.0% (95%CI 16.1%-26.4%). Evidence of IgG antibodies was identified in all WHO regions, except for Europe. Seroprevalence correlated with the epidemics in the Americas (39.9%, 95%CI:30.0-49.9), and in some Western Pacific countries (15.6%, 95%CI:8.2-24.9), as well as with recent and past circulation in Southeast Asia (22.8%, 95%CI:16.5-29.7), particularly in Thailand. Additionally, sustained low circulation was observed in Africa (8.4%, 95%CI:4.8-12.9), except for Gabon (43.7%), and Burkina Faso (22.8%). Although no autochthonous transmission was identified in the Eastern Mediterranean, a seroprevalence of 16.0% was recorded.

Conclusions/significance

The study highlights the high heterogeneity and gaps in the distribution of seroprevalence. The implementation of standardized protocols and the development of tests with high specificity are essential for ensuring a valid comparison between studies. Equally crucial are vector surveillance and control methods to reduce the risk of emerging and re-emerging ZIKV outbreaks, whether caused by *Ae. aegypti* or *Ae. albopictus* or by the Asian or African ZIKV.

Author summary

Zika virus (ZIKV) remains an important public health concern. Estimating the true burden of the disease is a major challenge, often underestimated due to the substantial number of asymptomatic infections. Consequently, seroprevalence studies are valuable for determining the geographic extent of the virus, measuring levels of human immunity, and assessing potential infection-related risks. Thus, we conducted a systematic review and meta-analysis of the literature addressing the seroprevalence of ZIKV in asymptomatic individuals worldwide. The overall seroprevalence of ZIKV IgG antibodies was 21.0%, calculated based on 84 studies published between January 2000 and July 2023. Antibodies have been found in all WHO regions, but none in Europe, with rates ranging from 8.4% in Africa to 39.9% in America. Further standardized surveillance studies are needed to understand immunity per region and over time, evaluate vector dynamics, and assess the risk of future outbreaks.

Introduction

Zika virus (ZIKV) is an arthropod-borne virus classified within the *Flaviviridae* family. It is mainly transmitted to humans through the bite of infected *Aedes aegypti* and *Aedes albopictus* mosquitoes. The first human ZIKV infections dates back to the 1950s. Subsequent serological studies revealed sporadic infections in African and Asian countries and territories over the following five decades. However, a transformative event unfolded after May 2007 when ZIKV emerged as an important human pathogen responsible for substantial human outbreaks in the Pacific Islands, followed by an explosive and rapid dissemination across the Americas during 2015-2016. Phylogenetic analysis has identified two major ZIKV lineages: the African lineage

(East and West African sublineages), and the Asian lineage (Asian and American sublineages), with the latter being responsible for the recent outbreaks [1–3].

ZIKV continues to pose a significant global threat, characterized by its persistent geographic expansion. According to the most recent data available, a total of 89 countries and territories, among five of the six WHO regions have documented autochthonous ZIKV transmission. The Eastern Mediterranean Region is the only exception [4].

Approximately 80% of ZIKV infections typically manifest as asymptomatic, and when symptoms do occur, they are usually self-limited and short-lasting. However, during recent ZIKV disease outbreaks, complications including Guillain-Barré syndrome (GBS) and congenital Zika syndrome (CZS) have been documented. Additionally, other modes of transmission have been described, aggravated by the absence of specific treatments of effective vaccines [5].

The current disease surveillance system heavily relies on data collected from health centers and laboratories, primarily focusing on symptomatic cases. While this approach facilitates the early detection of outbreaks, it is not designed to provide an accurate estimation of the true disease burden. In light of these limitations, seroprevalence studies assume a pivotal role in estimating population immunity, providing insights into geographic impact and distribution. Moreover, they contribute significantly to the evaluation and guidance of disease prevention and control strategies. Nevertheless, conducting seroprevalence studies poses significant challenges, mainly due to the extensive serological cross-reactivity observed between flaviviruses, notably with dengue virus (DENV). Consequently, additional tests to improve specificity are necessary, such as seroneutralization methods. However, these methods are characterized by being time-consuming, costly, labor-intensive, and are often not readily available [1,6].

The objective of our study was to perform a systematic review and meta-analysis of published studies on the seroprevalence of ZIKV IgG or IgM/IgG antibodies in asymptomatic populations between January 2000 to July 2023. Our study aimed to provide an update of a previous systematic review [6] conducted in a limited number of studies (n=12) for a better understanding of the global distribution, prevalence rates, risk factors, and changes over time of ZIKV.

Methods

This systematic review and meta-analysis were conducted in accordance with the PRISMA guidelines (S1 table). This study is registered on the PROSPERO International prospective register of systematic reviews platform, under registration number CRD42023442227.

Search strategy

We conducted a comprehensive and exhaustive search for articles published between January 01, 2000 to July 31, 2023 from Embase, Pubmed, SciELO and Scopus databases. To perform this search, we utilized the following Medical Subject Headings (MeSH) terms: “Zika virus”, “arboviruses”, “seroepidemiologic studies”, “serologic”, “prevalence”. These search terms were combined using the Boolean operators, AND and OR. Our search was limited to studies involving humans and those published in English, French, Spanish or Portuguese languages. Additionally, we included relevant papers obtained from the references of the original articles. To ensure comprehensiveness, we also conducted a free search using the Google search engine.

Inclusion and exclusion criteria

We included cohort and cross-sectional studies of more than 88 participants in the sample size, except in cases where articles covered different time periods. Our inclusion criteria encompassed studies involving individuals from the general population, as well as specific population from all over the world. We considered studies that utilized any serological techniques to detect past ZIKV infection (IgG or IgM/IgG). The exclusion criteria comprised articles that assessed the same set of samples (the most complete version was included), reviews, studies involving febrile patients, suspected or confirmed infections, those not covering the study period, research involving travelers, migrants or non-human subjects, as well as case control studies.

Literature selection and data extraction

After removing duplicates, the titles and abstracts were screened by a review team member (PMSV). Full texts that met the potential inclusion criteria were evaluated by the same member (PMSV) and an independent reviewer (RH) to assess their eligibility based on the inclusion and exclusion criteria. Any disagreements were resolved through discussion with a third member (SW). Irrelevant articles were excluded, and the reasons for their exclusion were documented.

Data were extracted into an Excel spreadsheet, including the author's name, year of publication, sampling period, sample size, study design (cohort, cross-sectional), study location, setting (urban, rural, semi-urban), study population (general and specific population), age groups (adults [≥13 years old] and children [<13 years old]), proportion of female participants, diagnostic method, ZIKV strain (Asian or African), number of positive cases, and data interpretation. Furthermore, any available risk factors for positivity were extracted. In cases involving multinational studies or studies conducted in different periods, the information was separated by

country and/or year. Data were categorized according to World Health Organization (WHO) regions, which include Africa, the Americas, the Eastern Mediterranean, Europe, Southeast Asia, and the Western Pacific. Additionally, they were grouped into different sampling periods, including the entire period, as well as specific time frames (Jan 2000-Apr 2015 and May 2015-Jul 2023).

Study quality assessment

The bias risk in the included studies was assessed using a modified version of the tool designed by Hoy *et al.*, 2012, which includes a 10-item checklist [7]. Each item was assigned a score of "0" for "no" or "unclear" and "1" for "yes". A total score of 7-9 was considered "low risk", 4-6 "moderate risk", and 0-3 "high risk" of bias. Two authors independently evaluated the risk bias (PMSV and RH). Any disagreements were resolved through discussion and consultation with a third author (SW).

Statistical analysis

Continuous variables were presented as means and standard deviation (SD), while categorical variables were expressed as numbers and percentages. The seroprevalences and their corresponding 95% confidence intervals (CIs) were calculated using the raw data.

In the meta-analysis, the I^2 method was employed to assess heterogeneity. An I^2 value of 25.0%, 50.0%, and 75.0% indicated low, moderate, high heterogeneity, respectively. The data were converted using Freeman-Tukey transformation to satisfy the normal distribution assumption. A random-effects model was chosen for studies with moderate to high heterogeneity;

otherwise, a fixed-effects model was used. Furthermore, Egger's test was used to evaluate the potential presence of publication bias.

The proportion of ZIKV seropositivity in each study was combined to derive a pooled seroprevalence of ZIKV worldwide. The data were further categorized and analyzed according to WHO regions and based on sampling years (before and after the first ZIKV case detected in Sao Paulo, Brazil in May 2015). Moreover, subgroup analyses were performed based on the study population, age groups, settings, diagnostic methods, and ZIKV strains.

The statistical analyses were performed using SPSS 13.0 software (SPSS, Inc., Chicago, IL). The $\chi 2$ test was used for group comparisons, considering statistical significance when the p-value was <0.05. To generate forest plots, calculate CIs, and evaluate heterogeneity, JBI Sumari and MedCalc software's were used. Additionally, maps were created using Mapchart.net to provide a better visualization of seroprevalence estimates per country and administrative divisions, as well as the number of studies conducted in each country.

Results

Literature search

The process of selecting studies reporting ZIKV seroprevalences is illustrated in Fig 1. Initially, a database search yielded a total of 1682 records. After removing duplicates, 884 articles underwent evaluation based on their titles and abstracts, resulting in the identification of 104 articles that were eligible for full-text review. Among these studies, those that did not meet the inclusion criteria were subsequently excluded, leading to a final inclusion of 84 articles with 113 data points when divided by year and country.

Fig 1. Flowchart of the selection process of the studies included in the analysis of Zika virus seroprevalence.

General study characteristics and quality assessment

Seroprevalence studies were conducted in 49 countries and territories, with a total of 63,864 individuals involved. These studies comprised 18 countries in Africa (including Burkina Faso [8], Cape Verde [9], Cameroon [10], Democratic Republic of the Congo [11], Ethiopia [12], Gabon [13], Ghana [14], Kenya [15,16], Madagascar [17], Mali [18,19], Nigeria [20–24], Republic of the Congo [25], Rwanda [26], Senegal [18], Sudan [27], Tanzania [28], The Gambia [18], and Zambia [29,30]); 13 countries in the Americas (Bolivia [1], Brazil, Colombia, French Guiana (see references in S2 Table), Guatemala [31], Honduras [32], Jamaica [33], Martinique [34], Mexico [32,35], Nicaragua (references in S2 Table), Peru [36], Puerto Rico [32], and Suriname [37]); 3 countries in the Eastern Mediterranean (Iran [38], Iraq [39], Saudi Arabia [40]); 3 countries in Europe (Cyprus [41], France [42] and Sweden [26]); 2 countries in Southeast Asia (Indonesia, Thailand (references in S2 Table); and 10 countries in the Western Pacific region (China [43–45], Fiji [46,47], French Polynesia (references in S2 Table), Lao PDR [48], Malaysia [49–51], Papua New Guinea [52], Solomon Islands [53], Taiwan [54], The Philippines [55], and Vietnam [56,57]). The majority of these studies were carried out within the tropical zone, with Brazil (n=13) and Thailand (n=7) being the most actively involved in survey implementation over the last century. Furthermore, more than 80.0% of the studies were published during and after 2019. The number of studies per country or territory is shown in Table 1 and Fig 2A.

Table 1. Seroprevalence of Zika virus per World Health Organization region and per study period.

WHO region	Country	Studies per country	Study period					
		No.	Jan 2000-Jul 2023		Jan 2000-Apr 2015		May 2015-Jul 2023	
			Prevalence		Prevalence		Prevalence	
			%	95%CI	%	95%CI	%	95%CI
Worldwide (84 studies)	Overall	91	21.0	16.1-26.4	12.6	6.4-20.4	25.7	20.5-31.3
Africa	All	25	8.4	4.8-12.9	5.1	1.2-11.2	15.0	9.2-21.9
	Burkina Faso	1	22.8	19.2-26.5	NA	NA	22.8	19.2-26.5
	Cabo Verde	1	10.9	8.1-14.0	NA	NA	10.9	8.1-14.0
	Cameroon	1	4.9	3.7-6.3	NA	NA	4.9	3.7-6.3
	Democratic Republic of the Congo	1	0.1	0.0-0.4	0.1	0.0-0.4	NA	NA
	Ethiopia	1	27.3	20.5-34.8	NA	NA	27.3	20.5-34.8
	Gabon	1	43.7	33.7-53.9	52.6	36.6-68.4	41.6	30.4-53.1
	Ghana	1	13.1	9.1-17.8	13.1	9.1-17.8	NA	NA
	Kenya	2	1.5	0.0-7.1	0.2	0.0-0.7	3.9	2.7-5.3
	Madagascar	1	0.0	NA	0.0	NA	NA	NA
	Mali	2	7.0	1.7-15.6	5.6	3.0-16.5	12.0	9.8-14.3
	Nigeria	5	13.1	6.6-21.3	NA	NA	13.1	6.6-21.3
	Republic of the Congo	1	1.8	0.7-3.4	1.8	0.7-3.4	NA	NA
	Rwanda	1	1.4	0.7-2.3	NA	NA	1.4	0.7-2.3
	Senegal	1	10.1	4.8-17.0	10.1	4.8-17.0	NA	NA
	Sudan	1	0.1	0.0-0.2	0.1	0.0-0.2	NA	NA
	Tanzania	1	6.7	5.5-7.9	NA	NA	6.7	5.5-7.9
	The Gambia	1	1.8	0.0-6.2	1.8	0.0-6.2	NA	NA
	Zambia	2[a]	7.9	3.9-13.1	NA	NA	10.7	6.9-15.3
America	All	31	39.9	30.3-49.9	0.0	NA	39.9	30.3-49.9
	Bolivia	1	29.1	26.0-32.3	NA	NA	29.1	26.0-32.3
	Brazil	13	36.9	24.9-49.8	0.0	NA	36.9	24.9-49.8
	Colombia	4	56.6	15.7-92.7	NA	NA	56.6	15.7-92.7
	French Guiana	1	23.3	21.7-24.9	NA	NA	23.3	21.7-24.9
	Guatemala	1	21.8	17.8-26.1	NA	NA	21.8	17.8-26.1
	Honduras	1	73.8	69.9-77.6	NA	NA	73.8	69.9-77.6

	Jamaica	1	15.6	12.7-18.6	NA	NA	15.6	12.7-18.6
	Martinique	1	21.9	18.6-25.3	NA	NA	21.9	18.6-25.3
	Mexico	2	60.1	56.9-63.2	NA	NA	60.1	56.9-63.2
	Nicaragua	3	57.0	37.8-75.1	NA	NA	57.0	37.8-75.1
	Peru	1	13.0	9.9-16.5	NA	NA	13.0	9.9-16.5
	Puerto Rico	1	34.0	29.4-38.7	NA	NA	34.0	29.4-38.7
	Suriname	1	35.1	31.7-38.5	NA	NA	35.1	31.7-38.5
Eastern Mediterranean	All	3	16.0	10.1-23.1	NA	NA	16.0	10.1-23.1
	Iran	1	0.0	NA	NA	NA	0.0	NA
	Iraq	1	12.0	6.5-18.9	NA	NA	12.0	6.5-18.9
	Saudi Arabia	1	18.5	14.9-22.5	NA	NA	18.5	14.9-22.5
Europe	All	3	0.0	NA	NA	NA	0.0	NA
	Cyprus	1	0.0	NA	NA	NA	0.0	NA
	France	1	0.0	NA	NA	NA	0.0	NA
	Sweden	1	0.0	NA	NA	NA	0.0	NA
Southeast Asia	All	10	22.8	16.5-29.7	23.3	13.6-34.5	22.1	16.4-28.4
	Indonesia	3	9.1	7.7-10.6	9.1	7.7-10.6	0.0	NA
	Thailand	7	25.9	19.5-32.8	29.3	18.7-41.1	22.1	16.4-28.4
Western Pacific	All	19	15.6	8.2-24.9	16.1	3.0-36.5	15.5	6.8-26.8
	China	3	2.1	0.0-11.9	NA	NA	2.1	0.0-11.9
	Fiji	2	13.7	7.4-21.4	7.5	5.9-9.3	19.2	12.5-26.9
	French Polynesia	4	40.7	26.6-67.4	40.0	8.2-77.5	42.1	7.5-82.0
	Lao PDR	1	7.0	2.7-13.2	4.5	2.5-6.9	9.9	7.8-12.2
	Malaysia	3	7.1	0.7-19.0	1.7	0.2-4.2	7.7	0.0-37.8
	Papua New Guinea	1	64.9	58.3-71.3	NA	NA	64.9	58.3-71.3
	Solomon Islands	1	28.1	25.4-30.9	NA	NA	28.1	25.4-30.9
	Taiwan	1	0.5	0.0-2.0	NA	NA	0.5	0.0-2.0
	The Philippines	1	17.9	14.8-21.2	NA	NA	17.9	14.8-21.2
	Vietnam	2	1.0	0.4-1.7	0.6	0.0-2.4	1.1	0.5-2.0

NA: No data available. [a]One study: No data of year

Fig 2. (A) Number of Zika virus seroprevalence studies per country. (B) Seroprevalence of Zika virus per country.

The m[illegible] were created using Mapch[illegible] https://www.mapchart.net/)

The cross-sectional observational research design, which is considered the most suitable for measuring prevalence, was the most frequently employed (78/84, 92.9%). Sample sizes ranged widely, from 88 to 5,880 individuals per study, with a mean of 582.2 ±135.93. As for study populations, the majority focused exclusively on the general population (34/84, 40.5%), while 19.0% centered on pregnant women, and 16.7% on blood donors. Among the eighty-three studies reporting age, 61.4% targeted adults, 32.6% encompassed all age groups, and 6.0% exclusively studied children. In terms of study settings, data were available for 67 studies. Among these, 64.2% were conducted in urban areas, 17.9% in both urban and rural settings, 11.9% in rural areas and the remaining studies in semi-urban or peri-urban. Furthermore, females accounted for 59.2% of the total participants.

The risk of bias was categorized as low in 76.2% and moderate in 23.8% of articles. The most significant issue in the evaluation was the absence of national population representation (Table S3).

Seroprevalence methods

Methods employed for the detection of IgG antibodies against ZIKV involved a wide range of techniques. These methods included both in-house and commercial enzyme-linked immunosorbent assay (ELISA), with a predominant usage of the commercial indirect Euroimmun-NS1 based kit. Other techniques included lateral flow immunoassays (LFA), blockade-of-binding assay (BOB), indirect immunofluorescent test (IIFT), colorimetric assays, Luminex bead-based assay, hemagglutination inhibition assay (HIA), microsphere immunoassay (MIA), and a variety of neutralization tests, such as plaque reduction neutralization tests (PRNT 50 & 90), focus reduction neutralization test (FRNT 50 & 90), and microneutralization assays (VNT).

For the interpretation of ZIKV seropositivity, ELISA tests were the most frequently used (34/83, 40.9%), followed by a combination of ELISA and a neutralization test (28/83, 33.7%), which included VNT (16/28, 57.1%), and well as PRNT or FRNT (12/28, 42.9%). In terms of ZIKV strains used for methodology, 36 out of 48 employed the Asian lineage, while 11 studies used the African lineage, and 1 Asian and African lineages.

Seroprevalence rates

In the meta-analysis, substantial heterogeneity was observed both in the overall data and among subgroups. The global ZIKV seroprevalence, determined using a random-effects model, was 21.0% (95%CI 16.1%-26.4%, I^2=99.6%, $p < 0.0001$) (S1 Fig). Egger's test yielded a p-value of 0.43, indicating no evidence of publication bias.

When dividing by WHO regions, the highest seroprevalence rate was observed in the Americas at 39.9% (95% CI: 30.3-49.9), followed by Southeast Asia at 22.8% (95% CI: 16.5-29.7). Lower seroprevalence rates were observed in the Eastern Mediterranean (16.0%, 95% CI: 10.1-23.1), Western Pacific (15.6%, 95% CI: 8.2-24.9), Africa (8.4%, 95% CI: 4.8-12.9) and Europe (0.0%).

Comparing seroprevalences between two time periods (Jan 2020 to Apr 2015 and May 2015 to Jul 2023), the overall seroprevalence increased from 12.6% (95%CI 6.4-20.4%) to 25.7% (95%CI 20.5-31.3%). Evidence of ZIKV circulation during both periods was documented in Africa (5.1% vs 15.0%), Southeast Asia (23.3% vs 22.1%), and Western Pacific (16.1% vs 15.5%). Further details on heterogeneity among studies, seroprevalence rates across WHO regions, countries, and time periods are available in Table 1 and 2, S4 and S5 Table.

Table 2: Subgroup analysis of Zika virus seroprevalence per World Health Organization region.

Characteristics	Worldwide Prevalence		Africa Prevalence		America Prevalence		Eastern Mediterranean Prevalence		Southeast Asia Prevalence		Western Pacific Prevalence	
	%	95%CI	%	95%CI	%	95%CI	%	95%CI	%	95%CI	%	95%CI
**Population type**												
General	23.4	17.2-30.2	16.1	7.0-27.8	42.8	32.4-53.4	12.0[a]	6.5-18.9	28.7	14.9-44.9	15.2	6.9-25.9
Pregnant women	32.2	21.0-44.5	11.9	5.5-20.4	62.3	44.4-78.5	18.5[a]	14.9-22.5	25.1	21.4-28.9	1.0	0.4-1.8
Blood donors	8.2	3.2-17.7	6.1	1.3-14.0	10.4	3.5-20.3	NA	NA	NA	NA	2.9	0.6-6.6
**Age**												
Children	14.3	6.9-23.8	4.9	0.05-17.1	27.3	20.9-34.1	NA	NA	9.2	7.9-10.8	16.4	13.4-19.8
Adults	20.6	15.2-26.6	8.4	4.7-5.5	39.8	32.0-47.9	18.5[a]	14.9-22.5	24.1	20.3-28.2	9.2	3.2-17.8
Children and Adults[b]	15.5	7.0-25.7	6.3	5.6-6.9	37.6	8.9-72.3	3.5	2.0-24.6	7.7	0.5-35.7	25.3	9.6-45.4
**Settings**												
Urban	19.7	13.8-26.4	11.1	5.3-18.7	38.0	27.7-49.0	17.0	13.9-20.4	23.3	12.4-36.4	4.3	1.3-8.8
Rural	14.1	7.3-22.7	7.2	3.1-12.7	28.6	6.9-57.5	12.0[a]	6.5-18.9	NA	NA	9.1	2.9-18.3
Semi-urban	31.0	14.9-50.0	43.7[a]	33.7-53.9	NA	NA	NA	NA	NA	NA	17.5	15.0-20.2
**Serological methods**												
ELISA	30.6	22.9-38.9	19.5	9.4-32.0	43.2	27.6-59.4	16.0	10.1-23.1	26.8	17.2-37.7	34.0	12.4-59.8
ELISA & PRNT or FRNT	14.7	3.6-31.4	0.1	0.0-0.2	39.5	10.6-73.3	NA	NA	30.8[a]	27.3-34.4	6.6	0.4-19.0
ELISA & VNT	15.8	9.5-23.4	6.6	3.2-11.1	35.0	22.8-48.2	NA	NA	NA	NA	4.8	1.5-9.6
PRNT90	12.9	5.7-22.5	3.9[a]	2.7-5.3	NA	NA	NA	NA	15.9	8.0-25.8	0.0[a]	NA
MIA	20.8	9.1-35.7	NA	NA	NA	NA	NA	NA	NA	NA	20.8	9.1-35.7
Other methods	22.1	10.8-36.1	16.1	9.2-24.2	39.6	28.5-51.2	NA	NA	17.0[a]	11.3-23.5	8.7[a]	0.0-43.2
**ZIKV strain**												
Asian	24.9	17.9-32.6	4.1	0.9-9.5	44.9	34.9-55.2	NA	NA	13.1	8.0-19.1	17.0	8.5-27.6
African	9.1	3.1-17.6	6.1	2.1-11.8	NA	NA	NA	NA	26.7[a]	24.5-28.9	2.5	0.0-94.6

[a]Less than two studies, [b]No individual data. Europe: No seropositivity

Abbreviations: ELISA: Enzyme-linked immunosorbent assay; FRNT: Focus reduction neutralization assay; MIA: Microsphere immunoassay; NA: No data available; PRNT: Plaque reduction neutralization test; VNT: Virus neutralization test.

The top five countries with the highest seroprevalence rates were Honduras (73.8%, 95%CI 69.9-77.6%), Papua New Guinea (64.9%, 95%CI 58.3-71.3%), Mexico (60.1%, 95%CI 56.9-63.2%), Nicaragua (57.0%, 95%CI 37.8-75.1%), and Gabon (43.7%, 95%CI 33.7-53.9%). Conversely, the countries with the lowest ZIKV seroprevalence rates were Madagascar, Iran, and European countries (0.0%). Fig 2B displays seroprevalence rates by country.

Before May 2015, provinces like Moyen-Ogooué in Gabon, Ratchaburi in Thailand (~55.0%), and the Society Islands in French Polynesia (40.0%), exhibited high seropositivity, determined through ELISA tests. After 2015, high seroprevalences rates were observed in various regions. In Africa: Moyen-Ogooué province in Gabon (maintained a high seroprevalence 41.6%), followed by Gambella, Ethiopia (27.3%, BOB ELISA, African strain), and Ouagadougou and Bobo-Dioulasso, Burkina Faso (22.8%, cELISA & VNT, African strain) [8]. In the Americas: Departments of Córdoba (89.0%, ELISA) and Risaralda (86.8%, ELISA & FRNT) in Colombia, Acapulco in Mexico, and Tegucigalpa in Honduras (85.0%, 73.8%, respectively; BOB ELISA and VNT). In the Eastern Mediterranean: The cities of Najran (18.5%) in Saudi Arabia, and Basrah in Iraq (12.0%) both determined using ELISA tests. In Southeast Asia: Samut Prakan province in Thailand (45.4%, ELISA). In the Western Pacific region: Manus and Wewak Islands (~65.0%) in Papua New Guinea (ELISA and VNT, African strain), North Guadalcanal and Honiara (~55%) in the Solomon Islands (ELISA), and the Society Islands in French Polynesia (42.1%, similar seroprevalence rates as before 2015).

The lowest seroprevalence rates after the emergence of ZIKV in the Americas were found in the highlands of Bolivia (La Paz and Cochabamba, 0.0%) [1], the subtropics of Brazil (Santa Maria, Rio Grande do Sul, 0.6%), China (Guizhou province, 0.0%), and Taiwan (Tainan, 0.5%), and in the tropical rainforest of Indonesia (Aceh province, 0.0%). The seroprevalence rates by administrative division and by sampling period are shown in Fig 3A and 3B.

Fig 3. (A) Seroprevalence of Zika virus according to administrative divisions from January 2000 to April 2015. (B) Seroprevalence of Zika virus according to administrative divisions from May 2015 to July 2023.

The maps were created using MapChart (https://www.mapchart.net/)

In subgroup analysis, the seroprevalence was estimated to be higher in pregnant women (32.2%, 95%CI 21.0-44.5%), than in the general population (23.4%, 95%CI 17.2-30.2) and in blood donors (8.2%, 95%CI 3.2-17.7) ($p<0.001$). Globally, adults were significantly more exposed to ZIKV compared to exclusively children (20.6% vs 14.3%, p-value <0.001), with particularly low seroprevalence in children in Africa (4.9%) and Southeast Asia (9.2%). Seroprevalence was higher in semiurban (31.0%, 14.9-50.0%) and urban (19.7%, 13.8-26.4%) compared to rural areas (14.1%, 7.3-22.7%). Moreover, increased sensitivity of ELISA tests (30.6%, 22.9-38.9%) was significantly associated with increased seroprevalence compared to the combination of ELISA and a seroneutralization tests (PRNT or FRNT 14.7%, 3.6-31.4%, or VNT 15.8%, 9.5-23.4%, p-value <0.001). Using the Asian lineage (24.9%, 17.9-32.6%), resulted in higher seroprevalence compared to the African lineage (9.1%, 3.1-17.6%) (p-value <0.001). Additionally, other factors

contributing to seropositivity in specific studies included low educational levels, low socio-economic status, and polygamy.

Discussion

Zika virus is a mosquito-borne illness that has affected thousands of people in tropical and subtropical regions worldwide and has caused severe birth effects. In this systematic review and meta-analysis, we retrieved information on the prevalence of ZIKV infection from January 2000 to July 2023 and analyzed it at global, regional, and national levels. Our study aimed to understand and provide a more comprehensive perspective of global ZIKV infection, identifying trends over time, and addressing future challenges.

The estimated pooled global seroprevalence was 21.0% by involving 84 studies and 63,864 individuals. The findings indicate a high heterogeneity of study designs and serological tests. Similar seroprevalence was identified in a previous systematic review analyzing 12 studies until 2019 [6] and indicate a progressive growing of ZIKV seroprevalence studies in the last four years.

The assessment of seroprevalence involved a variety of diagnostic methods, resulting in challenges when determining the relative accuracy, sensitivity, and specificity, as well as when estimating the performance value of each study through proper comparison. The most commonly used method, the indirect semi-quantitative NS1 ELISA IgG, was preferred due to its higher sensitivity, stronger response, longer duration of detectability, and reduced cross-reactivity compared to the envelope protein E, which is highly conserved and leads to suboptimal diagnoses [58]. However, significant cross-reactivity persists, as demonstrated in a study in Brazil with 54.0% of cross-reactivity when compared with PRNT [59], and 60.0% in a study in Bolivia compared with VNT [1]. Nonetheless, significant advancements and investments have been made in the development and improvement of tests. For instance, the use of the WHO International

Standard (WHO ISs) for anti-Asian linage ZIKV antibody (IS 16_352) allows for the comparison of biological activity by defining an internationally agreed unit, the International Unit (IU27), using a calibrator for serology assays. This standardization facilitates the harmonization of data from different laboratories worldwide and provides better definition of parameters such as the analytical sensitivity of tests and protective levels of antibody [60]. Other promising tests such as the ZIKV NS1 BOB ELISA, a simple, robust, and cost-effective test, have been used in some seroprevalence studies. In this assay, serum antibodies are measured for their ability to block the binding of a ZIKV NS1- specific monoclonal antibody to solid-phase ZIKV NS1. This method has shown higher specificity compared to other ELISAs, but should undergo further validation [41]. Additionally, some studies have used the envelope protein domain III (EDIII), which is highly diverse among flaviviruses and can improve sensitivity and specificity of the assays [61]. Nevertheless, neutralization tests continue to be considered the "gold standard" and provide better specificity, but the potential for cross-reactivity persists in flavivirus-endemic areas. Variability across neutralization tests, including the type of assay, target cells, virus stock, timings, and titer threshold, has been observed. According to the WHO guidelines for recent ZIKV infection, a PRNT using a 90% cutoff value with a titer ≥10 and negativity for other flaviviruses is confirmatory [62]. However, no information regarding the titer in seroprevalence studies is available. The implementation of standardized protocols is essential, as they will not only enable the comparison of results across different regions and countries but also enhance the quality of studies by reducing potential biases.

In Africa, ZIKV has been circulating since 1945 [2], primarily the African genotype. However, large-scale outbreaks have been relatively infrequent, as shown in our study, with an overall seroprevalence of 8.4%, which increased from 5.1% before May 2015 to 15.0% after May

2015. According to the WHO, ZIKV transmission has been reported in 14 African countries or territories, including those where seroprevalence studies were conducted, such as Burkina Faso (with a seroprevalence of 22.8%), Cabo Verde (10.9%), Cameroon (4.9%), Ethiopia (27.3%), Gabon (43.7%), Kenya (1.5%), Nigeria (13.1%), and Senegal (10.1%) [63]. The high seroprevalence found in Gabon is attributable to the largest epidemic ever recorded in Africa, which occurred in 2007. This epidemic was potentially caused by *Ae. albopictus*, which is present in all human environments in Gabon and is thought to be more susceptible to African ZIKV [64] compared to African *Ae. aegypti*, which is more resistant to American and Asian ZIKV. However, an exception is the emergence of the Asian lineage in Cabo Verde (7,580 suspected cases) in 2015-2016 caused by *Ae. aegypti* [65]. Studies indicate that *Ae. aegypti* in Cabo Verde appear to have a strong attraction to human blood and a high susceptibility to ZIKV infection compared to generalist ancestral subspecies *Ae. aegypti formosus* (Aaf), which predominates in Africa. This genomic profile was also identified in Senegal, which had a similar seroprevalence between 2007 and 2012, and in Angola, where only four acute cases were detected in 2016-2017 [66]. Moreover, the moderate seroprevalence found in Ethiopia is associated with individuals who visited forest areas [12]. As for Burkina Faso, further studies are needed to understand and potentially identify any undetected epidemics [8]. There is a high public health concern regarding the possibility of African ZIKV triggering large urban epidemics, along with the risk associated with *Ae. albopictus*. Experiments have demonstrated that African strains are more pathogenic than Asian strains, and that *Ae. aegypti* mosquitoes are highly competent to transmit the different genotypes, specially, the African [64]. Hence, there is a need to enhance vector surveillance and control methods in the region to reduce the risk of potential future outbreaks.

In the Americas, autochthonous vector-borne transmission of ZIKV has been confirmed in all countries and territories, except for continental Bermuda, Canada, mainland Chile, and Uruguay. Regarding the number of cases reported between 2015 and 2023, Brazil (4th in incidence), Colombia and Venezuela top the list with 508,609, 111,744 and 62,093 cases, respectively [67]. Our meta-analysis revealed a seroprevalence of 39.9% within the region, which ascends to ~50% in many tropical regions, reflecting the substantial degree of exposure to ZIKV among the population, particularly in Honduras, Mexico, and Colombia. However, comparing the number of reported cases by country and their seroprevalence presents challenges. On one hand, there are disparities in case reporting, such as confirmed cases (e.g. Mexico) versus probable and confirmed cases (e.g. Brazil, Colombia). In addition to variations in diagnostic protocols and laboratory capacity. On the other hand, seroprevalence is influenced by variations in methods, study periods, the number of studies, and study regions. Consequently, these limitations may affect the generalizability of the findings.

Currently, there is a growing concern in the region due to the recent confirmation of the presence of the *Ae. aegypti* in the highlands of Bolivia (Cochabamba, 2,558 m), the expansion of the vector's distribution in new regions of Chile (e.g. Parinacota and Los Andes in Valparaíso Region), the identification of local Chikungunya transmission in Uruguay in 2023 [68], and a notable increase in arbovirus activity in the state of Rio Grande do Sul, Southern Brazil [69]. These expansions are attributable to climate change, heightening the potential for future transmission in previously unaffected areas. Additional risks involve the recent identification of the African lineage in Brazil among non-human primates and mosquitoes in geographically and climatically distinct regions (South and Southeast), posing a potential threat associated with birth defects [70]. Therefore,

further research is essential to better understand the prevalence of ZIKV, vector dynamics, and the genetic evolution of the virus in the Americas.

In the Eastern Mediterranean Region (EMR), no country has reported autochthonous transmission of ZIKV. Nevertheless, documented records indicate the presence of *Ae. aegypti* populations in Afghanistan, Djibouti, Egypt, Oman, Pakistan, Saudi Arabia, Somalia, Sudan and Yemen [4]. Seroprevalence studies have revealed the presence of IgG antibodies in two countries within their population. First, in the city of Najran in southwestern Saudi Arabia, with a seroprevalence of 18.5% between 2016-2017. This region experiences a growing annual increase in dengue cases [71]. Second, in the city of Basra, southern Iraq, with a seroprevalence of 12.0% in 2019-2020. This country is free of transmission and presence of known vectors. However, these studies were performed using ELISA tests. Consequently, it is challenging to discern whether this seropositivity is the result of possible cross-reaction with other flaviviruses or actual ZIKV transmission. A study has demonstrated that all EMR countries are suitable for *Ae. aegypti* and *Ae. albopictus*. Thus, it is imperative to undertake active entomological and epidemiological surveillance throughout the region to help identify the introduction of mosquito species and the circulation of arboviruses [72].

In Europe, Southern France (Hyeres, Var department) was the first and only region reporting three autochthonous transmission in October 2019. Subsequently, a seroprevalence study was undertaken in the same area, but it yielded no positive cases [42]. This localized transmission event was attributed to *Ae. albopictus*, a mosquito species well-established in France since 2004, potentially by the Asian genotype originating from Southeast Asia [73]. Previous studies revealed the reduced susceptibility of the French *Ae. albopictus* populations (Montpellier and Corsica) to the Asian ZIKV compared to the African ZIKV genotype. Moreover, these mosquitoes

demonstrated limited capacity to sustain local virus transmission [74]. It is plausible that environmental factors played a role in this brief episode of local transmission, despite the apparent limitations in vector competence [73]. To gain a deeper understanding of the potential for ZIKV to established a sustained transmission cycle involving *Ae. albopictus* in southern Europe, further investigations are needed. This is especially crucial in light of the evolving epidemiological situation of Dengue virus in France, which has seen a rising number of autochthonous cases being reported recently [75].

In Southeast Asia, ZIKV has been circulating since at least the 1950s. Surprisingly, ZIKV outbreaks in this region have remained moderate in intensity, with notable outbreaks occurring in Thailand in 2016-2018 (n=2,300), and in India in 2018 (n=283 cases) [2,76]. In our study, the estimated seroprevalence was determined to be 25.9% (Thailand 25.9%, Indonesia 9.1% in children), with evidence of prior circulation before May 2015. Studies have hypothesized that the observed differences in ZIKV outbreaks patterns between Southeast Asia and the Americas may be attributed to the fact that, despite the highly efficient *Ae. aegypti* mosquitoes in Southeast Asia, the transmission efficiency of Asian ZIKV strains may not be as robust as that of American strains [77], and/or to the acquisition of genetic changes that increased ZIKV infectivity and prevalence in mosquitoes of the Americas [78]. Other studies also indicate that in regions with endemic transmission, such as Thailand, there is an increase in genetic diversity among circulating lineages that would limit sustained transmission chains [79]. To gain deeper insights into the genetic diversity and the persistent circulation in the region, it is imperative to conduct extensive surveillance in competent mosquito vectors across Southeast Asia. Moreover, Thailand's laboratory capabilities should be shared to improve the capacities of neighboring countries in the region.

In the Western Pacific region, large epidemics, likely caused by the American sublineage, were reported in French Polynesia between 2013 and 2014, followed by outbreaks in the South Pacific Islands [3]. Correspondingly, studies showed higher seroprevalence in Fiji (13.7%), French Polynesia (40.7%), Papua New Guinea (64.9%), and Solomon Islands (28.1%). Conversely, sporadic cases or small outbreaks, probably caused by the Asian sublineage [3], were identified in countries where seroprevalence studies were conducted, such as Lao PDR (7.0%), Malaysia (7.1%), The Philippines (17.9%), and Vietnam (1.0%) [2,80]. Consequently, Malaysia, Laos and Vietnam may have lower levels of immunity, making them highly vulnerability to future epidemics. Moreover, evidence indicating a decline in seroprevalence over a two-year period reported in French Polynesia [46] underscores the need for exhaustive monitoring of ZIKV.

In subgroup analysis, higher seroprevalence was identified in: a) Pregnant women, possibly because some studies were conducted during epidemics and potentially involved a higher participation of pregnant women with previous symptoms, which might introduce biases into the results. b) Adults, known to result from greater exposure to outdoor activities. c) Urban and semi-urban areas, linked to the urban maintenance cycles of ZIKV in recent large epidemics. Additionally, some studies suggest an association with low educational and low-socio-economic levels, likely resulting from poor-quality housing conditions, overcrowding, and the storage of water in containers that create breeding sites for mosquitoes. A study has also indicated an association with polygamous relationships, suggesting a possible transmission through sexual contact.

Our meta-analysis faced several limitations. Our literature search highlights the highly heterogeneous seroprevalence of ZIKV worldwide. Some of these variations may stem from methodological differences, as well as the choice of study population, and sample size. This

heterogeneity may also reflect differential exposure to mosquitoes and the period of study—before, during, or after the end of the outbreak. Other limitations include the differences in the number of studies per country, which may increase or decrease the seroprevalence. Additionally, our study was based only in the detection of IgG or IgM/IgG. Although this may represent a major limitation for the comparison of seroprevalence, this study has strengths. A comprehensive search strategy was performed, with almost 80.0% of the studies assessed as having low risk of bias in their methodological quality. This suggests that we can be confident in the quality of our findings and also provides an updated global image of the Zika situation in the world until now.

In conclusion, ZIKV infection remains a public health threat. As ongoing social, environmental and economic changes continue to alter our environment, arboviral importation and transmission will persist. Addressing remaining gaps in its distribution requires the implementation of standardized seroprevalence studies, including the development of more sensitive and specific diagnostic tools.

References

1. Saba Villarroel PM, Nurtop E, Pastorino B, Roca Y, Drexler JF, Gallian P, et al. Zika virus epidemiology in Bolivia: A seroprevalence study in volunteer blood donors. PLoS Negl Trop Dis. 2018;12: e0006239. doi:10.1371/journal.pntd.0006239
2. Saba Villarroel PM, Gumpangseth N, Songhong T, Yainoy S, Monteil A, Leaungwutiwong P, et al. Emerging and re-emerging zoonotic viral diseases in Southeast Asia: One Health challenge. Frontiers in Public Health. 2023;11. Available: https://www.frontiersin.org/articles/10.3389/fpubh.2023.1141483

3. Noisumdaeng P, Dangsagul W, Sangsiriwut K, Prasertsopon J, Changsom D, Yoksan S, et al. Molecular characterization and geographical distribution of Zika virus worldwide from 1947 to 2022. International Journal of Infectious Diseases. 2023;136: 5–10. doi:10.1016/j.ijid.2023.08.023

4. Zika epidemiology update - February 2022. [cited 13 Sep 2023]. Available: https://www.who.int/publications/m/item/zika-epidemiology-update---february-2022

5. Hamel R, Liégeois F, Wichit S, Pompon J, Diop F, Talignani L, et al. Zika virus: epidemiology, clinical features and host-virus interactions. Microbes and Infection. 2016;18: 441–449. doi:10.1016/j.micinf.2016.03.009

6. Li Z, Wang J, Cheng X, Hu H, Guo C, Huang J, et al. The worldwide seroprevalence of DENV, CHIKV and ZIKV infection: A systematic review and meta-analysis. PLoS Negl Trop Dis. 2021;15: e0009337. doi:10.1371/journal.pntd.0009337

7. Assessing risk of bias in prevalence studies: modification of an existing tool and evidence of interrater agreement - PubMed. [cited 15 Sep 2023]. Available: https://pubmed.ncbi.nlm.nih.gov/22742910/

8. Tinto B, Kaboré DPA, Kania D, Kagoné TS, Kiba-Koumaré A, Pinceloup L, et al. Serological Evidence of Zika Virus Circulation in Burkina Faso. Pathogens. 2022;11: 741. doi:10.3390/pathogens11070741

9. Ward D, Gomes AR, Tetteh KKA, Sepúlveda N, Gomez LF, Campino S, et al. Sero-epidemiological study of arbovirus infection following the 2015–2016 Zika virus outbreak in Cabo Verde. Sci Rep. 2022;12: 11719. doi:10.1038/s41598-022-16115-4

10. Gake B, Vernet MA, Leparc-Goffart I, Drexler JF, Gould EA, Gallian P, et al. Low seroprevalence of Zika virus in Cameroonian blood donors. Braz J Infect Dis. 2017;21: 481–483. doi:10.1016/j.bjid.2017.03.018
11. Willcox AC, Collins MH, Jadi R, Keeler C, Parr JB, Mumba D, et al. Seroepidemiology of Dengue, Zika, and Yellow Fever Viruses among Children in the Democratic Republic of the Congo. Am J Trop Med Hyg. 2018;99: 756–763. doi:10.4269/ajtmh.18-0156
12. Asebe G, Michlmayr D, Mamo G, Abegaz WE, Endale A, Medhin G, et al. Seroprevalence of Yellow fever, Chikungunya, and Zika virus at a community level in the Gambella Region, South West Ethiopia. PLoS ONE. 2021;16. doi:10.1371/journal.pone.0253953
13. Ushijima Y, Abe H, Nguema Ondo G, Bikangui R, Massinga Loembé M, Zadeh VR, et al. Surveillance of the major pathogenic arboviruses of public health concern in Gabon, Central Africa: increased risk of West Nile virus and dengue virus infections. BMC Infect Dis. 2021;21: 265. doi:10.1186/s12879-021-05960-9
14. Sherman KE, Rouster SD, Kong LX, Shata TM, Archampong T, Kwara A, et al. Zika Virus Exposure in an HIV-Infected Cohort in Ghana. J Acquir Immune Defic Syndr. 2018;78: e35–e38. doi:10.1097/QAI.0000000000001718
15. Kisuya B, Masika MM, Bahizire E, Oyugi JO. Seroprevalence of Zika virus in selected regions in Kenya. Transactions of The Royal Society of Tropical Medicine and Hygiene. 2019;113: 735–739. doi:10.1093/trstmh/trz077
16. Chepkorir E, Tchouassi DP, Konongoi SL, Lutomiah J, Tigoi C, Irura Z, et al. Serological evidence of Flavivirus circulation in human populations in Northern Kenya: an assessment of disease risk 2016-2017. Virol J. 2019;16: 65. doi:10.1186/s12985-019-1176-y

17. Schwarz NG, Mertens E, Winter D, Maiga-Ascofaré O, Dekker D, Jansen S, et al. No serological evidence for Zika virus infection and low specificity for anti-Zika virus ELISA in malaria positive individuals among pregnant women from Madagascar in 2010. PLoS One. 2017;12: e0176708. doi:10.1371/journal.pone.0176708

18. Marchi S, Viviani S, Montomoli E, Tang Y, Boccuto A, Vicenti I, et al. Zika Virus in West Africa: A Seroepidemiological Study between 2007 and 2012. Viruses. 2020;12: 641. doi:10.3390/v12060641

19. Diarra I, Nurtop E, Sangaré AK, Sagara I, Pastorino B, Sacko S, et al. Zika Virus Circulation in Mali. Emerg Infect Dis. 2020;26: 945–952. doi:10.3201/eid2605.191383

20. Mathé P, Egah DZ, Müller JA, Shehu NY, Obishakin ET, Shwe DD, et al. Low Zika virus seroprevalence among pregnant women in North Central Nigeria, 2016. Journal of Clinical Virology. 2018;105: 35–40. doi:10.1016/j.jcv.2018.05.011

21. Kolawole OM, Suleiman MM, Bamidele EP. Molecular epidemiology of Zika virus and Rubella virus in pregnant women attending Sobi Specialist Hospital Ilorin, Nigeria. International Journal of Research in Medical Sciences. 2020;8: 2275–2283. doi:10.18203/2320-6012.ijrms20202234

22. Shaibu JO, Okwuraiwe AP, Jakkari A, Dennis A, Akinyemi KO, Li J, et al. Sero-molecular Prevalence of Zika Virus among Pregnant Women Attending Some Public Hospitals in Lagos State, Nigeria. European Journal of Medical and Health Sciences. 2021;3: 77–82. doi:10.24018/ejmed.2021.3.5.1075

23. Anejo-Okopi J, Gotom DY, Chiehiura NA, Okojokwu JO, Amanyi DO, Egbere JO, et al. The Seroprevalence of Zika Virus Infection among HIV Positive and HIV Negative Pregnant

Women in Jos, Nigeria. Hosts and Viruses. 637134336000000000;7. doi:10.17582/journal.hv/2020/7.6.129.136

24. Mac PA, Kroeger A, Daehne T, Anyaike C, Velayudhan R, Panning M. Zika, Flavivirus and Malaria Antibody Cocirculation in Nigeria. Tropical Medicine and Infectious Disease. 2023;8: 171. doi:10.3390/tropicalmed8030171

25. Nurtop E, Moyen N, Dzia-Lepfoundzou A, Dimi Y, Ninove L, Drexler JF, et al. A Report of Zika Virus Seroprevalence in Republic of the Congo. Vector Borne Zoonotic Dis. 2020;20: 40–42. doi:10.1089/vbz.2019.2466

26. Seruyange E, Gahutu J-B, Muvunyi CM, Katare S, Ndahindwa V, Sibomana H, et al. Seroprevalence of Zika virus and Rubella virus IgG among blood donors in Rwanda and in Sweden. J Med Virol. 2018;90: 1290–1296. doi:10.1002/jmv.25198

27. Soghaier MA, Abdelgadir DM, Abdelkhalig SM, Kafi H, Zarroug IMA, Sall AA, et al. Evidence of pre-existing active Zika virus circulation in Sudan prior to 2012. BMC Res Notes. 2018;11: 906. doi:10.1186/s13104-018-4027-9

28. Mwanyika GO, Sindato C, Rugarabamu S, Rumisha SF, Karimuribo ED, Misinzo G, et al. Seroprevalence and associated risk factors of chikungunya, dengue, and Zika in eight districts in Tanzania. Int J Infect Dis. 2021;111: 271–280. doi:10.1016/j.ijid.2021.08.040

29. Chisenga CC, Bosomprah S, Musukuma K, Mubanga C, Chilyabanyama ON, Velu RM, et al. Sero-prevalence of arthropod-borne viral infections among Lukanga swamp residents in Zambia. PLoS One. 2020;15: e0235322. doi:10.1371/journal.pone.0235322

30. Babaniyi OA, Mwaba P, Songolo P, Mazaba-Liwewe ML, MweeneNdumba I, Masaninga F, et al. Seroprevalence of Zika virus infection specific IgG in Western and North-Western Provinces of Zambia. International Journal of Public Health and Epidemiology. 2014;4: 1–6.

31. Lamb MM, Paniagua-Avila A, Zacarias A, Rojop N, Chacon A, Natrajan MS, et al. Repeated Rapid Active Sampling Surveys Demonstrated a Rapidly Changing Zika Seroprevalence among Children in a Rural Dengue-endemic Region in Southwest Guatemala during the Zika Epidemic (2015-2016). Am J Trop Med Hyg. 2022;107: 1099–1106. doi:10.4269/ajtmh.22-0399

32. Rivas E, Ojeda J, Garcia-Rivera EJ, Rivera DM, Arredondo JL, Medina EL, et al. Prospective surveillance of Zika virus at the end of the Americas' outbreak: An unexpected outcome. Frontiers in Tropical Diseases. 2022;3. Available: https://www.frontiersin.org/articles/10.3389/fitd.2022.1027908

33. Anzinger JJ, Mears CD, Ades AE, Francis K, Phillips Y, Leys YE, et al. Antenatal Seroprevalence of Zika and Chikungunya Viruses, Kingston Metropolitan Area, Jamaica, 2017-2019. Emerg Infect Dis. 2022;28: 473–475. doi:10.3201/eid2802.211849

34. Gallian P, Cabié A, Richard P, Paturel L, Charrel RN, Pastorino B, et al. Zika virus in asymptomatic blood donors in Martinique. Blood. 2017;129: 263–266. doi:10.1182/blood-2016-09-737981

35. Eligio-García L, Crisóstomo-Vázquez M del P, Caballero-García M de L, Soria-Guerrero M, Méndez–Galván JF, López-Cancino SA, et al. Co-infection of Dengue, Zika and Chikungunya in a group of pregnant women from Tuxtla Gutiérrez, Chiapas: Preliminary data. 2019. PLOS Neglected Tropical Diseases. 2020;14: e0008880. doi:10.1371/journal.pntd.0008880

36. Cachay R, Schwalb A, Acevedo-Rodriguez JG, Merino X, Talledo M, Suarez-Ognio L, et al. Zika Virus Seroprevalence in Two Districts of Chincha, Ica, Peru: A Cross-Sectional Study. Am J Trop Med Hyg. 2021;106: 192–198. doi:10.4269/ajtmh.20-1339

37. Langerak T, Brinkman T, Mumtaz N, Arron G, Hermelijn S, Baldewsingh G, et al. Zika Virus Seroprevalence in Urban and Rural Areas of Suriname, 2017. J Infect Dis. 2019;220: 28–31. doi:10.1093/infdis/jiz063

38. Ziyaeyan M, Behzadi MA, Leyva-Grado VH, Azizi K, Pouladfar G, Dorzaban H, et al. Widespread circulation of West Nile virus, but not Zika virus in southern Iran. PLoS Negl Trop Dis. 2018;12: e0007022. doi:10.1371/journal.pntd.0007022

39. ShantASinbat AKM. Seroepidemiology of Zikavirus in Basrah, Southern Iraq. Annals of the Romanian Society for Cell Biology. 2021;25: 12546–12553.

40. Alayed MS, Qureshi MA, Ahmed S, Alqahtani AS, Al-Qahtani AM, Alshaybari K, et al. Seroprevalence of Zika virus among asymptomatic pregnant mothers and their newborns in the Najran region of southwest Saudi Arabia. Ann Saudi Med. 2018;38: 408–412. doi:10.5144/0256-4947.2018.408

41. Abushoufa F, Arikan A, Sanlidag T, Guvenir M, Guler E, Suer K. Absence of Zika Virus Seroprevalence Among Blood Donors in Northern Cyprus. J Infect Dev Ctries. 2021;15: 1032–1034. doi:10.3855/jidc.12766

42. Franke F, Noël H, Durand GA, Giron S, Decoppet A, de Valk H, et al. 81 - Enquête de séroprévalence suite à la première transmission vectorielle du Zika en Europe. Revue d'Épidémiologie et de Santé Publique. 2022;70: S167–S168. doi:10.1016/j.respe.2022.06.108

43. Sun J, Su J, Jiao X, Zhou H, Zhang H, Wu D, et al. Community based serosurvey of naïve population indicate no local circulation of Zika virus in an hyper endemic area of China 2016. Journal of Infection. 2019;79: 61–74. doi:10.1016/j.jinf.2019.03.013

44. Li F, Zhou JZ, Zhou L, Fu SH, Tian ZZ, Wang Q, et al. Serological Survey of Zika Virus in Humans and Animals in Dejiang Prefecture, Guizhou Province, China. Biomed Environ Sci. 2019;32: 875–880. doi:10.3967/bes2019.108
45. Zhou C-M, Liu J-W, Qi R, Fang L-Z, Qin X-R, Han H-J, et al. Emergence of Zika virus infection in China. PLoS Negl Trop Dis. 2020;14: e0008300. doi:10.1371/journal.pntd.0008300
46. Henderson AD, Aubry M, Kama M, Vanhomwegen J, Teissier A, Mariteragi-Helle T, et al. Zika seroprevalence declines and neutralizing antibodies wane in adults following outbreaks in French Polynesia and Fiji. Elife. 2020;9: e48460. doi:10.7554/eLife.48460
47. Kama M, Aubry M, Naivalu T, Vanhomwegen J, Mariteragi-Helle T, Teissier A, et al. Sustained Low-Level Transmission of Zika and Chikungunya Viruses after Emergence in the Fiji Islands. Emerg Infect Dis. 2019;25: 1535–1538. doi:10.3201/eid2508.180524
48. Pastorino B, Sengvilaipaseuth O, Chanthongthip A, Vongsouvath M, Souksakhone C, Mayxay M, et al. Low Zika Virus Seroprevalence in Vientiane, Laos, 2003–2015. Am J Trop Med Hyg. 2019;100: 639–642. doi:10.4269/ajtmh.18-0439
49. Sam I-C, Montoya M, Chua CL, Chan YF, Pastor A, Harris E. Low seroprevalence rates of Zika virus in Kuala Lumpur, Malaysia. Trans R Soc Trop Med Hyg. 2019;113: 678–684. doi:10.1093/trstmh/trz056
50. Khoo H-Y, Lee H-Y, Khor C-S, Tan K-K, Bin Hassan MR, Wong CM, et al. Seroprevalence of Zika Virus among Forest Fringe Communities in Peninsular Malaysia and Sabah: General Population-Based Study. Am J Trop Med Hyg. 2022;107: 560–568. doi:10.4269/ajtmh.21-0988

51. Khor C-S, Mohd-Rahim N-F, Hassan H, Tan K-K, Zainal N, Teoh B-T, et al. Serological evidence of DENV, JEV, and ZIKV among the indigenous people (Orang Asli) of Peninsular Malaysia. J Med Virol. 2020;92: 956–962. doi:10.1002/jmv.25649
52. Grant R, Kizu J, Graham M, McCallum F, McPherson B, Auliff A, et al. Serological evidence of possible high levels of undetected transmission of Zika virus among Papua New Guinea military personnel, 2019. IJID Regions. 2022;4: 131–133. doi:10.1016/j.ijregi.2022.07.006
53. Russell TL, Horwood PF, Harrington H, Apairamo A, Kama NJ, Bobogare A, et al. Seroprevalence of dengue, Zika, chikungunya and Ross River viruses across the Solomon Islands. PLoS Negl Trop Dis. 2022;16: e0009848. doi:10.1371/journal.pntd.0009848
54. Chien Y-W, Ho T-C, Huang P-W, Ko N-Y, Ko W-C, Perng GC. Low seroprevalence of Zika virus infection among adults in Southern Taiwan. BMC Infect Dis. 2019;19: 884. doi:10.1186/s12879-019-4491-4
55. Adams C, Jadi R, Segovia-Chumbez B, Daag J, Ylade M, Medina FA, et al. Novel Assay to Measure Seroprevalence of Zika Virus in the Philippines. Emerg Infect Dis. 2021;27: 3073–3081. doi:10.3201/eid2712.211150
56. Chiu Y-C, Baud D, Fahmi A, Zumkehr B, Vouga M, Pomar L, et al. Absence of Zika virus among pregnant women in Vietnam in 2008. Tropical Diseases, Travel Medicine and Vaccines. 2023;9: 4. doi:10.1186/s40794-023-00189-7
57. Nguyen CT, Moi ML, Le TQM, Nguyen TTT, Vu TBH, Nguyen HT, et al. Prevalence of Zika virus neutralizing antibodies in healthy adults in Vietnam during and after the Zika virus epidemic season: a longitudinal population-based survey. BMC Infectious Diseases. 2020;20: 332. doi:10.1186/s12879-020-05042-2

58. Delfin-Riela T, Rossotti MA, Mattiuzzo G, Echaides C, González-Sapienza G. Nanobody-Based Blocking of Binding ELISA for the Detection of Anti-NS1 Zika-Virus-Specific Antibodies in Convalescent Patients. Tropical Medicine and Infectious Disease. 2023;8: 55. doi:10.3390/tropicalmed8010055
59. Slavov SN, Guaragna Machado RR, Ferreira AR, Soares CP, Araujo DB, Leal Oliveira DB, et al. Zika virus seroprevalence in blood donors from the Northeastern region of São Paulo State, Brazil, between 2015 and 2017. Journal of Infection. 2020;80: 111–115. doi:10.1016/j.jinf.2019.10.002
60. Mattiuzzo G, Knezevic I, Hassall M, Ashall J, Myhill S, Faulkner V, et al. Harmonization of Zika neutralization assays by using the WHO International Standard for anti-Zika virus antibody. npj Vaccines. 2019;4: 1–9. doi:10.1038/s41541-019-0135-3
61. Ndiaye O, Diagne CT, Abd El Wahed A, Dia F, Dia M, Faye A, et al. Use of Envelope Domain III Protein for the Detection of IgG Type Antibodies Specific to Zika Virus by Indirect ELISA. Diagnostics. 2023;13: 462. doi:10.3390/diagnostics13030462
62. Sharp TM. Dengue and Zika Virus Diagnostic Testing for Patients with a Clinically Compatible Illness and Risk for Infection with Both Viruses. MMWR Recomm Rep. 2019;68. doi:10.15585/mmwr.rr6801a1
63. Countries and territories with current or previous Zika virus transmission. [cited 26 Oct 2023]. Available: https://www.who.int/publications/m/item/countries-and-territories-with-current-or-previous-zika-virus-transmission
64. Obadia T, Gutierrez-Bugallo G, Duong V, Nuñez AI, Fernandes RS, Kamgang B, et al. Zika vector competence data reveals risks of outbreaks: the contribution of the European ZIKAlliance project. Nat Commun. 2022;13: 4490. doi:10.1038/s41467-022-32234-y

65. Faye O, de Lourdes Monteiro M, Vrancken B, Prot M, Lequime S, Diarra M, et al. Genomic Epidemiology of 2015–2016 Zika Virus Outbreak in Cape Verde. Emerg Infect Dis. 2020;26: 1084–1090. doi:10.3201/eid2606.190928
66. Rose NH, Dabo S, Leal S da V, Sylla M, Diagne CT, Faye O, et al. Enhanced mosquito vectorial capacity underlies the Cape Verde Zika epidemic. PLOS Biology. 2022;20: e3001864. doi:10.1371/journal.pbio.3001864
67. PAHO/WHO Data - ZIKA. [cited 23 Oct 2023]. Available: https://www3.paho.org/data/index.php/en/mnu-topics/zika-weekly-en/
68. Estallo EL, Sippy R, Robert MA, Ayala S, Barboza Pizard CJ, Pérez-Estigarribia PE, et al. Increasing arbovirus risk in Chile and neighboring countries in the Southern Cone of South America. Lancet Reg Health Am. 2023;23: 100542. doi:10.1016/j.lana.2023.100542
69. Rio Grande do Sul teve redução de casos prováveis de dengue, Zika e chikungunya em 2023. In: Ministério da Saúde [Internet]. [cited 26 Oct 2023]. Available: https://www.gov.br/saude/pt-br/assuntos/noticias-para-os-estados/rio-grande-do-sul/2023/maio/rio-grande-do-sul-teve-reducao-de-casos-provaveis-de-dengue-zika-e-chikungunya-em-2023
70. Kasprzykowski JI, Fukutani KF, Fabio H, Fukutani ER, Costa LC, Andrade BB, et al. A recursive sub-typing screening surveillance system detects the appearance of the ZIKV African lineage in Brazil: Is there a risk of a new epidemic? International Journal of Infectious Diseases. 2020;96: 579–581. doi:10.1016/j.ijid.2020.05.090
71. Altassan KK, Morin C, Shocket MS, Ebi K, Hess J. Dengue fever in Saudi Arabia: A review of environmental and population factors impacting emergence and spread. Travel Medicine and Infectious Disease. 2019;30: 46–53. doi:10.1016/j.tmaid.2019.04.006

72. Ducheyne E, Tran Minh NN, Haddad N, Bryssinckx W, Buliva E, Simard F, et al. Current and future distribution of Aedes aegypti and Aedes albopictus (Diptera: Culicidae) in WHO Eastern Mediterranean Region. International Journal of Health Geographics. 2018;17: 4. doi:10.1186/s12942-018-0125-0
73. Durand GA, Piorkowski G, Thirion L, Ninove L, Giron S, Zandotti C, et al. Vector-Borne Transmission of the Zika Virus Asian Genotype in Europe. Viruses. 2020;12: 296. doi:10.3390/v12030296
74. Vazeille M, Madec Y, Mousson L, Bellone R, Barré-Cardi H, Sousa CA, et al. Zika virus threshold determines transmission by European Aedes albopictus mosquitoes. Emerg Microbes Infect. 8: 1668–1678. doi:10.1080/22221751.2019.1689797
75. Cochet A, Calba C, Jourdain F, Grard G, Durand GA, Guinard A, et al. Autochthonous dengue in mainland France, 2022: geographical extension and incidence increase. Eurosurveillance. 2022;27: 2200818. doi:10.2807/1560-7917.ES.2022.27.44.2200818
76. Kato DM, Samaraweera DS. Zika virus in the South-East Asia Region.
77. Pompon J, Morales-Vargas R, Manuel M, Huat Tan C, Vial T, Hao Tan J, et al. A Zika virus from America is more efficiently transmitted than an Asian virus by Aedes aegypti mosquitoes from Asia. Sci Rep. 2017;7: 1215. doi:10.1038/s41598-017-01282-6
78. Azar SR, Weaver SC. Vector Competence: What Has Zika Virus Taught Us? Viruses. 2019;11: 867. doi:10.3390/v11090867
79. Ruchusatsawat K, Wongjaroen P, Posanacharoen A, Rodriguez-Barraquer I, Sangkitporn S, Cummings DAT, et al. Observational study into the long-term circulation of Zika in Thailand. Lancet Infect Dis. 2019;19: 439–446. doi:10.1016/S1473-3099(18)30718-7

80. Biggs JR, Sy AK, Brady OJ, Kucharski AJ, Funk S, Tu Y-H, et al. Serological Evidence of Widespread Zika Transmission across the Philippines. Viruses. 2021;13: 1441. doi:10.3390/v13081441

Supporting information Captions

S1 Table. Prisma checklist.

S2 Table. Characteristics of Zika virus seroprevalence studies included in the systematic review and meta-analysis.

S3 Table: Risk of bias assessment

S4 Table: Heterogeneity assessment of seroprevalence of Zika virus per World Health Organization region and per study period.

S5 Table: Heterogeneity assessment of subgroup analysis of Zika virus seroprevalence per World Health Organization region.

S1 Figure. The global Zika virus seroprevalence using a random-effects model.

Funding sources/sponsors

This work was financially supported by the National Research Council of Thailand (NRCT): NRCT5-RGJ63012-125, Grant No. RGNS 64-172 by Office of the Permanent Secretary, Ministry of Higher Education, Science, Research and Innovation (MHESI) and international postdoctoral fellowship 2022 provided by Mahidol University.

Fig 1

Click here to access/download;Figure;Fig_1.tif

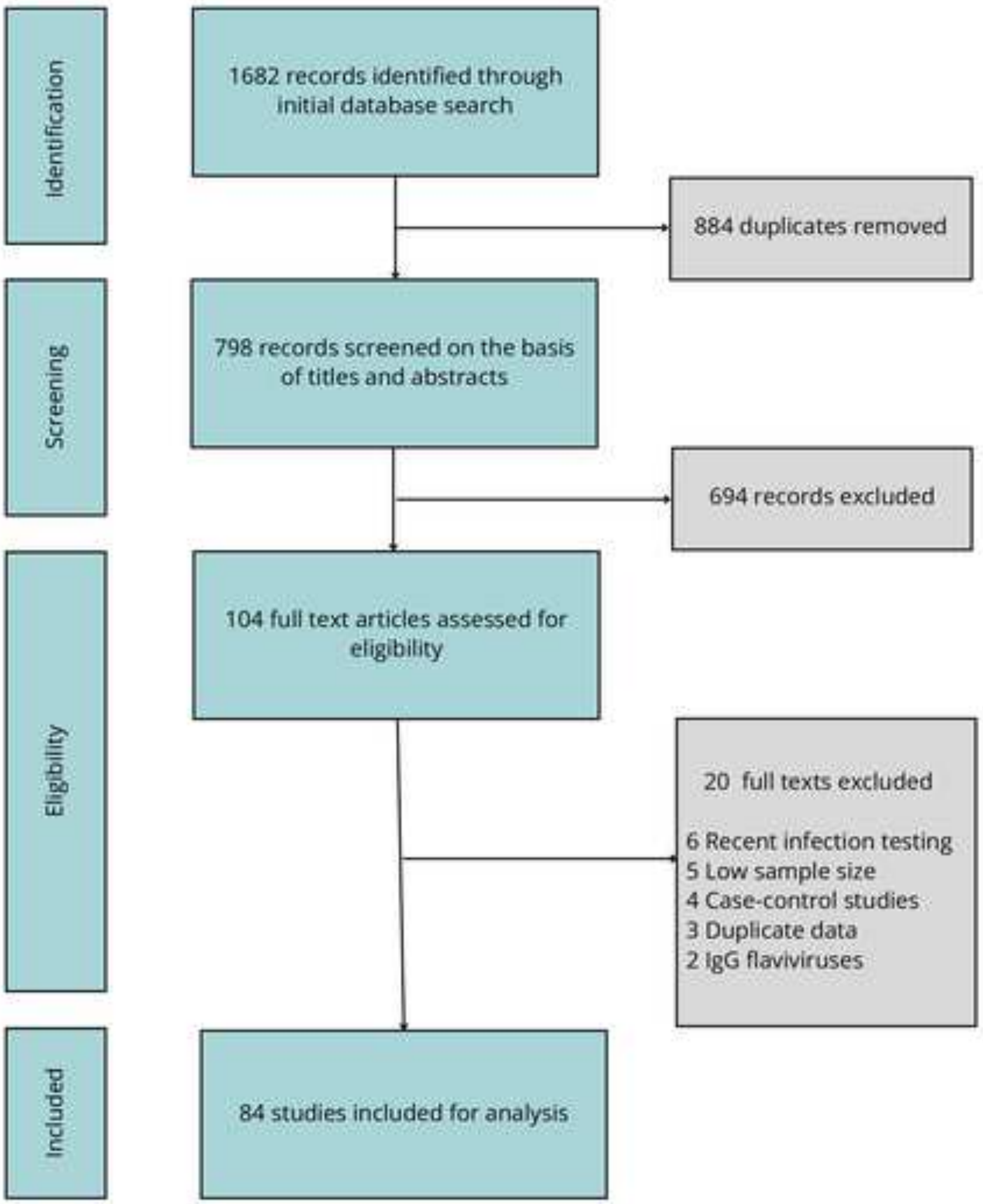

Fig 2

Click here to access/download;Figure;Fig_2.tif

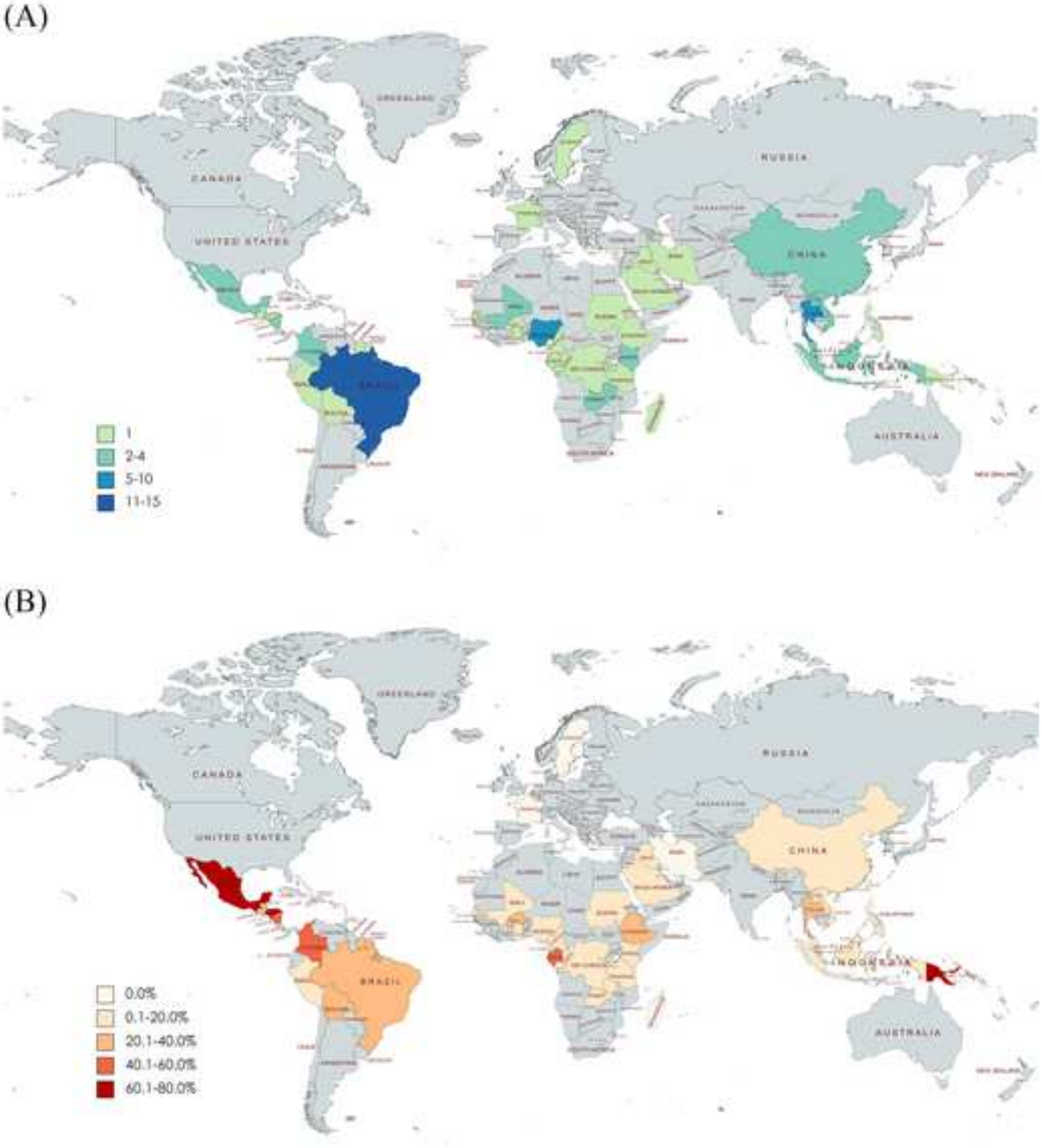

Fig 3

Click here to access/download;Figure;Fig_3.tif

(A)

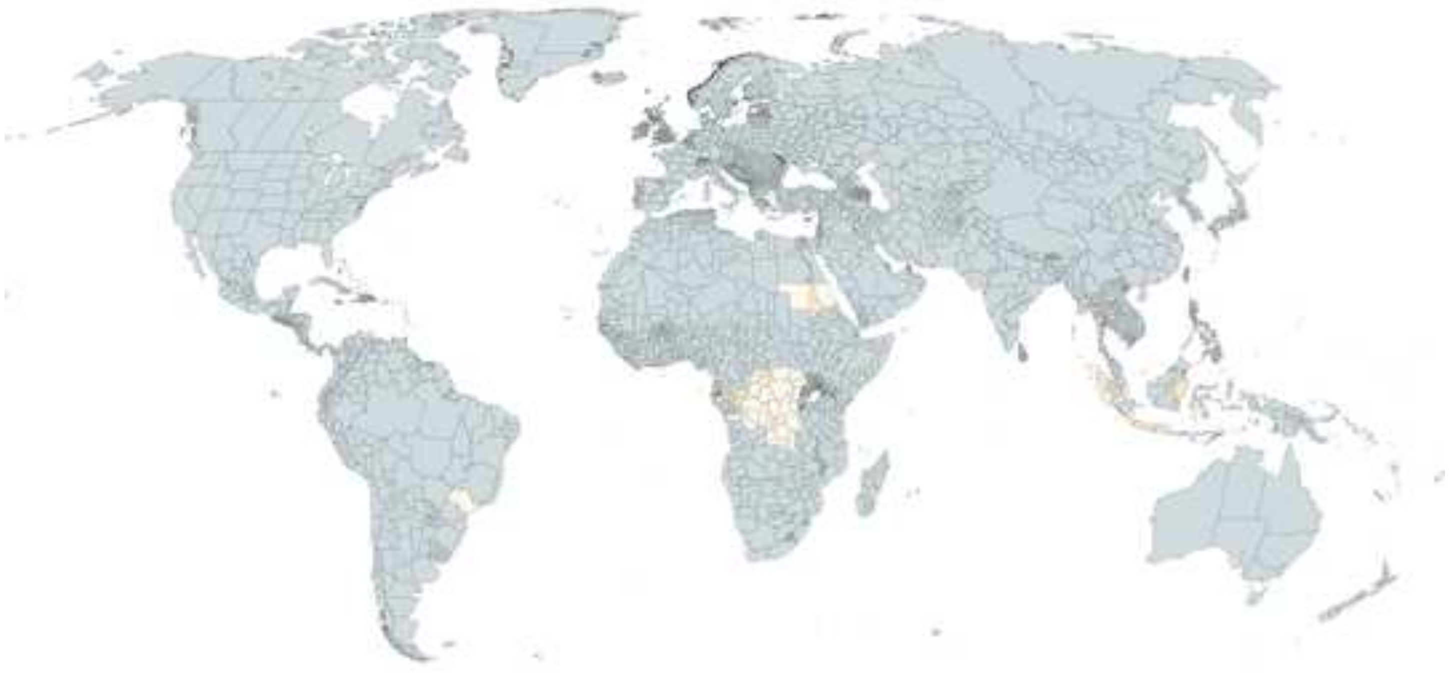

(B)

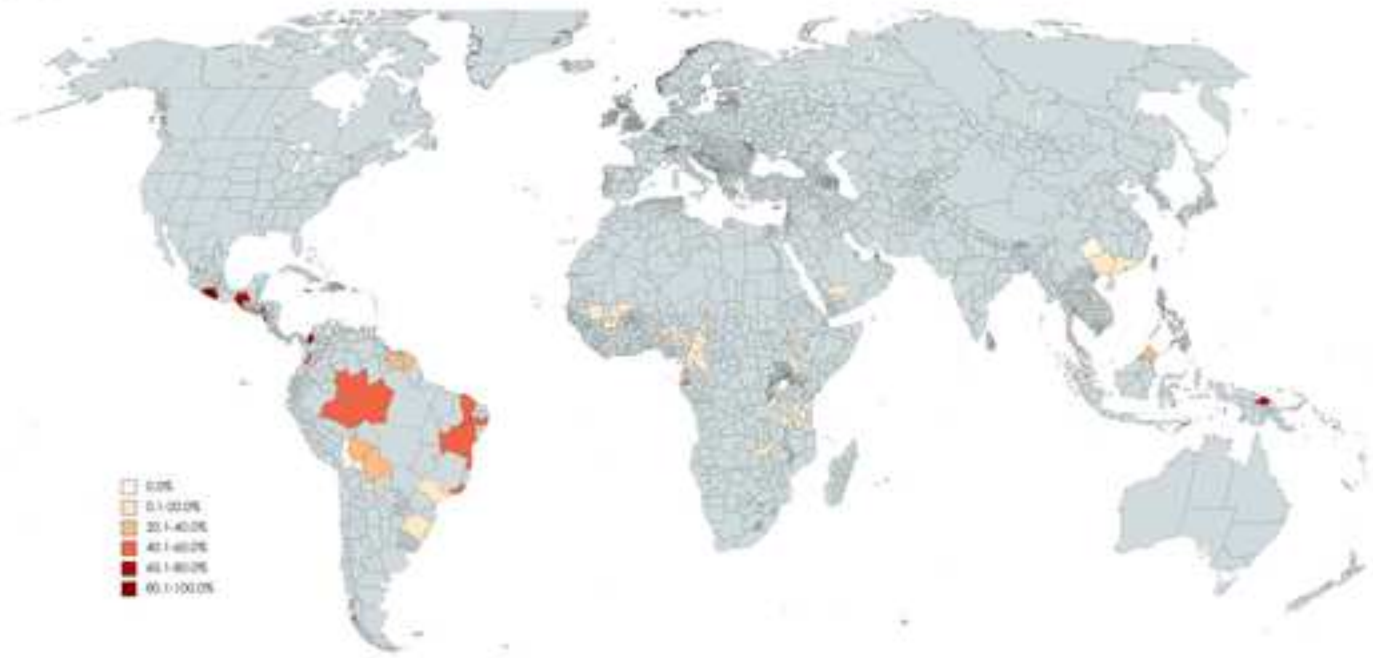

S1 Fig

Click here to access/download
Supporting Information
S1_Fig.tif

S1 Table

Click here to access/download
Supporting Information
S1_Table.docx

S2 Table

Click here to access/download
Supporting Information
S2_Table.docx

S3 Table

Click here to access/download
Supporting Information
S3_Table.docx

S4 Table

Click here to access/download
Supporting Information
S4_Table.docx

S5 Table

Click here to access/download
Supporting Information
S5_Table.docx
