# Supplementary material for: Global seroprevalence of Zika virus in asymptomatic individuals: A systematic review
Source: PLoS Negl Trop Dis. 2024 Apr 17;18(4):e0011842. doi: 10.1371/journal.pntd.0011842 (PMC11057727; doi:10.1371/journal.pntd.0011842)
Supplement: Attachment — Submitted filename: Review comments.docx [file pntd.0011842.s008.docx]

**Global seroprevalence of Zika virus in asymptomatic individuals. A systematic review**

This systematic review paper provides a good overview of the Zika virus, how it spreads, and its genealogy. Provides information on the distribution of the seroprevalence from the past to recent years, and gives information on the regions where the virus has spread. In addition, the paper explains why it is necessary to implement standardized research/protocol on Zika virus. I have the following minor comments to the authors. Additional comments are embedded within the manuscript.pdf file.

Review comments:

1- Provide references for statements in lines 71 and 72-74.
2- Lines 91-101, in the MeSH terms, why was flaviviruses not added to the search terms?
3- Lines 111-114, exclusion criteria, why were studies reporting flaviviruses not included in the exclusion criteria yet in Fig1 they were among the exclusion?
4- Line 121, data abstraction, were raw data (individual data) requested from the authors of the included studies for further detailed analyses?
5- Lines 137-138, studies were scored as 7-9 (low risk), 4-6 (moderate risk), and 0-3 (high risk), how were studies scoring 10 categorized?
6- In table 1, total number of studies per country was reported as 91, and total number of studies worldwide 84. Explain this difference.
7- For the maps, the clarity of the maps needs to be improved. Webpage reference needs to follow journal citation style.
8- Line 202, the reporting of the prevalence need to be standardized e.g., xx/1000 people or similar standardization.
