# Supplementary material for: Global seroprevalence of Zika virus in asymptomatic individuals: A systematic review
Source: PLoS Negl Trop Dis. 2024 Apr 17;18(4):e0011842. doi: 10.1371/journal.pntd.0011842 (PMC11057727; doi:10.1371/journal.pntd.0011842)
Supplement: Attachment — Submitted filename: Letter-Answer to reviewers.docx [file pntd.0011842.s010.docx]

**Mahidol University**

Faculty of Medical Technology

999 Phutthamonthon 4 Rd, Salaya, Phutthamonthon

Nakhon Pathom, 73170, Thailand

E-mail: marielasaba@gmail.com

March 5^th^, 2024

**Ref:** Responses to the review comments on the manuscript titled " Global seroprevalence of Zika virus in asymptomatic individuals: A systematic review"

Dear Editors,

We are very grateful to *PLOS Neglected Tropical Diseases* for considering this paper and for the helpful and interesting comments provided by the reviewers. We have addressed all reviewer comments and modified the manuscript accordingly.

To ensure clarity in our responses, we have included the reviewers’ comments below and appended our answers to each comment.

Please inform us if you require any additional information.

Yours sincerely,

Paola Mariela Saba Villarroel, Ph.D.

**Reviewer 1**

- We modified Jan 2020 to Jan 2000 in Line 239

**Reviewer 2**

***Minor comments***

**Abstract
In the abstract part, no need any citation, the authors cited Hoy et al., 2012, better to remove it.**

- We removed the citation and changed to “a tool to assess the risk of study bias in prevalence studies” (line 27).

**Introduction
Line 70: “The Eastern Mediterranean Region is the only exception”, this statement is not clear, Certainly! The author should elaborate further to provide clarity and ensure that their statement is easily understandable. Clear explanations are essential for effective communication.**

- We changed the sentence to: “The Eastern Mediterranean Region stands as the only region without documented autochthonous ZIKV transmission” (line 71-72).

**Methods
The authors of the MS did not mention the Web of Science Core Collection or the Cochrane Library as databases for searching articles. Instead, they focused on other sources. However, it’s worth noting that these databases are widely used for academic research and could provide valuable information for future studies.**

- Thank you very much for your comment. We also conducted searches on Web of Science. However, we did not mention it to simplify the manuscript, as the articles were already selected from other databases. We are confident that the collected studies include all the articles meeting the inclusion criteria.

**Why the authors excluded research involving travelers, migrants? As you know travelers and migrants play a crucial role in the transmission of Zika disease due to their mobility. When people move across borders or travel between regions, they can inadvertently carry the virus with them, potentially introducing it to new areas. This movement facilitates the spread of Zika and other infectious diseases globally. It’s essential to consider these population dynamics when addressing public health measures and disease prevention strategies.**

- We did not include travelers or migrants because we aimed to depict the true seroprevalence of the population residing in a specific environment, particularly autochthonous Zika infection. The inclusion of travelers and migrants would have biased our results.

**Results
Line 226 ZIKV strains used for methodology, 36 out of 48 employed the Asian lineage, while 11 studies used the African lineage, and 1 Asian and African lineages.  The authors did not include phylogeny data in their analysis. However, they need to address how they compare the Asian lineage and African lineages of the Zika virus.**

- In this paragraph, we analyzed the methodology (seroneutralization) and not virus isolation. In seroneutralization tests, different strains (African or Asian) are used. The results may vary according to the strain used and the lineage circulating in each country or region.

**Reviewer 3**

***Minor comments:***

**Provide references for statements in lines 71 and 72-74.**

- The reference 5 is the same for the entire paragraph. This is why we did not include the reference in lines 71-74.

**Lines 91-101, in the MeSH terms, why was flaviviruses not added to the search terms?**

- We did not include flaviviruses in our search because a large number of articles unrelated to Zika virus were found, which complicated the search.

**Lines 111-114, exclusion criteria, why were studies reporting flaviviruses not included in the exclusion criteria yet in Fig1 they were among the exclusion?**

- Thank you very much for the comment. We added the sentence “studies without differentiation between flaviviruses” in the exclusion criteria.

**Line 121, data abstraction, were raw data (individual data) requested from the authors of the included studies for further detailed analyses?**

- We collected the data from each publication. Therefore, we changed raw to “abstracted” as suggested in line 148.

**Lines 137-138, studies were scored as 7-9 (low risk), 4-6 (moderate risk), and 0-3 (high risk), how were studies scoring 10 categorized?**

- Thank you very much for your comment and we are sorry for the mistake. We changed the score of low risk to 7-10.

**In table 1, total number of studies per country was reported as 91, and total number of studies worldwide 84. Explain this difference.**

- We added an explanation to the table: “84 studies in total: 4 studies including more than one country”.

**For the maps, the clarity of the maps needs to be improved. Webpage reference needs to follow journal citation style.**

- The map references were changed to: World Map - Simple | Create a custom map. In: MapChart [Internet]. [cited 5 Mar 2024]. Available: <https://mapchart.net/world>.

World Map - Advanced | MapChart. [cited 5 Mar 2024]. Available: https://www.mapchart.net/world-advanced.html

**Line 202, the reporting of the prevalence need to be standardized e.g., xx/1000 people or similar standardization.**

- In this part, the numbers represent the sample sizes of the studies analyzed, not the seroprevalence. “Sample sizes ranged widely, from 88 to 5,880 individuals per study”.

**What was the age definition of a child as this may vary from country to country.**

- The age definition is mentioned in line 125. Children under 13 years old. As it varied from study to study, individuals under 13 years old were categorized by us as children.
